# Graded hypoellipticity of BGG sequences

**DOI:** 10.1007/s10455-022-09870-0

**Published:** 2022-09-02

**Authors:** Shantanu Dave, Stefan Haller

**Affiliations:** 1 Faculty of Mathematics, University of Vienna, Oskar-Morgenstern-Platz 1, 1090 Vienna, Austria; 2 Department of Mathematics, University of Vienna, Oskar-Morgenstern-Platz 1, 1090 Vienna, Austria

**Keywords:** Filtered manifold, Pseudodifferential operator, Hypoelliptic operator, Rockland operator, Hypoelliptic sequence, Rockland sequence, BGG sequence, Rumin–Seshadri operator, Engel structure, Generic rank two distribution in dimension five, Primary 58J40, Secondary 58A30, 58A14, 58J10

## Abstract

This article studies hypoellipticity on general filtered manifolds. We extend the Rockland criterion to a pseudodifferential calculus on filtered manifolds, construct a parametrix and describe its precise analytic structure. We use this result to study Rockland sequences, a notion generalizing elliptic sequences to filtered manifolds. The main application that we present is to the analysis of the Bernstein–Gelfand–Gelfand (BGG) sequences over regular parabolic geometries. We do this by generalizing the BGG machinery to more general filtered manifolds (in a non-canonical way) and show that the generalized BGG sequences are Rockland in a graded sense.

## Introduction and main results

Elliptic operators and their analysis touch a large number of problems in geometry, analysis, topology, and physics. At the heart of their wide applicability are three simple reasons:

1. There is a big supply of natural, invariant elliptic operators such as the Laplace and Dirac operators, which encode Riemannian and other geometry.
2. Elliptic operators are defined by invertibility of their “highest order” term. However, this suffices to guarantee that they are invertible up to a smoothing error. This approximate inverse is known as a parametrix, and it ensures many of the usual analytic properties of elliptic operators, such as their Fredholmness on compact manifolds.
3. The heat kernel asymptotic for positive elliptic operators also encodes several important invariants of geometry and topology.

The purpose of this article is to show that in many other geometric situations there is a large supply of natural hypoelliptic operators which admit a nice parametrix in a suitable calculus [17, 52, 79]. These operators include the curved Bernstein–Gelfand–Gelfand (BGG) operators on regular parabolic geometries [11] and Rumin’s complexes [65–67, 69] on Carnot–Carathéodory (C–C) manifolds. The heat kernel asymptotics for positive differential operators in this class resembles the elliptic case, as has been shown in another article [24]. This class of hypoelliptic operators therefore enjoys the same properties as formulated above for elliptic operators.

Historically, finding geometric hypoelliptic operators has been a dream. In the few cases where they are known they lead to striking results, for instance the Connes–Moscovici [21] index formula for the transverse signature operators on foliations, or Julg–Kasparov’s [48] proof of the Baum–Connes conjecture for discrete subgroups of ${\text {SU}}(n,1)$. In both the examples above, the hypoellipticity was known through the Heisenberg calculus, see [3, 59, 73]. Another substantial source of hypoelliptic operators is Hörmander’s celebrated sum of squares theorem [47].

There are many problems in geometry and operator theory, for example the extension of the results by Connes–Moscovici and Julg–Kasparov mentioned above, where the underlying manifold is a filtered manifold and the natural representative of a K-homology class is expected to be a geometric hypoelliptic operator, see [2, 18, 76, 77, 81] for instance. The K-homology class represented by the classical BGG sequences on generalized flag varieties are expected to play a crucial role in extending Julg and Kasparov’s approach to higher rank Lie groups. We will construct similar hypoelliptic sequences for a large class of filtered manifolds.

To obtain precise analytic properties of a hypoelliptic operator, we want its parametrix to be in a suitable pseudodifferential calculus. This will, for instance, provide precise maximal hypoellipticity estimates in the corresponding Sobolev spaces. Such a pseudodifferential calculus for filtered manifold was first described in Melin [52]. Melin’s original preprint remains unpublished. A new geometric approach to the calculus was developed in van Erp and Yuncken [79]. We shall follow [79] for definiteness, although arguably both methods [52, 79] very likely produce the same calculus. Van Erp and Yuncken’s approach is based on the construction of a Heisenberg tangent groupoid for filtered manifolds [15, 41, 54, 78]. We will refer to the calculus as Heisenberg calculus. The construction of the Heisenberg calculus follows the geometrical insights of Debord and Skandalis [27] into classical pseudodifferential calculus using tangent groupoids [20, 45, 64]. The operators in the Heisenberg calculus are classical in the sense that they admit local homogeneous expansions. Our main analytic result shows that operators satisfying a pointwise Rockland condition admit a parametrix in the Heisenberg calculus.

In the remaining part of this introductory section, we will outline the main results and hint at their proofs.

### Filtered manifolds and their osculating groups

A natural structure available on every smooth manifold is its Lie algebra of vector fields. Various geometric structures on smooth manifolds can be described in terms of a filtration on the tangent bundle which is compatible with the Lie bracket of vector fields. This compatibility is subsumed in the concept of a filtered manifold. A filtered manifold is a smooth manifold *M* together with a filtration of its tangent bundle by smooth subbundles,

1 $$\begin{aligned} TM=T^{-r}M\supseteq \cdots \supseteq T^{-2}M\supseteq T^{-1}M\supseteq T^0M=0, \end{aligned}$$

which is compatible with the Lie bracket of vector fields in the sense that $[X,Y]\in \Gamma ^\infty (T^{p+q}M)$ for all $X\in \Gamma ^\infty (T^pM)$ and $Y\in \Gamma ^\infty (T^qM)$.

To each point *x* in a filtered manifold *M* one can assign a simply connected nilpotent Lie group ${\mathcal {T}}_xM$, called the osculating group at *x*, which can be regarded as a non-commutative analogue of the tangent space at *x*. Its Lie algebra is

$$\begin{aligned} {\mathfrak {t}}_xM:={{\,\mathrm{gr}\,}}(T_xM):=\bigoplus _pT_x^pM/T_x^{p+1}M \end{aligned}$$

with the (Levi) bracket induced from the Lie bracket of vector fields. The osculating algebras combine to form a smooth bundle of graded nilpotent Lie algebras $\mathfrak tM$ over *M*, called the bundle of osculating algebras. Correspondingly, the osculating groups combine to form a smooth bundle of simply connected nilpotent Lie groups $\mathcal {T}M$ over *M*, called the bundle of osculating groups. These play a crucial role in the analysis on filtered manifolds.

#### Remark 1.1

There are various conventions on choosing orders/degrees on filtrations and gradings. Van Erp and Yuncken [78, 79], for instance, assign positive values to gradings. We follow the convention prevalent in parabolic geometry, see, for example, [10, 55]. In this context, the choice of negative degree/order is a well-established convention.

To describe simple examples, suppose ${\mathfrak {n}}={\mathfrak {n}}_{-r}\oplus \cdots \oplus {\mathfrak {n}}_{-1}$ is a graded nilpotent Lie algebra, and let *N* be a Lie group with Lie algebra ${\mathfrak {n}}$. Then, the filtration ${\mathfrak {n}}={\mathfrak {n}}^{-r}\supseteq \cdots \supseteq {\mathfrak {n}}^{-1}\supseteq {\mathfrak {n}}^0=0$, with ${\mathfrak {n}}^p:=\bigoplus _{p\le q}{\mathfrak {n}}_q$, determines a left invariant filtration of *TN* which turns *N* into a filtered manifold with (locally) trivial bundle of osculating algebras and typical fiber ${\mathfrak {n}}$.

Let us mention a few geometric structures that can be described as filtered manifolds with special osculating algebras. By Frobenius’ theorem, foliated manifolds can equivalently be described as filtered manifolds with abelian, but non-trivially graded osculating algebras. A contact manifold is just a filtered manifold with osculating algebras isomorphic to the Heisenberg algebra. According to Darboux’s theorem, the filtration on a contact manifold is even locally diffeomorphic to the left invariant filtration on the Heisenberg group. Engel structures on 4-manifolds provide further examples of filtered manifolds that admit local normal forms, cf. [60, 80] and Example 4.19. A generic rank two distribution in dimension five [7, 10, 14, 23, 71] is a filtered manifold with osculating algebras isomorphic to a particular (generic) 5-dimensional graded nilpotent Lie algebra, see Example 4.21. Most regular normal parabolic geometries can equivalently be described as filtered manifolds with prescribed osculating algebras, see [10, Proposition 4.3.1]. These include generic rank two distributions in dimension five, generic rank three distributions in dimension six [5], and quaternionic contact structures [4].

Heisenberg manifolds [59] constitute a well studied class of filtered manifolds for which the bundle of osculating algebras need not be locally trivial. They occur naturally as boundaries of complex manifolds. Equiregular Carnot–Carathéodory (C–C) manifolds [40] give rise to filtered manifolds which are often assumed to be bracket generating in the sense that $T^{-p}M$ is spanned by iterated Lie brackets of sections in $T^{-1}M$ which are of length at most *p*.

### Analytic results

In this paper, we will study operators on filtered manifolds and provide a criterion for their hypoellipticity. Our main analytic results are based on the Heisenberg calculus for filtered manifolds. This calculus can be obtained using the Heisenberg tangent groupoid [15, 41, 54, 78, 79] and the idea of essential homogeneity introduced in [27]. Although the calculus in [79] is described for scalar operators it can easily be generalized to operators acting between sections of vector bundles. The goal is to obtain a general Rockland type theorem in such a pseudodifferential calculus. In addition to using the Heisenberg calculus, the proof of this theorem also builds upon harmonic analysis by Christ et al. [17] and arguments due to Ponge [59].

Let us briefly describe the Heisenberg calculus and the related setup. For two vector bundles *E* and *F* over a filtered manifold *M*, and any complex number *s*, we let $\Psi ^s(E,F)$ denote the class of all pseudodifferential operators of Heisenberg order at most *s*. These are continuous operators $\Gamma ^\infty _c(E)\rightarrow \Gamma ^\infty (F)$ which extend continuously to pseudolocal operators on distributional sections, $\Gamma ^{-\infty }_c(E)\rightarrow \Gamma ^{-\infty }(F)$. They can be characterized as operators with a Schwartz kernel that admits an extension to the Heisenberg tangent groupoid which is essentially homogeneous of order *s*. This extends the Heisenberg filtration on differential operators. More precisely, a differential operator has Heisenberg order at most $k\in {\mathbb {N}}_0$ if and only if it is contained in $\Psi ^k(E,F)$.

An operator $A\in \Psi ^s(E,F)$ has a Heisenberg principal cosymbol $\sigma _x^s(A)\in \Sigma ^s_x(E,F)$ at every point $x\in M$. Here $\Sigma ^s_x(E,F)$ denotes the space of regular distributional volume densities on the osculating group ${\mathcal {T}}_xM$ with values in $\hom (E_x,F_x)$ which are essentially homogeneous of order *s*, modulo smooth volume densities. This cosymbol extends the Heisenberg principal (co)symbol of differential operators on filtered manifolds. The basic properties of this operator class and the Heisenberg principal cosymbol are summarized in Proposition 3.1.

Let $\pi :{\mathcal {T}}_xM\rightarrow U({\mathcal {H}})$ be a non-trivial irreducible unitary representation of the osculating group on a Hilbert space ${\mathcal {H}}$, and let ${\mathcal {H}}_\infty$ denote the subspace of smooth vectors. If $A\in \Psi ^s(E,F)$, then ${\bar{\pi }}(\sigma _x^s(A))$ is a well-defined closed unbounded operator on ${\mathcal {H}}$, restricting to a map ${\bar{\pi }}(\sigma ^s_x(A)):{\mathcal {H}}_\infty \otimes E_x\rightarrow {\mathcal {H}}_\infty \otimes F_x$. The operator *A* is said to satisfy the Rockland [62] condition if ${\bar{\pi }}(\sigma ^s_x(A))$ is injective on ${\mathcal {H}}_\infty \otimes E_x$ for all non-trivial irreducible unitary representations $\pi$ of ${\mathcal {T}}_xM$ and every $x\in M$.

For left invariant differential operators on graded nilpotent Lie groups, Helffer and Nourrigat [43] proved that the harmonic analytic Rockland condition implies hypoellipticity, thus confirming a conjecture of Rockland’s [62] for the Heisenberg group. Christ et al. [17] constructed a pseudodifferential operator calculus on graded nilpotent Lie groups and proved that the pointwise Rockland condition implies the existence of a parametrix in their calculus. For Heisenberg manifolds with varying osculating algebras such a result has been obtained by Ponge [59]. As mentioned already, Melin [52] constructed a pseudodifferential calculus on general filtered manifolds and used it construct a parametrix for scalar Rockland differential operators. We will prove the following general Rockland type result for systems of pseudodifferential operators on general filtered manifolds.

#### Theorem A

Let *E* and *F* be two vector bundles over a filtered manifold *M* and suppose $A\in \Psi ^s(E,F)$ satisfies the Rockland condition. Then, there exists a properly supported left parametrix $B\in \Psi ^{-s}_\text {prop}(F,E)$; that is, $BA-{{\,\mathrm{id}\,}}$ is a smoothing operator.

As a consequence, every operator $A\in \Psi ^s(E,F)$ satisfying the Rockland condition is hypoelliptic; that is, if $\psi$ is a compactly supported distributional section of *E* such that $A\psi$ is smooth on an open subset *U* of *M*, then $\psi$ was smooth on *U*. Over closed manifolds this implies that $\ker (A)$ is a finite dimensional subspace of $\Gamma ^\infty (E)$, see Theorem 3.11.

Combining Theorem A with a result of Christ et al., see [17, Theorem 6.1], we construct, for each complex number *s*, an operator $\Lambda _s\in \Psi ^s(E)$ which is invertible mod smoothing operators, see Lemma 3.13. This permits us to introduce a Heisenberg Sobolev scale, see Proposition 3.17, and allows us to formulate more refined regularity statements, including maximal hypoelliptic estimates, for operators satisfying the Rockland condition, see Corollary 3.20.

We will use Theorem A to analyze Rockland sequences. A sequence of operators,

$$\begin{aligned} \cdots \rightarrow \Gamma ^\infty (E_{i-1})\xrightarrow {A_{i-1}}\Gamma ^\infty (E_i)\xrightarrow {A_i}\Gamma ^\infty (E_{i+1})\rightarrow \cdots , \end{aligned}$$

where $A_i\in \Psi ^{s_i}(E_i,E_{i+1})$ will be called Rockland sequence if the corresponding principal symbol sequence is exact in every non-trivial irreducible unitary representation $\pi :{\mathcal {T}}_xM\rightarrow U({\mathcal {H}})$ at each $x\in M$, that is, the sequence

$$\begin{aligned} \cdots \rightarrow {\mathcal {H}}_\infty \otimes E_{x,i-1}\xrightarrow {{\bar{\pi }}(\sigma ^{s_{i-1}}_x(A_{i-1}))}{\mathcal {H}}_\infty \otimes E_{x,i}\xrightarrow {{\bar{\pi }}(\sigma ^{s_i}_x(A_i))}{\mathcal {H}}_\infty \otimes E_{x,i+1}\rightarrow \cdots \end{aligned}$$

is exact. On trivially filtered manifolds this definition reduces to the well-known concept of elliptic sequences. To study these sequences, we consider formal adjoints $A_i^*\in \Psi ^{{\bar{s}}}(E_{i+1},E_i)$ with respect to standard $L^2$ inner products on $\Gamma ^\infty (E_i)$. Theorem A implies that $(A_{i-1}^*,A_i)$ is hypoelliptic, and more refined regularity statements, including maximal hypoelliptic estimates, can be formulated using the Heisenberg Sobolev scale.

On closed manifolds, in case the Rockland sequence forms a complex, i.e., $A_iA_{i-1}=0$, it is convenient to use suitable Sobolev adjoints, $A_i^\sharp$ of $A_i$. These adjoints are constructed such that $A_{i-1}A_{i-1}^\sharp$ and $A_i^\sharp A_i$ have the same Heisenberg order and thus $B_i:=A_{i-1}A_{i-1}^\sharp +A_i^\sharp A_i$ is a Rockland operator. We obtain a Hodge decomposition

$$\begin{aligned} \Gamma ^\infty (E_i)={{\,\mathrm{img}\,}}(A_{i-1})\oplus \ker (B_i)\oplus {{\,\mathrm{img}\,}}(A_i^\sharp ) \end{aligned}$$

where $\ker (B_i)=\ker (A_{i-1}^\sharp )\cap \ker (A_i)$. In particular, each cohomology class has a unique harmonic representative, that is, $\ker (A_i)/{{\,\mathrm{img}\,}}(A_{i-1})=\ker (B_i)$.

For Rockland complexes of differential operators of positive order, one can alternatively follow the Rumin–Seshadri approach [70] and consider

2 $$\begin{aligned} \Delta _i:=(A_{i-1}A_{i-1}^*)^{a_{i-1}}+(A_i^*A_i)^{a_i} \end{aligned}$$

where the numbers $a_i\in {\mathbb {N}}$ are chosen such that $\kappa :=s_{i-1}a_{i-1}=s_ia_i$. Then, $\Delta _i$ is a differential operator of order at most $2\kappa$ which satisfies the Rockland condition. We obtain a similar Hodge decomposition, namely,

$$\begin{aligned} \Gamma ^\infty (E_i)={{\,\mathrm{img}\,}}(A_{i-1})\oplus \ker (\Delta _i)\oplus {{\,\mathrm{img}\,}}(A_i^*), \end{aligned}$$

where $$\ker (A_i)/{{\,\mathrm{img}\,}}(A_{i-1})=\ker (\Delta _i)=\ker (A_{i-1}^*)\cap \ker (A_i)$$.

In order to study the generalized BGG sequences, we will construct below, the analysis needs to be adapted to a setup where the operators act on sections of filtered vector bundles, cf. Rumin’s concept of Carnot–Carathéodory (C–C) ellipticity in [67, 69]. For any two filtered vector bundles *E* and *F*, we consider a class of operators, ${\tilde{\Psi }}^s(E,F)$, which will be called pseudodifferential operators of graded Heisenberg order *s*. If we identify *E* and *F* with the associated graded using splittings of the filtrations, then an operator $A\in {\tilde{\Psi }}^s(E,F)$ can be considered as a matrix with entries $A_{qp}\in \Psi ^{s+q-p}({{\,\mathrm{gr}\,}}_p(E),{{\,\mathrm{gr}\,}}_q(F))$. The graded Heisenberg principal cosymbol, ${\tilde{\sigma }}^s_x(A)$, can be defined as the matrix obtained by taking the (ordinary) Heisenberg principal cosymbol of each entry, that is,

$$\begin{aligned} {\tilde{\sigma }}^s_x(A)=\sum _{p,q}\sigma ^{s+q-p}_x(A_{qp}). \end{aligned}$$

Neither the operator class ${\tilde{\Psi }}^s(E,F)$, nor the graded Heisenberg principal cosymbol ${\tilde{\sigma }}^s_x(A)$ depend on the choice of splittings.

An operator $A\in {\tilde{\Psi }}^s(E,F)$ is called graded Rockland operator if ${\bar{\pi }}({\tilde{\sigma }}^s_x(A)):{\mathcal {H}}_\infty \otimes {{\,\mathrm{gr}\,}}(E_x)\rightarrow {\mathcal {H}}_\infty \otimes {{\,\mathrm{gr}\,}}(F_x)$ is injective for all non-trivial irreducible unitary representations $\pi :{\mathcal {T}}_xM\rightarrow U({\mathcal {H}})$ and all $x\in M$. Similarly, a graded Rockland sequence is defined to be a sequence of operators such that its graded Heisenberg principal symbol sequence is exact in each non-trivial irreducible unitary representation of ${\mathcal {T}}_xM$. Theorem A has a graded analogue, and all the analysis mentioned above generalizes to this graded setup, see Sect. 5. Even if a graded Rockland sequence is made of differential operators, its analysis requires conjugation by a pseudodifferential operator in the calculus, cf. [67, 69]. This is another reason why we need the generality of Theorem A to analyze generalized BGG sequences.

### Construction of Rockland sequences

In this paper will shall construct several examples of Rockland sequences. The most basic sequence we will consider is the de Rham sequence associated with a linear connection $\nabla$ on a filtered vector bundle *E* over a filtered manifold *M*. This can be characterized as the unique extension of $\nabla$,

3 $$\begin{aligned} \cdots \rightarrow \Omega ^{k-1}(M;E)\xrightarrow {d_{k-1}^\nabla }\Omega ^k(M;E)\xrightarrow {d_k^\nabla }\Omega ^{k+1}(M;E)\rightarrow \cdots , \end{aligned}$$

such that the Leibniz rule $d^\nabla (\alpha \wedge \psi )=d\alpha \wedge \psi +(-1)^k\alpha \wedge d^\nabla \psi$ holds for all $\alpha \in \Omega ^k(M)$ and $\psi \in \Omega ^*(M;E)$, cf. [37, Section 7.14]. Here, we use the notation $\Omega ^k(M;E)=\Gamma ^\infty (\Lambda ^kT^*M\otimes E)$ for the space of *E*-valued differential forms.

We assume that $\nabla$ is filtration-preserving; that is to say, we assume $\nabla _X\psi \in \Gamma ^\infty (E^{p+q}M)$ for all $X\in \Gamma ^\infty (T^pM)$ and $\psi \in \Gamma ^\infty (E^q)$. Then, all operators in the sequence (3) are of graded Heisenberg order at most zero with respect to the induced filtration on the vector bundles $\Lambda ^kT^*M\otimes E$. We will, furthermore, assume that the curvature [37, Section 7.15] of $\nabla$ is contained in filtration degree one, that is, we assume $F^\nabla _x(X_1,X_2)\psi \in E_x^{p_1+p_2+p+1}$ for all $X_i\in T_x^{p_i}M$ and $\psi \in E^p_x$. Linear connections of this kind exist on every filtered vector bundle. If *E* is trivially filtered, then all linear connections on *E* satisfy the two assumptions. In general, using a splitting of the filtration to identify *E* with its associated graded, ${{\,\mathrm{gr}\,}}(E)=\bigoplus _pE^p/E^{p+1}$, each linear connection preserving the grading on ${{\,\mathrm{gr}\,}}(E)$ will satisfy the two assumptions. Moreover, all tractor bundles associated with regular parabolic geometries come equipped with a natural linear connection satisfying these assumptions, see Sect. 4.6.

We have the following generalization of a result of Rumin’s for the de Rham complex on C–C manifolds, cf. [69, Theorem 5.2] and [67, Theorem 3].

#### Theorem B

Let *E* be a filtered vector bundle over a filtered manifold *M* and suppose $\nabla$ is a filtration-preserving linear connection on *E* such that its curvature is contained in filtration degree one. Then the de Rham sequence in (3) is a graded Rockland sequence.

Essentially, Theorem B follows from the fact that the Lie algebra cohomology $H^*({\mathfrak {g}};{\mathcal {H}}_\infty )$ vanishes for every finite dimensional nilpotent Lie algebra ${\mathfrak {g}}$ and its representation on the space of smooth vectors ${\mathcal {H}}_\infty$ associated with any non-trivial irreducible unitary representation of the corresponding simply connected nilpotent Lie group on a Hilbert space ${\mathcal {H}}$. This will be established in Sect. 4.4, see Proposition 4.15.

To construct new sequences, we follow Čap et al., see [11], and consider a Kostant type codifferential. By this we mean a sequence of filtration-preserving vector bundle homomorphisms,

$$\begin{aligned} \cdots \leftarrow \Omega ^{k-1}(M;E)\xleftarrow {\delta _k}\Omega ^k(M;E)\xleftarrow {\delta _{k+1}}\Omega ^{k+1}(M;E)\leftarrow \cdots , \end{aligned}$$

satisfying $\delta _k\delta _{k+1}=0$ and two more conditions formulated in Definition 4.6. Assuming $\delta _k$ to have locally constant rank, we obtain smooth vector bundles $\ker (\delta _k)$, ${{\,\mathrm{img}\,}}(\delta _{k+1})$, and ${\mathcal {H}}_k:=\ker (\delta _k)/{{\,\mathrm{img}\,}}(\delta _{k+1})$, which are filtered in a natural way. We let ${\bar{\pi }}_k:\ker (\delta _k)\rightarrow {\mathcal {H}}_k$ denote the natural vector bundle projection.

Using the BGG machinery [8, 11–13], we will see that there exist operators analogous to the splitting operators in parabolic geometry, see [12, Theorem 2.4]. More precisely, there exists a unique differential operator ${\bar{L}}_k:\Gamma ^\infty ({\mathcal {H}}_k)\rightarrow \Omega ^k(M;E)$ such that $\delta _k{\bar{L}}_k=0$, ${\bar{\pi }}_k{\bar{L}}_k={{\,\mathrm{id}\,}}$, and $\delta _{k+1}d^\nabla _k{\bar{L}}_k=0$. These operators ${\bar{L}}_k$ are of graded Heisenberg order zero and permit defining a sequence of differential operators of graded Heisenberg order zero,

4 $$\begin{aligned} \cdots \rightarrow \Gamma ^\infty ({\mathcal {H}}_{k-1})\xrightarrow {{\bar{D}}_{k-1}}\Gamma ^\infty ({\mathcal {H}}_k)\xrightarrow {{\bar{D}}_k}\Gamma ^\infty ({\mathcal {H}}_{k+1})\rightarrow \cdots , \end{aligned}$$

by setting $${\bar{D}}_k:={\bar{\pi }}_{k+1}d^\nabla _k{\bar{L}}_k$$.

In Sect. 4.5, we will establish the following result, see Corollary 4.18.

#### Theorem C

The operators in (4) form a graded Rockland sequence.

A codifferential $\delta$ of maximal rank exists, provided the dimension of the Lie algebra cohomology $H^*({\mathfrak {t}}_xM;{{\,\mathrm{gr}\,}}(E_x))$ is locally constant in *x*. Note that the curvature assumption on $\nabla$ implies that ${{\,\mathrm{gr}\,}}(E_x)$ becomes a graded representation of the graded nilpotent Lie algebra ${\mathfrak {t}}_xM$, see Lemma 4.14. In this case the codifferential $\delta _k$ can be constructed using splittings of the filtrations on the bundles $\Lambda ^kT^*M\otimes E\cong {{\,\mathrm{gr}\,}}(\Lambda ^kT^*M\otimes E)=\Lambda ^k{\mathfrak {t}}^*M\otimes {{\,\mathrm{gr}\,}}(E)$ and the adjoint of the fiber-wise Chevalley–Eilenberg differential, $\partial _{k-1}:\Lambda ^{k-1}{\mathfrak {t}}^*M\otimes {{\,\mathrm{gr}\,}}(E)\rightarrow \Lambda ^k{\mathfrak {t}}^*M\otimes {{\,\mathrm{gr}\,}}(E)$, see Remark 4.12 for details. For this codifferential there exists a (non-canonical) isomorphism of smooth vector bundles ${\mathcal {H}}_k\cong H^k(\mathfrak tM;{{\,\mathrm{gr}\,}}(E))=\ker (\partial _k)/{{\,\mathrm{img}\,}}(\partial _{k-1})$ where the latter denotes the vector bundle with fibers $H^k({\mathfrak {t}}_xM;{{\,\mathrm{gr}\,}}(E_x))$.

For tractor bundles associated with regular parabolic geometries, however, there exists a natural choice for $\delta$ which is called *Kostant codifferential* and often denoted by $\partial ^*$. In this case the construction above reduces to the construction of the curved BGG sequences, and the operators ${\bar{L}}_k$ coincide with the well-known splitting operators, see [12, Theorem 2.4] for instance. As an immediate corollary of Theorem C, we thus obtain, cf. Corollary 4.20:

#### Theorem D

All (curved, torsion free) BGG sequences associated with a regular parabolic geometry are graded Rockland sequences.

To prove Theorem C, we shall construct another sequence that, at the principal symbol level, can be combined with the sequence (4) to obtain the de Rham sequence of Theorem B, up to conjugation. The Rockland condition for both components then follows from Theorem B. This construction is closely related to the standard BGG machinery and the approach by Rumin [67, 69].

More precisely, we consider $\Box _k:=d^\nabla _{k-1}\delta _k+\delta _{k+1}d^\nabla _k$, a differential operator of graded Heisenberg order at most zero on $\Omega ^k(M;E)$. The associated graded vector bundle endomorphism ${\tilde{\Box }}_k:={{\,\mathrm{gr}\,}}(\Box _k)$ on ${{\,\mathrm{gr}\,}}(\Lambda ^kT^*M\otimes E)$ is analogous to Kostant’s box operator. Using the fiber-wise projection onto the generalized zero eigenspace of ${\tilde{\Box }}_k$, we obtain a vector bundle projector ${\tilde{P}}_k$ on ${{\,\mathrm{gr}\,}}(\Lambda ^kT^*M\otimes E)$, providing a decomposition of smooth vector bundles

$$\begin{aligned} {{\,\mathrm{gr}\,}}(\Lambda ^kT^*M\otimes E)={{\,\mathrm{img}\,}}({\tilde{P}}_k)\oplus \ker ({\tilde{P}}_k) \end{aligned}$$

such that ${\tilde{\Box }}_k$ is nilpotent on ${{\,\mathrm{img}\,}}({\tilde{P}}_k)$ and invertible on $\ker ({\tilde{P}}_k)$. We will construct two sequences of differential operators of graded Heisenberg order at most zero,

5 $$\begin{aligned} \cdots \rightarrow \Gamma ^\infty ({{\,\mathrm{img}\,}}({\tilde{P}}_{k-1}))\xrightarrow {D_{k-1}}\Gamma ^\infty ({{\,\mathrm{img}\,}}({\tilde{P}}_k))\xrightarrow {D_k}\Gamma ^\infty ({{\,\mathrm{img}\,}}({\tilde{P}}_{k+1}))\rightarrow \cdots \end{aligned}$$

and

6 $$\begin{aligned} \cdots \rightarrow \Gamma ^\infty (\ker ({\tilde{P}}_{k-1}))\xrightarrow {B_{k-1}}\Gamma ^\infty (\ker ({\tilde{P}}_k))\xrightarrow {B_k}\Gamma ^\infty (\ker ({\tilde{P}}_{k+1}))\rightarrow \cdots , \end{aligned}$$

as well as invertible differential operators

$$\begin{aligned} L_k:\Gamma ^\infty ({{\,\mathrm{gr}\,}}(\Lambda ^kT^*M\otimes E))\rightarrow \Omega ^k(M;E), \end{aligned}$$

such that the graded Heisenberg principal symbols are related by

$$\begin{aligned} {\tilde{\sigma }}^0_x(L^{-1}_{k+1}d_k^\nabla L_k)={\tilde{\sigma }}^0_x(D_k)\oplus {\tilde{\sigma }}^0_x(B_k) \end{aligned}$$

at each point $x\in M$. Theorem B readily implies that (5) and (6) are both graded Rockland sequences. Moreover, we will construct an invertible differential operator of graded Heisenberg order zero, $V_k:\Gamma ^\infty ({{\,\mathrm{img}\,}}({\tilde{P}}_k))\rightarrow \Gamma ^\infty ({\mathcal {H}}_k)$, such that $V_{k+1}^{-1}{\bar{D}}_kV_k=D_k$, whence Theorem C.

The construction of the operators announced in the preceding paragraph is based on the observation that there exists a unique filtration-preserving differential operator

$$\begin{aligned} P_k:\Omega ^k(M;E)\rightarrow \Omega ^k(M;E) \end{aligned}$$

characterized by $P_k\Box _k=\Box _kP_k$, $P_k^2=P_k$ and ${{\,\mathrm{gr}\,}}(P_k)={\tilde{P}}_k$. This operator has graded Heisenberg order zero. Using splittings of the filtrations, $S_k:{{\,\mathrm{gr}\,}}(\Lambda ^kT^*M\otimes E)\rightarrow \Lambda ^kT^*M\otimes E$, we define differential operators of graded Heisenberg order zero,

$$\begin{aligned} L_k:=P_kS_k{\tilde{P}}_k+({{\,\mathrm{id}\,}}-P_k)S_k({{\,\mathrm{id}\,}}-{\tilde{P}}_k). \end{aligned}$$

Since ${{\,\mathrm{gr}\,}}(L_k)={{\,\mathrm{id}\,}}$, this differential operator is invertible and its inverse $L^{-1}_k$ is a differential operator of graded Heisenberg order zero too. Moreover, it conjugates the differential projectors into vector bundle projectors, $L^{-1}_kP_kL_k={\tilde{P}}_k$. We will verify that the operators $D_k:={\tilde{P}}_{k+1}L_{k+1}^{-1}d^\nabla _kL_k|_{\Gamma ^\infty ({{\,\mathrm{img}\,}}({\tilde{P}}_k))}$, $B_k:=({{\,\mathrm{id}\,}}-{\tilde{P}}_{k+1})L_{k+1}^{-1}d^\nabla _kL_k|_{\Gamma ^\infty (\ker ({\tilde{P}}_k))}$, and $V_k:={\bar{\pi }}_kL_k|_{\Gamma ^\infty ({{\,\mathrm{img}\,}}({\tilde{P}}_k))}$ with inverse $V_k^{-1}=L_k^{-1}{\bar{L}}_k$ have all the desired properties. The operator $P_k$ is related to the splitting operator ${\bar{L}}_k$ considered above by ${\bar{L}}_k{\bar{\pi }}_k=P_k|_{\ker (\delta _k)}$. On regular parabolic geometries $P_k$ coincides with the composition of (5.1) and (5.2) in [8].

Let us now suppose that the linear connection $\nabla$ is flat. In this case the sequence (3) is known as de Rham complex, $d^\nabla _kd^\nabla _{k-1}=0$, and computes the cohomology of *M* with coefficients in the locally constant sheave provided by the flat connection on *E*. In this case the sequence of operators $L_{k+1}^{-1}d^\nabla _kL_k$ decouples into a Rumin complex and an acyclic subcomplex, cf. [69, Theorem 2.6] or [67, Theorem 1]. More precisely, we have

$$\begin{aligned} L_{k+1}^{-1}d^\nabla _kL_k=D_k\oplus B_k, \end{aligned}$$

and, in particular, $D_kD_{k-1}=0$ as well as $B_kB_{k-1}=0$. In this situation the sequences in (4) and (5) will be called Rumin complexes, for they essentially coincide with complexes on contact [65, 66, 68] and more general Carnot–Carathéodory [67, 69] manifolds which have been introduced by Rumin. We will show that the sequence $B_k$ is conjugate to an acyclic tensorial complex. More precisely, we will see that there exist invertible differential operators of graded Heisenberg order at most zero, $G_k$ acting on $\Gamma ^\infty (\ker ({\tilde{P}}_k))$, such that

$$\begin{aligned} G_{k+1}^{-1}B_kG_k=\partial _k|_{\Gamma ^\infty (\ker ({\tilde{P}}_k))} \end{aligned}$$

where the right hand side denotes the restriction of the Chevalley–Eilenberg differential on ${{\,\mathrm{gr}\,}}(\Lambda ^kT^*M\otimes E)=\Lambda ^k{\mathfrak {t}}^kM\otimes {{\,\mathrm{gr}\,}}(E)$ to the invariant acyclic subbundle $\ker ({\tilde{P}}_k)$, see Theorem 4.17. Summarizing, we obtain:

#### Theorem E

If the linear connection $\nabla$ is a flat, then there exist invertible differential operators of graded Heisenberg order zero, $W_k:\Gamma ^\infty ({\mathcal {H}}_k\oplus \ker ({\tilde{P}}_k))\rightarrow \Omega ^k(M;E)$, such that

$$\begin{aligned} W^{-1}_{k+1}d^\nabla _kW_k={\bar{D}}_k\oplus (\partial _k|_{\Gamma ^\infty (\ker ({\tilde{P}}_k))}). \end{aligned}$$

On a contact manifold, the Rumin complex (4) is Rockland in the ungraded sense. Hypoellipticity of this complex has been established by Rumin [66, Section 3] using results of Helffer and Nourrigat [44]. For generic rank two distributions in dimension five, the Rumin complex is Rockland in the ungraded sense too, see Example 4.21. In general, the Rumin complex will only be Rockland in the graded sense, and the graded analysis in Sect. 5 may be used to study them. For instance, the Rumin complex associated with an Engel structure will only be Rockland in the graded sense, see Example 4.19. On C–C manifolds, Rumin has used the concept of C–C ellipticity, see [69, Definition 5.1] or [67, Section 2], to show that the Rumin complexes are hypoelliptic, see [69, Theorem 5.2] or [67, Theorem 3].

### Motivation and outlook

The work in this paper provides a framework to study filtered manifolds by exploring the analogies to the elliptic case. Classically, the relation between geometry and topology has been successfully studied by analyzing elliptic operators that arise naturally. We hope that the hypoellipticity of the operators considered in this paper will allow relating the geometry of filtered manifolds to global topological properties. We will now mention some directions which have been motivating our investigations.

By hypoellipticity, Rockland operators on closed filtered manifolds are Fredholm and there is a clear candidate for the index formula. To be more specific, suppose *E* and *F* are two vector bundles over a closed filtered manifold, and consider $A\in \Psi ^s(E,F)$ such that *A* and $A^t$ both satisfy the Rockland condition. In this situation, the analysis mentioned above implies that *A* induces a Fredholm operator between appropriate Heisenberg Sobolev spaces, see Corollary 3.23. We expect that the index of this operator can be computed by an index formula similar to van Erp’s in the contact case, see [20, 76]. More precisely, the Rockland condition should guarantee that the Heisenberg principal symbol of *A* represents a *K*-theory class on the non-commutative cotangent bundle, $[\sigma ^s(A)]\in K_0(C^*(\mathcal {T}M))$, and we expect the index formula ${{\,\mathrm{ind}\,}}(A)={{\,\mathrm{t-ind}\,}}(\psi ([\sigma ^s(A)]))$ where $\psi :K_0(C^*(\mathcal {T}M))\rightarrow K^0(T^*M)$ denotes the abstract Connes–Thom isomorphism [19] and ${{\,\mathrm{t-ind}\,}}:K^0(T^*M)\rightarrow {\mathbb {Z}}$ is the topological index map of Atiyah and Singer [1]. More generally, the Heisenberg principal symbol sequence of every Rockland complex should, in a natural way, represent an element in $K_0(C^*(\mathcal {T}M))$ which is mapped to the Euler characteristics of the Rockland complex via ${{\,\mathrm{t-ind}\,}}\circ \psi :K_0(C^*(\mathcal {T}M))\rightarrow {\mathbb {Z}}$. We expect explicit index formulas for various parabolic geometries, similar to van Erp’s formula on contact manifolds, see [77].

It seems promising to try to extend the Weitzenböck formula for the Rumin complex on contact manifolds [65, 66] to other filtered manifolds *M* and combine them with the Hodge decomposition, cf. Corollaries 2.16 and 5.8, to obtain analogues of Bochner’s vanishing result. Assuming non-negative curvature, a Weitzenböck formula should imply that every harmonic section of ${\mathcal {H}}_k$ is parallel. Over closed connected manifolds, the Hodge decomposition would thus yield a bound on the *k*-th Betti number, $b_k(M)\le {{\,\mathrm{rank}\,}}({\mathcal {H}}_k)$. If, moreover, the curvature is strictly positive at one point, one would expect $b_k(M)=0$.

Let us specialize the above remarks to a particular 5-dimensional Cartan geometry and formulate a precise conjecture. To this end, consider a 5-manifold *M* equipped with a generic rank two distribution [7, 14, 23, 71]. More precisely, suppose $T^{-1}M\subseteq TM$ is a distribution of rank two with growth vector (2, 3, 5), that is, Lie brackets of sections of $T^{-1}M$ span a rank three subbundle $T^{-2}M$ of *TM* and triple brackets of sections of $T^{-1}M$ span all of *TM*. Such a filtered manifold can equivalently be described as a regular normal parabolic geometry of type (*G*, *P*) where *G* is the split real form of the exceptional Lie group $G_2$ and *P* is a particular parabolic subgroup. Cartan [14] constructed a curvature tensor $\kappa \in \Gamma ^\infty (S^4(T^{-1}M)^*)$ which is a complete obstruction to local flatness. More precisely, $\kappa$ vanishes if and only if the filtration is locally diffeomorphic to the flat model *G*/*P*. Regarding the curvature $\kappa _x$ as a fourth order polynomial on $T^{-1}_xM$, we call $\kappa _x$ non-negative and write $\kappa _x\ge 0$, if $\kappa _x(X,Y,X,Y)\ge 0$ for all $X,Y\in T_x^{-1}M$. Since the corresponding Rumin complex for the trivial flat line bundle has ${{\,\mathrm{rank}\,}}({\mathcal {H}}_1)=2$, see Example 4.21, we conjecture the following to hold true: If *M* is closed, connected, and $\kappa \ge 0$, then the first Betti number is bounded by $b_1(M)\le 2$. If, moreover, $\kappa _x>0$ in at least one point *x*, then $b_1(M)=0$.

Another application we have in mind concerns the extension of Ponge’s [59] spectral analysis on Heisenberg manifolds to more general filtered manifolds. In [24], building on the analytic results presented here, the heat kernel expansion has been established for formally self-adjoint, non-negative Rockland differential operators on general closed filtered manifolds. As an immediate application of this result, one obtains the detailed structure of complex powers of these operators, and a Weyl’s law for the growth of their eigenvalues. Other applications include a McKean–Singer index formula for Rockland differential operators, and the construction of a non-commutative residue on the algebra of Heisenberg pseudodifferential operators, see [24]. Moreover, this analysis permits to generalize [42] the Rumin–Seshadri analytic torsion [70] to closed filtered manifolds which give rise to ungraded Rumin complexes. Due to the rich structure available on regular parabolic geometries it appears feasible to work out explicit anomaly formulas for the Rumin–Seshadri analytic torsion, expressing to what extent this analytic torsion depends on the $L^2$ inner product used to define the formal adjoints, see (2). We hope that the decomposition of the de Rham complex in Theorem E will prove helpful in establishing a comparison result relating the Rumin–Seshadri analytic torsion of the Rumin complex with the Ray–Singer torsion [61] of the full de Rham complex.

The existence of a regular parabolic geometry of a particular type on a given manifold can often be described equivalently in terms of a differential relation. Formally, these can be solved in terms of homotopy theory. The subtle question is to what extent Gromov’s h-principle [39] holds true for regular parabolic geometries. We anticipate that the proposed generalization of the Rumin–Seshadri analytic torsion has the potential to detect a possible failure of the h-principle. In particular, this might lead to topological obstructions to the existence of regular parabolic geometries on closed manifolds [23], and it might provide a sufficiently strong tool to show that formally homotopic regular parabolic geometries need not be homotopic in general, see [60].

### Organization of the remaining part of the paper

In Sect. 2, we begin by considering differential operators on filtered manifolds. We present a result which states the existence of a parametrix for Rockland differential operators, and discuss several immediate consequences. The Rockland type theorem will be proved in Sect. 3 which contains a more general form of this result. There we recall the Heisenberg pseudodifferential calculus and complement it with a general Rockland type theorem asserting that Rockland pseudodifferential operators admit a parametrix in this calculus. As an application, we introduce a Heisenberg Sobolev scale, and formulate maximal hypoelliptic estimates. We extend the BGG machinery to filtered manifolds in Sect. 4 and show that the BGG sequences are Rockland in a graded sense. Section 5 then provides hypoellipticity for graded Rockland operators by reducing it to the results in Sect. 3. This completes the proof of the claim that BGG sequences are hypoelliptic.

## Hypoelliptic sequences of differential operators

In the present section, we are considering differential operators on filtered manifolds. We present a result which states the existence of a parametrix for Rockland differential operators. Since our main goal is to provide analysis for general BGG type operators, we will note here that the result of this section is inadequate for this purpose in spite of the fact that these sequences are made of differential operators. This is because the vector bundles underlying the BGG sequences are graded and hence they only satisfy a graded version of the pointwise Rockland condition, as shown in Sect. 4. The purpose of this section is to set up notation and provide background for readers not familiar with pseudodifferential operators.

### Differential operators on filtered manifolds

Let *M* be a filtered manifold, cf. (1), and consider the quotient bundle ${\mathfrak {t}}^pM:=T^pM/T^{p+1}M$ with fibers ${\mathfrak {t}}_x^pM=T^p_xM/T^{p+1}_xM$. Recall that the Lie bracket of vector fields induces a tensorial (Levi) bracket ${\mathfrak {t}}^pM\otimes {\mathfrak {t}}^qM\rightarrow {\mathfrak {t}}^{p+q}M$ which turns the associated graded $\mathfrak tM:=\bigoplus _p{\mathfrak {t}}^pM$ into a bundle of graded nilpotent Lie algebras called the *bundle of osculating algebras*. Each fiber ${\mathfrak {t}}_xM=\bigoplus _p{\mathfrak {t}}^p_xM$ is a graded nilpotent Lie algebra which will be referred to as the *osculating algebra at x*. The Lie algebra structure depends smoothly on *x* but is not assumed to be locally trivial, that is, different fibers may be non-isomorphic as Lie algebras. In the literature ${\mathfrak {t}}_xM$ is also known as the symbol algebra of *M* at *x*, see [55, 56, 58].

A filtration on *M* induces a (Heisenberg) filtration on differential operators. If *E* and *F* are smooth vector bundles over *M*, we let ${\mathcal {D}}{\mathcal {O}}(E,F)$ denote the class of all differential operators mapping section of *E* to sections of *F*. A differential operator in ${\mathcal {D}}{\mathcal {O}}(E,F)$ is said to be of *Heisenberg order at most k* if, locally, it can be written as a finite linear combination of operators of the form $\Phi \nabla _{X_m}\cdots \nabla _{X_1}$ where $\Phi \in \Gamma ^\infty (\hom (E,F))$, $\nabla$ is a linear connection on *E*, and $X_i\in \Gamma ^\infty (T^{p_i}M)$ are vector fields such that $-k\le p_m+\cdots +p_1$. Denoting the space of these differential operators by ${\mathcal {D}}{\mathcal {O}}^k(E,F)$, we obtain a filtration on ${\mathcal {D}}{\mathcal {O}}(E,F)$,

$$\begin{aligned} \Gamma ^\infty (\hom (E,F))={\mathcal {D}}{\mathcal {O}}^0(E,F)\subseteq {\mathcal {D}}{\mathcal {O}}^1(E,F)\subseteq {\mathcal {D}}{\mathcal {O}}^2(E,F)\subseteq \cdots , \end{aligned}$$

which is compatible with composition and transposition. More explicitly, if *G* is another vector bundle over *M*, $A\in {\mathcal {D}}{\mathcal {O}}^k(E,F)$ and $B\in {\mathcal {D}}{\mathcal {O}}^l(F,G)$, then $BA\in {\mathcal {D}}{\mathcal {O}}^{l+k}(E,G)$ and $A^t\in {\mathcal {D}}{\mathcal {O}}^k(F',E')$. Here, $A^t$ denotes the transpose (differential) operator characterized by

7 $$\begin{aligned} \langle \phi ,A\psi \rangle =\langle A^t\phi ,\psi \rangle , \end{aligned}$$

for all $\psi \in \Gamma ^\infty _c(E)$ and $\phi \in \Gamma ^\infty _c(F')$, with respect to the canonical pairings $\Gamma ^\infty _c(E')\times \Gamma ^\infty (E)\rightarrow {\mathbb {C}}$ and $\Gamma ^\infty _c(F')\times \Gamma ^\infty (F)\rightarrow {\mathbb {C}}$. Here we are using the notation $E':=E^*\otimes |\Lambda |_M$ where $E^*$ denotes the dual bundle and $|\Lambda |_M$ denotes the bundle of 1-densities on *M*. Note that $(A^t)^t=A$, up to the canonical isomorphism of vector bundles $E''=E$.

#### Remark 2.1

(*The spaces* $\Gamma ^r(E)$) For $r\in {\mathbb {N}}_0$ we let $\Gamma ^r(E)$ denotes the Heisenberg analogue of the space of *r* times continuously differentiable sections of *E*. More precisely, $\Gamma ^r(E)$ denotes the space of all $\psi \in \Gamma ^{-\infty }(E)$ such that $A\psi \in \Gamma (F)$ for all differential operators $A\in {\mathcal {D}}{\mathcal {O}}^r(E,F)$ of Heisenberg order at most *r* and all vector bundles *F*. Here $\Gamma (F)\subseteq \Gamma ^{-\infty }(F)$ denotes the space of continuous sections equipped with the topology of uniform convergence on compact subsets. We equip $\Gamma ^r(E)$ with the coarsest topology such that the maps $A:\Gamma ^r(E)\rightarrow \Gamma (F)$ are continuous for all $A\in {\mathcal {D}}{\mathcal {O}}^r(E,F)$. If $r-k\ge 0$, then each $A\in {\mathcal {D}}{\mathcal {O}}^k(E,F)$ induces a continuous operator, $A:\Gamma ^r(E)\rightarrow \Gamma ^{r-k}(F)$. Note that we have continuous inclusions $\cdots \subseteq \Gamma ^2(E)\subseteq \Gamma ^1(E)\subseteq \Gamma ^0(E)$ and topological isomorphisms $\Gamma ^0(E)=\Gamma (E)$ as well as $\bigcap _r\Gamma ^r(E)=\Gamma ^\infty (E)$. We will denote the compactly supported analogue by $\Gamma ^r_c(E)$.

#### Remark 2.2

(*Universal differential operators*) Consider a vector bundle *E* over *M* and let $J^kE\rightarrow E$ denote the bundle of Heisenberg *k*-jets of sections of *E*. This is a smooth vector bundle whose fiber over $x\in M$ coincides with the vector space of Heisenberg *k*-jets at *x* of sections of *E*. Recall that two sections $\psi _1,\psi _2\in \Gamma ^\infty (E)$ are said to represent the same Heisenberg *k*-jet at *x* if $A(\psi _2-\psi _1)(x)=0$ for all differential operators $A\in {\mathcal {D}}{\mathcal {O}}^k(E,F)$. Assigning to a section of *E* its Heisenberg *k*-jet, we obtain a differential operator $j^k:\Gamma ^\infty (E)\rightarrow \Gamma ^\infty (J^kE)$. In fact, $j^k\in {\mathcal {D}}{\mathcal {O}}^k(E,J^kE)$, and this differential operator is universal in the following sense: For every $A\in {\mathcal {D}}{\mathcal {O}}^k(E,F)$ there exists a unique smooth vector bundle homomorphism $\alpha :J^kE\rightarrow F$ such that $A=\alpha \circ j^k$. We refer to [56, Section 3.1], [58, Section 1.2.6] or [57] for details.

A differential operator $A\in {\mathcal {D}}{\mathcal {O}}^k(E,F)$ has a *Heisenberg principal cosymbol* at each $x\in M$,

$$\begin{aligned} \sigma _x^k(A)\in {\mathcal {U}}_{-k}({\mathfrak {t}}_xM)\otimes \hom (E_x,F_x), \end{aligned}$$

where ${\mathcal {U}}_{-k}({\mathfrak {t}}_xM)$ denotes the degree $-k$ part of the universal enveloping algebra of the graded Lie algebra ${\mathfrak {t}}_xM=\bigoplus _p{\mathfrak {t}}_x^pM$. More explicitly, ${\mathcal {U}}_{-k}({\mathfrak {t}}_xM)$ can be described as the linear subspace of ${\mathcal {U}}({\mathfrak {t}}_xM)$ spanned by all elements of the form $X_m\cdots X_1$ where $X_i\in {\mathfrak {t}}_x^{p_i}M$ and $-k=p_m+\cdots +p_1$. The Heisenberg principal cosymbol provides a short exact sequence

$$\begin{aligned} 0\rightarrow {\mathcal {D}}{\mathcal {O}}^{k-1}(E,F)\rightarrow {\mathcal {D}}{\mathcal {O}}^k(E,F)\xrightarrow {\sigma ^k}\Gamma ^\infty \bigl ({\mathcal {U}}_{-k}(\mathfrak tM)\otimes \hom (E,F)\bigr )\rightarrow 0 \end{aligned}$$

where ${\mathcal {U}}_{-k}(\mathfrak tM):=\bigsqcup _{x\in M}{\mathcal {U}}_{-k}({\mathfrak {t}}_xM)$ is a smooth vector bundle of finite rank according to the Poincaré–Birkhoff–Witt theorem. Details may be found in [58, Section 1.2.5].

If $A\in {\mathcal {D}}{\mathcal {O}}^k(E,F)$ and $B\in {\mathcal {D}}{\mathcal {O}}^l(F,G)$ where *G* is another vector bundle over *M*, then

8 $$\begin{aligned} \sigma _x^{l+k}(BA)=\sigma _x^l(B)\sigma ^k_x(A) \qquad \text {and}\qquad \sigma _x^k(A^t)=\sigma _x^k(A)^t. \end{aligned}$$

To explain the second equation in (8), we extend $-{{\,\mathrm{id}\,}}:{\mathfrak {t}}_xM\rightarrow {\mathfrak {t}}_xM$ to an anti-automorphism of ${\mathcal {U}}({\mathfrak {t}}_xM)$, ${\mathbf {X}}\mapsto {\mathbf {X}}^t$. Hence, $({\mathbf {X}}^t)^t={\mathbf {X}}$ and $({\mathbf {X}}{\mathbf {Y}})^t={\mathbf {Y}}^t{\mathbf {X}}^t$ for all ${\mathbf {X}},{\mathbf {Y}}\in {\mathcal {U}}({\mathfrak {t}}_xM)$. This *antipode* preserves the grading components ${\mathcal {U}}_{-k}({\mathfrak {t}}_xM)$. We extend this further to a transposition ${\mathcal {U}}({\mathfrak {t}}_xM)\otimes \hom (E_x,F_x)\rightarrow {\mathcal {U}}({\mathfrak {t}}_xM)\otimes \hom (F_x',E_x')$ characterized by $({\mathbf {X}}\otimes \Phi )^t:={\mathbf {X}}^t\otimes \Phi ^t\otimes {{\,\mathrm{id}\,}}_{|\Lambda |_{M,x}}$ for all $\Phi \in \hom (E_x,F_x)$ and ${\mathbf {X}}\in {\mathcal {U}}({\mathfrak {t}}_xM)$ where $\Phi ^t\in \hom (F_x^*,E_x^*)$ denotes the linear map dual to $\Phi$. This is the transposition used in $\sigma _x^k(A)^t$, see (8).

If $\nabla$ is a linear connection on *E* and $X\in \Gamma ^\infty (T^{-k}M)$, then $\nabla _X\in {\mathcal {D}}{\mathcal {O}}^k(E)$ and

9 $$\begin{aligned} \sigma ^k(\nabla _X)=[X]\otimes {{\,\mathrm{id}\,}}_E\in \Gamma ^\infty \bigl ({\mathcal {U}}_{-k}(\mathfrak tM)\otimes {{\,\mathrm{end}\,}}(E)\bigr ) \end{aligned}$$

where [*X*] denotes the section of ${\mathfrak {t}}^{-k}M=T^{-k}M/T^{-k+1}M$ represented by *X*. This property, together with the multiplicativity in (8) and the requirement $\sigma ^0(A)=A$ for all $A\in {\mathcal {D}}{\mathcal {O}}^0(E,F)=\Gamma ^\infty (\hom (E,F))=\Gamma ^\infty ({\mathcal {U}}_0(\mathfrak tM)\otimes \hom (E,F))$, characterizes the Heisenberg principal symbol uniquely.

#### Remark 2.3

(*Formal adjoints*) Suppose $A\in {\mathcal {D}}{\mathcal {O}}^k(E,F)$ and let $A^*$ denote the formal adjoint with respect to $L^2$ inner products associated with a smooth volume density *dx* on *M* and smooth fiber-wise Hermitian inner products $h_E$ and $h_F$ on the vector bundles *E* and *F*, respectively. Hence, $A^*$ is characterized by

10 $$\begin{aligned} \langle \!\langle A^*\phi ,\psi \rangle \!\rangle _{L^2(E)}=\langle \!\langle \phi ,A\psi \rangle \!\rangle _{L^2(F)} \end{aligned}$$

for all $\phi \in \Gamma ^\infty _c(F)$ and $\psi \in \Gamma ^\infty _c(E)$, where

11 $$\begin{aligned} \langle \!\langle \psi _1,\psi _2\rangle \!\rangle _{L^2(E)}=\int _Mh_E(\psi _1(x),\psi _2(x))dx =\langle (h_E\otimes dx)\psi _1,\psi _2\rangle \end{aligned}$$

for $\psi _1,\psi _2\in \Gamma _c^\infty (E)$ and similarly for *F*. Here we consider $h_E\otimes dx:{\bar{E}}\rightarrow E'$ as a vector bundle isomorphism. In terms of the transpose, we have

12 $$\begin{aligned} A^*=(h_E\otimes dx)^{-1}\circ A^t\circ (h_F\otimes dx). \end{aligned}$$

In particular, $A^*\in {\mathcal {D}}{\mathcal {O}}^k(F,E)$ and

13 $$\begin{aligned} \sigma _x^k(A^*)=\sigma ^k_x(A)^*. \end{aligned}$$

The involution ${\mathcal {U}}({\mathfrak {t}}_xM)\otimes \hom (E_x,F_x)\rightarrow {\mathcal {U}}({\mathfrak {t}}_xM)\otimes \hom (F_x,E_x)$ used on the right hand side can be characterized by $({\mathbf {X}}\otimes \Phi )^*={\mathbf {X}}^t\otimes \Phi ^*$ for all $\Phi \in \hom (E_x,F_x)$ and ${\mathbf {X}}\in {\mathcal {U}}({\mathfrak {t}}_xM)$ where $\Phi ^*\in \hom (F_x,E_x)$ denotes the adjoint of $\Phi$ with respect to the inner products $h_{E,x}$ and $h_{F,x}$. Equation (13) follows from (12) and (8).

A graded Lie algebra has a natural group of *dilation automorphisms.* Thus, for $\lambda >0$ we let ${\dot{\delta }}_\lambda \in {{\,\mathrm{Aut}\,}}(\mathfrak tM)$ denote the bundle automorphism given by multiplication with $\lambda ^{-p}$ on the grading component ${\mathfrak {t}}^pM$. For each $x\in M$, this restricts to an automorphism ${\dot{\delta }}_{\lambda ,x}\in {{\,\mathrm{Aut}\,}}({\mathfrak {t}}_xM)$ of the osculating algebra such that $\lim _{\lambda \rightarrow 0}{\dot{\delta }}_{\lambda ,x}=0$. Clearly, ${\dot{\delta }}_{\lambda _1\lambda _2}={\dot{\delta }}_{\lambda _1}{\dot{\delta }}_{\lambda _2}$ for all $\lambda _1,\lambda _2>0$. Extending ${\dot{\delta }}_{\lambda ,x}$ to an automorphism of ${\mathcal {U}}({\mathfrak {t}}_xM)$, we can characterize the grading by

14 $$\begin{aligned} {\mathcal {U}}_{-k}({\mathfrak {t}}_xM)=\bigl \{{\mathbf {X}}\in {\mathcal {U}}({\mathfrak {t}}_xM):{\dot{\delta }}_{\lambda ,x}({\mathbf {X}})=\lambda ^k{\mathbf {X}}\text { for all }\lambda >0\bigr \}. \end{aligned}$$

We let $\mathcal {T}M\rightarrow M$ denote the *bundle of osculating groups.* For each $x\in M$, the fiber ${\mathcal {T}}_xM$ is a simply connected nilpotent Lie group, called the *osculating group at x*, with Lie algebra ${\mathfrak {t}}_xM$. The fiber-wise exponential map, $\exp :\mathfrak tM\rightarrow \mathcal {T}M$, provides an isomorphism of smooth fiber bundles. The Lie algebra automorphisms ${\dot{\delta }}_{\lambda ,x}$ integrate to group automorphisms $\delta _{\lambda ,x}\in {{\,\mathrm{Aut}\,}}({\mathcal {T}}_xM)$ which assemble to a smooth bundle automorphism $\delta _\lambda \in {{\,\mathrm{Aut}\,}}(\mathcal {T}M)$ such that $\exp \circ {\dot{\delta }}_\lambda =\delta _\lambda \circ \exp$. Clearly, $\delta _{\lambda _1\lambda _2}=\delta _{\lambda _1}\delta _{\lambda _2}$, for all $\lambda _1,\lambda _2>0$.

Since the universal enveloping algebra of ${\mathfrak {t}}_xM$ can be identified with the algebra of left invariant differential operators on ${\mathcal {T}}_xM$, and in view of (14), the Heisenberg principal symbol of $A\in {\mathcal {D}}{\mathcal {O}}^k(E,F)$ can equivalently be regarded as a *left invariant differential operator,*

15 $$\begin{aligned} \sigma _x^k(A):C^\infty ({\mathcal {T}}_xM,E_x)\rightarrow C^\infty ({\mathcal {T}}_xM,F_x), \end{aligned}$$

which is *homogeneous* of degree *k*, that is,

16 $$\begin{aligned} \sigma ^k_x(A)\circ l_g^*=l_g^*\circ \sigma ^k_x(A) \qquad \text {and}\qquad \sigma ^k_x(A)\circ \delta _{\lambda ,x}^*=\lambda ^k\cdot \delta _{\lambda ,x}^*\circ \sigma ^k_x(A) \end{aligned}$$

for all $g\in {\mathcal {T}}_xM$ and $\lambda >0$. Here, $l_g^*$ denotes pull back along the left translation, $l_g:{\mathcal {T}}_xM\rightarrow {\mathcal {T}}_xM$, $l_g(h):=gh$, and $\delta _{\lambda ,x}^*$ denotes pull back along the dilation discussed above.

#### Remark 2.4

If the filtration on *M* is trivial, that is to say, if $T^{-1}M=TM$, then the filtration on differential operators is the usual one. In this case ${\mathcal {T}}_xM=T_xM$ is an Abelian Lie group and the principal cosymbol $\sigma ^k_x(A)$ of a differential operator *A* is a translation invariant (constant coefficient) differential operator on $T_xM$.

### Parametrices

As we have seen above, the Heisenberg principal symbols of a differential operator can be described by homogeneous left invariant operators on the osculating Lie groups. This is the primary reason why the osculating groups and their representation theory, and particularly the Rockland condition, become relevant to the analysis of these operators. We shall now briefly recall some facts from representation theory necessary to formulate the Rockland condition for differential operators, see Definition 2.5, and state the corresponding Rockland type theorem, see Theorem 2.6.

Let *G* be a Lie group with Lie algebra ${\mathfrak {g}}$. Suppose $\pi :G\rightarrow U({\mathcal {H}})$ is a *unitary representation* of *G* on a Hilbert space ${\mathcal {H}}$. These representations will always be assumed to be *strongly continuous*, that is, the map $G\rightarrow {\mathcal {H}}$, $g\mapsto \pi (g)v$, is assumed to be continuous for every vector $v\in {\mathcal {H}}$. For unitary representations, this is actually equivalent to *weak continuity* which only asserts that the function $G\rightarrow {\mathbb {C}}$, $g\mapsto \langle \!\langle \pi (g)v,w\rangle \!\rangle _{{\mathcal {H}}}$, is continuous for any two vectors $v,w\in {\mathcal {H}}$, see [49, Theorem 1 in Appendix V]. Rarely will the representations we shall encounter be continuous with respect to the norm topology on $U({\mathcal {H}})$.

Recall that $v\in {\mathcal {H}}$ is called *smooth vector* if the map $G\rightarrow {\mathcal {H}}$, $g\mapsto \pi (g)v$, is (strongly) smooth. According to [49, Theorem 3 in Appendix V] this is equivalent to the weak assumption: the function $G\rightarrow {\mathbb {C}}$, $g\mapsto \langle \!\langle \pi (g)v,w\rangle \!\rangle _{{\mathcal {H}}}$, is smooth for all vectors $w\in {\mathcal {H}}$. We will denote the subspace of smooth vectors by ${\mathcal {H}}_\infty$. This is a dense subspace in ${\mathcal {H}}$ which is invariant under the operators $\pi (g)$ for all $g\in G$, see [49, Theorem 4(1) in Appendix V]. For each $X\in {\mathfrak {g}}$ we may define, see [49, Theorem 4(2) in Appendix V],

$$\begin{aligned} \pi (X):{\mathcal {H}}_\infty \rightarrow {\mathcal {H}}_\infty ,\qquad \pi (X)v:=\tfrac{\partial }{\partial t}\big |_{t=0}\pi (\exp (tX))v, \end{aligned}$$

where $v\in {\mathcal {H}}_\infty$. By unitarity, $\langle \!\langle \pi (X)v,w\rangle \!\rangle _{{\mathcal {H}}}=\langle \!\langle v,\pi (-X)w\rangle \!\rangle _{{\mathcal {H}}}$ for all $v,w\in {\mathcal {H}}_\infty$. Hence, $\pi (X)$ has a densely defined adjoint, $\pi (X)^*=\pi (-X)$, and, in particular, $\pi (X)$ is closeable, see [49, Theorem 4(2) in Appendix V]. Clearly, $\pi ([X,Y])=\pi (X)\pi (Y)-\pi (Y)\pi (X)$ for any two $X,Y\in {\mathfrak {g}}$. Extending the definition of $\pi$ to the universal enveloping algebra of ${\mathfrak {g}}$, we obtain $\pi ({\mathbf {X}}):{\mathcal {H}}_\infty \rightarrow {\mathcal {H}}_\infty$ for ${\mathbf {X}}\in {\mathcal {U}}({\mathfrak {g}})$ such that

17 $$\begin{aligned} \pi ({\mathbf {X}})\pi ({\mathbf {Y}})=\pi ({\mathbf {X}}{\mathbf {Y}}) \end{aligned}$$

for all ${\mathbf {X}},{\mathbf {Y}}\in {\mathcal {U}}({\mathfrak {g}})$. We let ${\mathbf {X}}\mapsto {\mathbf {X}}^t$ denote the antipode of ${\mathcal {U}}({\mathfrak {g}})$ obtained by extending $-{{\,\mathrm{id}\,}}:{\mathfrak {g}}\rightarrow {\mathfrak {g}}$ to the universal enveloping algebra. Hence, $({\mathbf {X}}^t)^t={\mathbf {X}}$ and $({\mathbf {X}}{\mathbf {Y}})^t={\mathbf {Y}}^t{\mathbf {X}}^t$ for all ${\mathbf {X}},{\mathbf {Y}}\in {\mathcal {U}}({\mathfrak {g}})$. For each ${\mathbf {X}}\in {\mathcal {U}}({\mathfrak {g}})$ we thus have

18 $$\begin{aligned} \pi ({\mathbf {X}})^*=\pi ({\mathbf {X}}^t) \end{aligned}$$

as operators on ${\mathcal {H}}_\infty$.

If $E_0$ and $F_0$ are two finite dimensional vector spaces and $a\in {\mathcal {U}}({\mathfrak {g}})\otimes \hom (E_0,F_0)$ we let

$$\begin{aligned} \pi (a):{\mathcal {H}}_\infty \otimes E_0\rightarrow {\mathcal {H}}_\infty \otimes F_0 \end{aligned}$$

denote the linear operator obtained by linearly extending the definition $\pi ({\mathbf {X}}\otimes \Phi ):=\pi ({\mathbf {X}})\otimes \Phi$ for all $\Phi \in \hom (E_0,F_0)$ and ${\mathbf {X}}\in {\mathcal {U}}({\mathfrak {g}})$. Equivalently, using bases of $E_0$ and $F_0$ to identify *a* with a matrix with entries in ${\mathcal {U}}({\mathfrak {g}})$, the operator $\pi (a)$ corresponds to a matrix of the same size whose entries are operators on ${\mathcal {H}}_\infty$ obtained by applying $\pi$ to the corresponding entry of *a*. The multiplicativity in (17) immediately implies

19 $$\begin{aligned} \pi (ba)=\pi (b)\pi (a) \end{aligned}$$

for all $a\in {\mathcal {U}}({\mathfrak {g}})\otimes \hom (E_0,F_0)$ and $b\in {\mathcal {U}}({\mathfrak {g}})\otimes \hom (F_0,G_0)$ where $G_0$ is another finite dimensional vector space. If, moreover, $E_0$ and $F_0$ are equipped with Hermitian inner products, then (18) leads to

20 $$\begin{aligned} \pi (a)^*=\pi (a^*) \end{aligned}$$

as operators ${\mathcal {H}}_\infty \otimes F_0\rightarrow {\mathcal {H}}_\infty \otimes E_0$. Here the adjoint on the left hand side of (20) is with respect to the inner products on ${\mathcal {H}}_\infty \otimes E_0$ and ${\mathcal {H}}_\infty \otimes F_0$ induced by inner products on $E_0$ and $F_0$ and the restriction of the inner product on ${\mathcal {H}}$. On the right hand side of (20), $a^*\in {\mathcal {U}}({\mathfrak {g}})\otimes \hom (F_0,E_0)$ is defined by linear extension of $({\mathbf {X}}\otimes \Phi )^*:={\mathbf {X}}^t\otimes \Phi ^*$ for all ${\mathbf {X}}\in {\mathcal {U}}({\mathfrak {g}})$ and $\Phi \in \hom (E_0,F_0)$ where $\Phi ^*\in \hom (F_0,E_0)$ denotes the adjoint of $\Phi$. Equivalently, using orthogonal bases of $E_0$ and $F_0$ to identify *a* with a matrix with entries in ${\mathcal {U}}({\mathfrak {g}})$, $a^*$ corresponds to the matrix obtained by taking the transpose conjugate of *a* and applying the antipode ${\mathbf {X}}\mapsto {\mathbf {X}}^t$ to each entry.

#### Definition 2.5

(*Rockland condition*) Let *E* and *F* be vector bundles over a filtered manifold *M*. A differential operator $A\in {\mathcal {D}}{\mathcal {O}}^k(E,F)$ of Heisenberg order at most *k* is said to satisfy the *Rockland condition* if $\pi (\sigma _x^k(A)):{\mathcal {H}}_\infty \otimes E_x\rightarrow {\mathcal {H}}_\infty \otimes F_x$ is injective for every point $x\in M$ and every non-trivial irreducible unitary representation $\pi :{\mathcal {T}}_xM\rightarrow U({\mathcal {H}})$ of the osculating group ${\mathcal {T}}_xM$ on a Hilbert space ${\mathcal {H}}$. Here ${\mathcal {H}}_\infty$ denotes the subspace of smooth vectors in ${\mathcal {H}}$.

We let ${\mathcal {O}}(E,F)$ denote the space of operators $\Gamma ^\infty _c(E)\rightarrow \Gamma ^{-\infty }(F)$ corresponding to Schwartz kernels with wave front set contained in the conormal bundle of the diagonal. These are precisely the operators whose kernel is smooth away from the diagonal and which map $\Gamma ^\infty _c(E)$ continuously into $\Gamma ^\infty (F)$. If $A\in {\mathcal {O}}(E,F)$, then $A^t\in {\mathcal {O}}(F',E')$, cf. (7). The transpose permits extending *A* continuously to distributional sections, $A:\Gamma ^{-\infty }_c(E)\rightarrow \Gamma ^{-\infty }(F)$, such that $\langle A^t\phi ,\psi \rangle =\langle \phi ,A\psi \rangle$ for all $\psi \in \Gamma ^{-\infty }_c(E)$ and $\phi \in {\mathcal {D}}(F):=\Gamma ^\infty _c(F')$, and this extension is pseudolocal, i.e., ${{\,\mathrm{sing-supp}\,}}(A\psi )\subseteq {{\,\mathrm{sing-supp}\,}}(\psi )$ for all $\psi \in \Gamma ^{-\infty }_c(E)$. Recall that an operator with Schwartz kernel *k* is called *properly supported* if the two projections $M\times M\rightarrow M$ both restrict to proper maps on the support of *k*. If $B\in {\mathcal {O}}(F,G)$ and at least one of *A* or *B* is properly supported, then $BA\in {\mathcal {O}}(E,G)$ and $(BA)^t=A^tB^t$. We refer to [28, 74] for details.

We have the following vector valued analogue of a result due to Melin [52, Theorem 7.2]:

#### Theorem 2.6

(Left parametrix) Let *E* and *F* be vector bundles over a filtered manifold *M* and suppose $A\in {\mathcal {D}}{\mathcal {O}}^k(E,F)$ is a differential operator of Heisenberg order at most *k* which satisfies the Rockland condition, see Definition 2.5. Then, there exists a properly supported left parametrix $B\in {\mathcal {O}}_\text {prop}(F,E)$ such that $BA-{{\,\mathrm{id}\,}}$ is a smoothing operator.

Melin [52] considers the scalar case and shows that the parametrix may be chosen to be a pseudodifferential operator of Heisenberg order $-k$ in the calculus constructed in said paper. In Sect. 3 we will formulate and prove a generalization of this result for pseudodifferential operators of any order, see Theorem 3.11. This will allow us to refine the subsequent hypoellipticity statements, see Sect. 3.3, and to extend them to the graded setup required for the analysis of (generalized) BGG sequences, see Sect. 5.

#### Remark 2.7

Let us point out that several special cases of this result are well known. To begin with, for trivially filtered manifolds, i.e., $TM=T^{-1}M$, this reduces to the classical, elliptic case. In this situation all irreducible unitary representations of the (abelian) osculating group are one dimensional, and the scalar Rockland condition at $x\in M$ becomes the familiar condition that the principal symbol of the operator is invertible at every $0\ne \xi \in T_x^*M$.

Another well-studied class are the contact and (more generally) Heisenberg manifolds. For Heisenberg manifolds, a pseudodifferential calculus has been developed independently by Beals–Greiner [3] and Taylor [73], see also [59]. Special cases of Theorem 2.6 for Heisenberg manifolds can be found in [3, Theorem 8.4] or [59, Theorem 5.4.1]. These investigations can be traced back to the work of Kohn [50], Boutet de Monvel [25], and Folland–Stein [33] on CR manifolds. For more historical comments, we refer to the introduction of [3].

If the filtration on *M* is locally diffeomorphic to that on a graded nilpotent Lie group, then the scalar version of Theorem 2.6 can be found in [17, Theorem 2.5(d)]. This suffices to study the flat models in parabolic geometry given by the homogeneous spaces *G*/*P*, as well as topologically stable [60] structures like contact and Engel manifolds.

#### Corollary 2.8

(Hypoellipticity) Let *E* and *F* be vector bundles over a filtered manifold *M* and suppose $A\in {\mathcal {D}}{\mathcal {O}}^k(E,F)$ is a differential operator of Heisenberg order at most *k* which satisfies the Rockland condition. Then, *A* is hypoelliptic, that is, if $\psi$ is a compactly supported distributional section of *E* and $A\psi$ is smooth on an open subset *U* of *M*, then $\psi$ was smooth on *U*. If, moreover, *M* is closed, then $\ker (A)$ is a finite dimensional subspace of $\Gamma ^\infty (E)$.

#### Proof

We recall a standard argument. Let $B\in {\mathcal {O}}_\text {prop}(F,E)$ be a left parametrix as in Theorem 2.6. Hence, $BA-{{\,\mathrm{id}\,}}$ is a smoothing operator and $BA\psi -\psi$ is a smooth section of *E*. Moreover, $BA\psi$ is a smooth on *U*, for *B* is pseudolocal. Consequently, $\psi$ is smooth on *U*.

Assume *M* to be closed. By hypoellipticity, $\ker (A)\subseteq \Gamma ^\infty (E)\subseteq L^2(E)$. Since $BA-{{\,\mathrm{id}\,}}$ is a smoothing operator, the identical map on $\ker (A)$ coincides with the restriction of a smoothing operator. The latter induces a compact operator on $L^2(E)$ according to the theorem of Arzelà–Ascoli. Hence, every bounded subset of $\ker (A)$ is precompact in $L^2(E)$. Consequently, $\ker (A)$ has to be finite dimensional. $\square$

#### Corollary 2.9

(Hodge decomposition) Let *E* be a vector bundle over a closed filtered manifold *M*. Suppose $A\in {\mathcal {D}}{\mathcal {O}}^k(E)$ satisfies the Rockland condition and is formally self-adjoint, $A^*=A$, with respect to an $L^2$ inner product of the form (11). Moreover, let *Q* denote the orthogonal projection onto the (finite dimensional) subspace $\ker (A)\subseteq \Gamma ^\infty (E)$. Then, $A+Q$ is invertible with inverse $(A+Q)^{-1}\in {\mathcal {O}}(E)$. Consequently, we have topological isomorphisms and Hodge type decompositions:

$$\begin{aligned} A+Q:\Gamma ^\infty (E)&\xrightarrow \cong \Gamma ^\infty (E),&\Gamma ^\infty (E)&=\ker (A)\oplus A(\Gamma ^\infty (E)),\\ A+Q:\Gamma ^{-\infty }(E)&\xrightarrow \cong \Gamma ^{-\infty }(E),&\Gamma ^{-\infty }(E)&=\ker (A)\oplus A(\Gamma ^{-\infty }(E)). \end{aligned}$$

#### Proof

We recall the classical argument [35] which will be referred to in the proof of Corollary 3.12. According to Theorem 2.6 there exists $B\in {\mathcal {O}}(E)$ such that $BA-{{\,\mathrm{id}\,}}$ is a smoothing operator. Since *A* is formally self-adjoint, $AB^*-{{\,\mathrm{id}\,}}$ is a smoothing operator too. We conclude that *B* and $B^*$ differ by a smoothing operator. Hence, $P^*=P:=\tfrac{1}{2}(B+B^*)\in {\mathcal {O}}(E)$ is a formally self-adjoint parametrix such that $PA-{{\,\mathrm{id}\,}}$ and $AP-{{\,\mathrm{id}\,}}$ are both smoothing operators.

Note that $Q=Q^*$, the orthogonal projection onto $\ker (A)$, is a smoothing operator. In particular, $A+Q$ is hypoelliptic. Moreover, $\ker (A+Q)=0$ in view of $A^*=A$. Since $AP-{{\,\mathrm{id}\,}}$ is a smoothing operator, arguing as in the proof of Corollary 2.8 shows that *P* is hypoelliptic and $\ker (P)$ is a finite dimensional subspace of $\Gamma ^\infty (E)$. Adding the orthogonal projection onto $\ker (P)$ to $P=P^*$, we may furthermore assume $\ker (P)=0$.

Consider $G:=(A+Q)P\in {\mathcal {O}}(E)$. Since $G-{{\,\mathrm{id}\,}}$ is a smoothing operator, it induces a compact operator on every classical Sobolev space $H^s_\text {classical}(E)$. Hence, *G* induces a Fredholm operator with vanishing index on $H^s_\text {classical}(E)$ for all real numbers *s*. By construction, *G* is injective, whence invertible with bounded inverse on $H^s_\text {classical}(E)$. Using the classical Sobolev embedding theorem, we conclude that *G* is invertible on $\Gamma ^\infty (E)$ with continuous inverse, $G^{-1}:\Gamma ^\infty (E)\rightarrow \Gamma ^\infty (E)$. Using $G^*=P(A+Q)$, the same argument shows that $G^*$ is invertible on $\Gamma ^\infty (E)$ with continuous inverse, $(G^*)^{-1}:\Gamma ^\infty (E)\rightarrow \Gamma ^\infty (E)$. Since $(G^*)^{-1}$ is the formal adjoint of $G^{-1}$, we conclude that $G^{-1}$ extends continuously to distributional sections, $G^{-1}:\Gamma ^{-\infty }(E)\rightarrow \Gamma ^{-\infty }(E)$. Thus, according to the Schwartz kernel theorem, $G^{-1}$ is given by a (distributional) kernel we will denote by $G^{-1}$ too. The obvious relation $G^{-1}-{{\,\mathrm{id}\,}}=-(G-{{\,\mathrm{id}\,}})G^{-1}$ implies that $G^{-1}-{{\,\mathrm{id}\,}}$ is a smoothing operator and, consequently, $G^{-1}\in {\mathcal {O}}(E)$. We conclude $(A+Q)^{-1}=PG^{-1}\in {\mathcal {O}}(E)$. The remaining assertions follow at once. $\square$

### Rockland sequences

Let us now generalize the concept of elliptic sequences of differential operators to filtered manifolds. In subsequent sections we will generalize further to the graded setup, see Definition 4.2, and pseudodifferential operators, see Definition 5.6.

#### Definition 2.10

(*Rockland sequences of differential operators*) Let $E_i$ be smooth vector bundles over a filtered manifold *M*. A sequence of differential operators,

21 $$\begin{aligned} \cdots \rightarrow \Gamma ^\infty (E_{i-1})\xrightarrow {A_{i-1}}\Gamma ^\infty (E_i)\xrightarrow {A_i}\Gamma ^\infty (E_{i+1})\rightarrow \cdots , \end{aligned}$$

with $A_i\in {\mathcal {D}}{\mathcal {O}}^{k_i}(E_i,E_{i+1})$, is called *Rockland sequence* if for every $x\in M$ and all non-trivial irreducible unitary representation of the osculating group, $\pi :{\mathcal {T}}_xM\rightarrow U({\mathcal {H}})$, the sequence

22 $$\begin{aligned} \cdots \rightarrow {\mathcal {H}}_\infty \otimes E_{i-1,x}\xrightarrow {\pi (\sigma ^{k_{i-1}}_x(A_{i-1}))} {\mathcal {H}}_\infty \otimes E_{i,x}\xrightarrow {\pi (\sigma ^{k_i}_x(A_i))} {\mathcal {H}}_\infty \otimes E_{i+1,x}\rightarrow \cdots \end{aligned}$$

is weakly exact, i.e., the image of the left arrow is contained and dense in the kernel of the right arrow. Here ${\mathcal {H}}_\infty$ denotes the subspace of smooth vectors in the Hilbert space ${\mathcal {H}}$.

#### Remark 2.11

For every Rockland sequence of differential operators, the sequence (22) is actually exact in the strict (algebraic) sense. This follows from Lemma 2.14 and Remark 3.16.

#### Remark 2.12

If a sequence of differential operators as in (21) is a Rockland sequence, then so are the transposed sequence,

$$\begin{aligned} \cdots \leftarrow \Gamma ^\infty (E_{i-1}')\xleftarrow {A_{i-1}^t}\Gamma ^\infty (E_i')\xleftarrow {A_i^t}\Gamma ^\infty (E_{i+1}')\leftarrow \cdots , \end{aligned}$$

and the sequence of formal adjoints with respect to $L^2$ inner products of the form (11).

#### Remark 2.13

Consider the case $E_i=0$ for all $i\ne 1,2$. In other words, consider a sequence with a single differential operator of Heisenberg order at most $k_1$,

$$\begin{aligned} 0\rightarrow \Gamma ^\infty (E_1)\xrightarrow {A_1}\Gamma ^\infty (E_2)\rightarrow 0. \end{aligned}$$

Such a sequence is hypoelliptic in the sense of Definition 2.10 iff, for all points $x\in M$,

$$\begin{aligned} \pi (\sigma ^{k_1}_x(A_1)):{\mathcal {H}}_\infty \otimes E_{1,x}\rightarrow {\mathcal {H}}_\infty \otimes E_{2,x} \end{aligned}$$

is injective with dense image for all non-trivial irreducible unitary representations $\pi :{\mathcal {T}}_xM\rightarrow U({\mathcal {H}})$. Equivalently, $A_1$ and $A_1^t$ both satisfy the Rockland condition, see Definition 2.5.

Consider a Rockland sequence of differential operators as in (21). To study this sequence we shall introduce certain additional structures and operators in analogy with the standard elliptic sequences. Fix a smooth volume density *dx* on *M*, let $h_i$ be smooth fiber-wise Hermitian inner products on $E_i$, and consider the associated $L^2$ inner products on $\Gamma ^\infty _c(E_i)$,

23 $$\begin{aligned} \langle \!\langle \psi _1,\psi _2\rangle \!\rangle _{L^2(E_i)} =\int _Mh_i(\psi _1(x),\psi _2(x))\hbox {d}x =\langle (h_i\otimes \hbox {d}x)\psi _1,\psi _2\rangle \end{aligned}$$

where $\psi _1,\psi _2\in \Gamma _c^\infty (E_i)$. Moreover, let $A_i^*\in {\mathcal {D}}{\mathcal {O}}^{k_i}(E_{i+1},E_i)$ denote the corresponding formal adjoint, that is, $\langle \!\langle A_i^*\phi ,\psi \rangle \!\rangle _{L^2(E_i)}=\langle \!\langle \phi ,A_i\psi \rangle \!\rangle _{L^2(E_{i+1})}$ for $\psi \in \Gamma ^\infty _c(E_i)$ and $\phi \in \Gamma _c^\infty (E_{i+1})$.

Assume $k_i\ge 1$, choose positive integers $a_i$ such that

24 $$\begin{aligned} k_{i-1}a_{i-1}=k_ia_i=:\kappa , \end{aligned}$$

and consider the differential operator $\Delta _i\in {\mathcal {D}}{\mathcal {O}}^{2\kappa }(E_i)$,

25 $$\begin{aligned} \Delta _i:=(A_{i-1}A_{i-1}^*)^{a_{i-1}}+(A_i^*A_i)^{a_i}. \end{aligned}$$

We will refer to these operators as *Rumin–Seshadri operators* since they generalize the fourth order Laplacians associated with the Rumin complex in [70, Section 2.3].

#### Lemma 2.14

The Rumin–Seshadri operators $\Delta _i$ satisfy the Rockland condition.

#### Proof

Consider $x\in M$ and let $\pi :{\mathcal {T}}_xM\rightarrow U({\mathcal {H}})$ be a non-trivial irreducible unitary representation. We equip ${\mathcal {H}}_\infty \otimes E_{i,x}$ with the Hermitian inner product provided by the restriction of the scalar product of the Hilbert space ${\mathcal {H}}$ and the inner product $h_{i,x}$ on $E_{i,x}$. Using (8), (13), (19) and (20), we obtain:

$$\begin{aligned} \pi (\sigma ^\kappa _x(\Delta _i))) =(B_{i-1}B_{i-1}^*)^{a_{i-1}}+(B_i^*B_i)^{a_i} \end{aligned}$$

where we abbreviate $B_i:=\pi (\sigma _x^{k_i}(A_i)):{\mathcal {H}}_\infty \otimes E_{i,x}\rightarrow {\mathcal {H}}_\infty \otimes E_{i+1,x}$ and we consider $B_i^*=\pi (\sigma _x^{k_i}(A_i^*)):{\mathcal {H}}_\infty \otimes E_{i+1,x}\rightarrow {\mathcal {H}}_\infty \otimes E_{i,x}$. Due to positivity,

$$\begin{aligned} \ker (\pi (\sigma _x^{2\kappa }(\Delta _i)))&=\ker \bigl ((B_{i-1}B_{i-1}^*)^{a_{i-1}}\bigr ) \cap \ker \bigl ((B_i^*B_i)^{a_i}\bigr ), \\ \ker \bigl ((B_{i-1}B_{i-1}^*)^{a_{i-1}}\bigr )&=\ker (B_{i-1}^*)\text {, and} \\ \ker \bigl ((B_i^*B_i)^{a_i}\bigr )&=\ker (B_i). \end{aligned}$$

Since $A_i$ is a Rockland sequence, we also have $\ker (B_i)\subseteq \overline{{{\,\mathrm{img}\,}}(B_{i-1})}\perp \ker (B_{i-1}^*)$ and thus $\ker (B_i)\cap \ker (B_{i-1}^*)=0$. Combining this with the preceding equalities, we obtain $\ker (\pi (\sigma _x^{2\kappa }(\Delta _i)))=0$, i.e., $\Delta _i$ satisfies the Rockland condition. $\square$

Combining Corollary 2.8 and Lemma 2.14 we see that each Rumin–Seshadri operator is hypoelliptic. For Rockland sequences this immediately implies:

#### Corollary 2.15

The differential operator $(A_{i-1}^*,A_i):\Gamma ^\infty (E_i)\rightarrow \Gamma ^\infty (E_{i-1}\oplus E_{i+1})$ is hypoelliptic, that is, if $\psi$ is a distributional section of $E_i$ such that $A_{i-1}^*\psi$ and $A_i\psi$ are both smooth on an open subset *U* of *M*, then $\psi$ was smooth on *U*. Moreover,

26 $$\begin{aligned} \ker (\Delta _i|_{\Gamma ^{-\infty }_c(E_i)})= & {} \ker (A_{i-1}^*|_{\Gamma ^{-\infty }_c(E_i)})\cap \ker (A_i|_{\Gamma ^{-\infty }_c(E_i)}) \nonumber \\= & {} \ker (A_{i-1}^*|_{\Gamma ^\infty _c(E_i)})\cap \ker (A_i|_{\Gamma ^\infty _c(E_i)}). \end{aligned}$$

Over closed manifolds Corollary 2.9 applies to $\Delta _i=\Delta _i^*$; hence, $\ker (\Delta _i)$ is a finite dimensional subspace of $\Gamma ^\infty (E_i)$, and we get Hodge type decompositions as in Corollary 2.9 for the Rumin–Seshadri operators. For Rockland complexes over closed manifolds, this implies:

#### Corollary 2.16

If *M* is closed and $A_iA_{i-1}=0$, then we have Hodge type decompositions

$$\begin{aligned} \Gamma ^\infty (E_i)&=A_{i-1}(\Gamma ^\infty (E_{i-1}))\oplus \ker (\Delta _i)\oplus A_i^*(\Gamma ^\infty (E_{i+1})), \\ \Gamma ^{-\infty }(E_i)&=A_{i-1}(\Gamma ^{-\infty }(E_{i-1}))\oplus \ker (\Delta _i)\oplus A_i^*(\Gamma ^{-\infty }(E_{i+1})), \end{aligned}$$

and

$$\begin{aligned} \ker (A_i|_{\Gamma ^\infty (E_i)})&=A_{i-1}(\Gamma ^\infty (E_{i-1}))\oplus \ker (\Delta _i), \\ \ker (A_i|_{\Gamma ^{-\infty }(E_i)})&=A_{i-1}(\Gamma ^{-\infty }(E_{i-1}))\oplus \ker (\Delta _i). \end{aligned}$$

In particular, every cohomology class admits a unique harmonic representative:

$$\begin{aligned} \frac{\ker (A_i|_{\Gamma ^{-\infty }(E_i)})}{{{\,\mathrm{img}\,}}(A_{i-1}|_{\Gamma ^{-\infty }(E_{i-1})})} =\frac{\ker (A_i|_{\Gamma ^\infty (E_i)})}{{{\,\mathrm{img}\,}}(A_{i-1}|_{\Gamma ^\infty (E_{i-1})})} =\ker (\Delta _i) =\ker (A_{i-1}^*)\cap \ker (A_i). \end{aligned}$$

In the subsequent section, these regularity statements will be refined by maximal hypoelliptic estimates. We postpone these more elaborate results because their formulation requires a pseudodifferential calculus for filtered manifolds.

## A Rockland theorem for the Heisenberg calculus

The aim of this section is to prove Theorem 2.6 which lies at the core of the hypoellipticity results discussed in Sects. 2.2 and 2.3. Combining the Heisenberg calculus [52, 79] with harmonic analysis due to Christ et al. [17], and using arguments of Ponge [59], we obtain a more general Rockland type theorem for pseudodifferential operators on filtered manifolds, see Theorem 3.11. In Sect. 3.3 we use this to introduce a Heisenberg Sobolev scale, see Proposition 3.17, and improve upon Corollaries 2.15 and 2.16 by establishing maximal hypoelliptic estimates, see Corollaries 3.24 and 3.25.

### The Heisenberg pseudodifferential calculus

The pseudodifferential calculus on a filtered manifold can be approached via the Heisenberg tangent groupoid [15, 41, 54, 78]. A key feature of the Heisenberg tangent groupoid is an action of ${\mathbb {R}}_+$, the so-called zoom action. The kernels in the Heisenberg calculus can be characterized as the Schwartz kernels that extend across the Heisenberg tangent groupoid in an essentially homogeneous fashion with respect to the zoom action. As already mentioned, this is inspired by a characterization of classical pseudodifferential operators due to Debord and Skandalis [27]. Our first task is to describe the cosymbol space of the Heisenberg calculus and relate it to the harmonic analysis. We will outline the results needed from the Heisenberg calculus and refer the reader to [79] for details. A more self-contained exposition of this part can be found in [22, Section 3].

For two vector bundles *E* and *F* over a filtered manifold *M*, and any complex number *s*, we let $\Psi ^s(E,F)$ denote the class of *pseudodifferential operators* of Heisenberg order at most *s*, mapping sections of *E* to sections of *F*, cf. [79, Definition 19]. There is a *principal cosymbol map*

$$\begin{aligned} \sigma ^s:\Psi ^s(E,F)\rightarrow \Sigma ^s(E,F), \end{aligned}$$

cf. [79, Definition 35], where $\Sigma ^s(E,F)$ denotes the space *principal cosymbols of order* *s*.

To describe the space of cosymbols, we let $\Omega _\pi$ denote the (trivializable) line bundle over $\mathcal {T}M$ obtained by applying the representation $|\det |^{-1}$ of the general linear group to the frame bundle of the vertical bundle $\ker (T\pi )$ of the submersion $\pi :\mathcal {T}M\rightarrow M$. Hence, the restriction of $\Omega _\pi$ to the fiber ${\mathcal {T}}_xM$ identifies canonically with the bundle of volume densities on ${\mathcal {T}}_xM$, that is, $\Omega _\pi |_{{\mathcal {T}}_xM}=|\Lambda |_{{\mathcal {T}}_xM}$.

We let ${\mathcal {K}}(\mathcal {T}M;E,F)$ denote the space of all distributions $k\in \Gamma ^{-\infty }(\hom (\pi ^*E,\pi ^*F)\otimes \Omega _\pi )$ whose wave front set is contained in the conormal of the identical section $M\subseteq \mathcal {T}M$. In particular, these *k* are assumed to be smooth on $\mathcal {T}M\setminus M$. Equivalently, these can be characterized as families of regular distributional volume densities on the fibers ${\mathcal {T}}_xM$ with values in $\hom (E_x,F_x)$, smoothly parametrized by $x\in M$. We also introduce the notation ${\mathcal {K}}^\infty (\mathcal {T}M;E,F):=\Gamma ^\infty (\hom (\pi ^*E,\pi ^*F)\otimes \Omega _\pi )$ for the subspace of smooth cosymbols. A cosymbol *k* is called *properly supported* if $\pi$ restricts to proper map on the support of *k*.

The regular representation of $k\in {\mathcal {K}}(\mathcal {T}M;E,F)$ at $x\in M$ provides a right invariant operator $C^\infty _c({\mathcal {T}}_xM,E_x)\rightarrow C^\infty ({\mathcal {T}}_xM,F_x)$ on the group ${\mathcal {T}}_xM$ with matrix valued convolution kernel $k^x\in \Gamma ^{-\infty }(|\Lambda |_{{\mathcal {T}}_xM})\otimes \hom (E_x,F_x)$. The kernels in ${\mathcal {K}}(\mathcal {T}M;E,F)$ which are supported on the space of units $M\subseteq \mathcal {T}M$ correspond precisely to differential operators $\Gamma ^\infty (\pi ^*E)\rightarrow \Gamma ^\infty (\pi ^*F)$ which are vertical, i.e., commute with functions in the image of the homomorphism $\pi ^*:C^\infty (M)\rightarrow C^\infty (\mathcal {T}M)$, and restrict to right invariant operators on each fiber ${\mathcal {T}}_xM$.

We denote the space of *complete cosymbols* by

$$\begin{aligned} \Sigma (E,F):=\frac{{\mathcal {K}}_\text {prop}(\mathcal {T}M;E,F)}{{\mathcal {K}}_\text {prop}^\infty (\mathcal {T}M;E,F)} =\frac{{\mathcal {K}}(\mathcal {T}M;E,F)}{{\mathcal {K}}^\infty (\mathcal {T}M;E,F)}, \end{aligned}$$

where the subscript indicates properly supported kernels. The fiber-wise convolution product and inversion induce an associative multiplication and a compatible transposition,

$$\begin{aligned} \Sigma (F,G)\times \Sigma (E,F)\xrightarrow *\Sigma (E,G),\qquad \Sigma (E,F)\xrightarrow {t}\Sigma (F',E'). \end{aligned}$$

More explicitly, we have $(l*k)^t=k^t*l^t$ and $(k^t)^t=k$, for $k\in \Sigma (E,F)$ and $l\in \Sigma (F,G)$.

The scaling automorphism $\delta _\lambda$ acts on $\Sigma (E,F)$ in a way compatible with multiplication and transposition. A cosymbol $k\in {\mathcal {K}}(\mathcal {T}M;E,F)$ is called *essentially homogeneous of order* *s* if $(\delta _\lambda )_*k-\lambda ^s k\in {\mathcal {K}}^\infty (\mathcal {T}M;E,F)$, for $\lambda >0$. The space of *principal cosymbols of order* *s* is

$$\begin{aligned} \Sigma ^s(E,F) :=\left\{ k\in \frac{{\mathcal {K}}(\mathcal {T}M;E,F)}{{\mathcal {K}}^\infty (\mathcal {T}M;E,F)}:(\delta _\lambda )_*k=\lambda ^sk\text { for all }\lambda >0\right\} , \end{aligned}$$

cf. [79, Definition 34]. Convolution and transposition are compatible with homogeneity, i.e.,

$$\begin{aligned} \Sigma ^{s_2}(F,G)\times \Sigma ^{s_1}(E,F)\xrightarrow *\Sigma ^{s_1+s_2}(E,G),\qquad \Sigma ^s(E,F)\xrightarrow {t}\Sigma ^s(F',E'). \end{aligned}$$

For $k\in {\mathbb {N}}_0$ there is a canonical inclusion,

27 $$\begin{aligned} \Gamma ^\infty ({\mathcal {U}}_{-k}(\mathfrak tM)\otimes \hom (E,F))\subseteq \Sigma ^k(E,F) \end{aligned}$$

provided by regarding both sides as right invariant vertical operators on ${\mathcal {T}}^\text {op}M$.^1^

The following basic properties of the Heisenberg calculus have been established in [79] for scalar valued operators and integral *s*. It is straight forward to extend this to the slightly more general setup we are considering here, see also [52, 53]. Hence, we have:

#### Proposition 3.1

Let *E*, *F* and *G* be vector bundles over a filtered manifold *M* and let *s* be any complex number. Then, the following hold true:

- We have $\Psi ^s(E,F)\subseteq {\mathcal {O}}(E,F)$, the operators with conormal kernels.
- We have $\Psi ^{s-1}(E,F)\subseteq \Psi ^s(E,F)$, and the following sequence is exact:
  $$\begin{aligned} 0\rightarrow \Psi ^{s-1}(E,F)\rightarrow \Psi ^s(E,F)\xrightarrow {\sigma ^s}\Sigma ^s(E,F)\rightarrow 0 \end{aligned}$$
- $\bigcap _{k\in {\mathbb {N}}}\Psi ^{s-k}(E,F)={\mathcal {O}}^{-\infty }(E,F)$, the smoothing operators.
- If $A\in \Psi ^{s_1}(E,F)$, $B\in \Psi ^{s_2}(F,G)$, and at least one of the two is properly supported, then $BA\in \Psi ^{s_2+s_1}(E,G)$ and $\sigma ^{s_2+s_1}(BA)=\sigma ^{s_2}(B)\sigma ^{s_1}(A)$.
- If $A\in \Psi ^s(E,F)$, then $A^t\in \Psi ^s(F',E')$ and $\sigma ^s(A^t)=\sigma ^s(A)^t$.
- ${\mathcal {D}}{\mathcal {O}}^k(E,F)={\mathcal {D}}{\mathcal {O}}(E,F)\cap \Psi ^k(E,F)$ for all $k\in {\mathbb {N}}_0$, and the principal symbol considered here extends the one for differential operators via the canonical inclusion (27).
- Let $A\in \Psi ^s(E,F)$ and assume that there exists $b\in \Sigma ^{-s}(F,E)$ such that $b\,\sigma ^s(A)=1$. Then, there exists a left parametrix $B\in \Psi ^{-s}_\text {prop}(F,E)$ such that $\sigma ^{-s}(B)=b$ and $BA-{{\,\mathrm{id}\,}}$ is a smoothing operator. An analogous statement involving right parametrices holds true.

Strictly speaking, the statement about the transposed in item (e) above has not been addressed in [79]. However, given the characterization of the calculus in terms of the tangent groupoid, this is a trivial consequence, see [22, Proposition 3.4(e)]. An equivalent statement for formal adjoints can be found in [52, Theorem 5.10].

#### Remark 3.2

For Heisenberg manifolds, a statement similar to Proposition 3.1(g) can be found in [59, Proposition 3.3.1].

#### Remark 3.3

(*Formal adjoints*) If $A\in \Psi ^s(E,F)$ and $A^*$ denotes the formal adjoint with respect to inner products of the form (11), then $A^*\in \Psi ^{{\bar{s}}}(F,E)$ and $\sigma ^s(A^*)=\sigma ^{{\bar{s}}}(A)^*$. Indeed, in view of (12) this follows immediately from the assertions (d), (e), and (f) of Proposition 3.1. Here the adjoint of the cosymbol, $\sigma ^s(A)^*$, is understood as follows: If $k\in {\mathcal {K}}(\mathcal {T}M;E,F)$, then $k^*\in {\mathcal {K}}(\mathcal {T}M;F,E)$ is defined by $k^*(g)=k(g^{-1})^*$ where $g \in {\mathcal {T}}_xM$, and the right hand side denotes the adjoint of $k(g^{-1})$ with respect to the inner products $h_{E,x}$ and $h_{F,x}$ on $E_x$ and $F_x$, respectively. Since this star preserves the subspace ${\mathcal {K}}^\infty (\mathcal {T}M;E,F)$ and commutes with $\delta _\lambda$, it induces an involution $\Sigma ^{{\bar{s}}}(F,E)\xrightarrow {*}\Sigma ^s(E,F)$, for each complex *s*. For $s\in {\mathbb {N}}_0$ this extends the involution in Remark 2.3 via the inclusion (27).

#### Remark 3.4

(*Asymptotic expansion in exponential coordinates*) Let us use exponential coordinates as in [79] to identify an open neighborhood *U* of the zero section in $\mathcal {T}M$ with an open neighborhood *V* of the diagonal in $M\times M$,

$$\begin{aligned} \mathcal {T}M\supseteq U\xrightarrow \varphi V\subseteq M\times M. \end{aligned}$$

This diffeomorphism is obtained by restricting the composition

$$\begin{aligned} \mathcal {T}M\xleftarrow {\exp }\mathfrak tM\xrightarrow {-S}TM\supseteq U'\xrightarrow {(p,\exp ^\nabla )}M\times M. \end{aligned}$$

Here $\exp$ denotes the fiber-wise exponential map; *S* is a splitting of the filtration; $\exp ^\nabla$ denotes the exponential map associated with a linear connection on the tangent bundle which preserves the grading $TM=\bigoplus _pS({\mathfrak {t}}^pM)$; $U'$ is an open neighborhood of the zero section in *TM* on which $\exp ^\nabla$ is defined and a diffeomorphism onto its image; and $p:TM\rightarrow M$ denotes the canonical projection, cf. [79, Section 3.2]. Let $\pi :\mathcal {T}M\rightarrow M$ denote the canonical projection and, after possibly shrinking *U*, fix an isomorphism of vector bundles ${\tilde{\varphi }}$ over $\varphi$,which restricts to the tautological identification over the zero section/diagonal. For every Schwartz kernel $k\in \Gamma ^{-\infty }(F\boxtimes E')$ we obtain ${\tilde{\varphi }}^*(k|_V)\in \Gamma ^{-\infty }((\pi ^*\hom (E,F)\otimes \Omega _\pi )|_U)$. If *k* is the kernel of an operator $A\in \Psi ^s(E,F)$, then we have an asymptotic expansion of the form

28 $$\begin{aligned} {\tilde{\varphi }}^*(k|_V)\sim k_0+k_1+k_2+\cdots , \end{aligned}$$

where $k_j\in \Sigma ^{s-j}(E,F)$ and $\sigma ^s(A)=k_0$. More precisely, for every $r\in {\mathbb {N}}$ there exists $N\in {\mathbb {N}}$ such that ${\tilde{\varphi }}^*(k|_V)-\sum _{j=0}^Nk_j$ is of class $C^r$ on *U*. Conversely, if a Schwartz kernel *k* is smooth away from the diagonal and admits an asymptotic expansion of the form (28), then the corresponding operator is in $\Psi ^s(E,F)$, see [79, Theorem 59]. The calculus is asymptotically complete in the sense that any sequence $k_j$ can be realized by an operator in $\Psi ^s(E,F)$.

#### Remark 3.5

In the flat case, that is to say, if the filtration on *M* is locally diffeomorphic to the left invariant filtration on a graded nilpotent Lie group, the calculus described above coincides with the calculus of Christ et al. [17]. On Heisenberg manifolds it specializes to the classical Heisenberg calculus, see [3, 59, 73], which builds upon work of Boutet de Monvel [25], Folland–Stein [33] and Dynin [29, 30], see also [26, 31, 38, 47, 63]. The equivalence with the Heisenberg calculus follows from [3, Theorems 15.39 and 15.49], see also [59, Proposition 3.1.15], for the exponential coordinates used in Remark 3.4 are clearly privileged coordinates in the sense of [59, Definition 2.1.10]. For trivially filtered manifolds, that is $TM=T^{-1}M$, we recover classical pseudodifferential operators, see [79, Section 11] and [27]. Since the coordinates used by Melin, obtained by integrating [52, Proposition 2.9] connection maps [52, Definition 2.7], include the exponential coordinates used in Remark 3.4, his calculus should coincide with the one sketched above.

Let us now link the principal cosymbols considered above with the principal cosymbols used by Christ et al. [17]. For every complex number *s*, put

$$\begin{aligned} {\mathcal {P}}^s(\mathcal {T}M;E,F):=\bigl \{k\in {\mathcal {K}}^\infty (\mathcal {T}M;E,F): (\delta _\lambda )_*k=\lambda ^sk\text { for all }\lambda >0\bigr \}. \end{aligned}$$

Clearly, ${\mathcal {P}}^s(\mathcal {T}M;E,F)=0$ if $-s-n\notin {\mathbb {N}}_0$ where

29 $$\begin{aligned} n:=-\sum _pp\cdot {{\,\mathrm{rank}\,}}({\mathfrak {t}}^pM)=-\sum _pp\cdot {{\,\mathrm{rank}\,}}(T^pM/T^{p+1}M) \end{aligned}$$

denotes the *homogeneous dimension* of *M*. For $-s-n\in {\mathbb {N}}_0$, by Taylor’s theorem, this is the space of smooth kernels which restrict to (matrices of) polynomial volume densities of homogeneous degree *s* on each fiber ${\mathcal {T}}_xM$, using the exponential map to canonically identify ${\mathcal {T}}_xM$ with the graded vector space ${\mathfrak {t}}_xM$. Equivalently, these can be characterized as smooth families of polynomial volume densities of homogeneous degree *s* on the fibers ${\mathcal {T}}_xM={\mathfrak {t}}_xM$, smoothly parametrized by $x\in M$. We have the following classical fact, cf. [22, Lemma 3.8]:

#### Lemma 3.6

The identical map on ${\mathcal {K}}(\mathcal {T}M;E,F)$ induces a canonical identification

$$\begin{aligned} \Sigma ^s(E,F)=\left\{ k\in \frac{{\mathcal {K}}(\mathcal {T}M;E,F)}{{\mathcal {P}}^s(\mathcal {T}M;E,F)}: (\delta _\lambda )_*k=\lambda ^sk\text { for all }\lambda >0\right\} . \end{aligned}$$

Moreover, with respect to a homogeneous norm $|-|$ on $\mathfrak tM$, and using the fiber-wise exponential map, $\exp :\mathfrak tM\rightarrow \mathcal {T}M$, every kernel $k\in {\mathcal {K}}(\mathcal {T}M;E,F)$ which is essentially homogeneous of order *s* can be written in the form

30 $$\begin{aligned} k=k_\infty +k_s+p_s\log |\exp ^{-1}(-)| \end{aligned}$$

where $k_\infty \in {\mathcal {K}}^\infty (\mathcal {T}M;E,F)$, $k_s\in {\mathcal {K}}(\mathcal {T}M;E,F)$ homogeneous of order *s*, that is, $(\delta _\lambda )_*k_s=\lambda ^sk_s$ for all $\lambda >0$, and $p_s\in {\mathcal {P}}^s(\mathcal {T}M;E,F)$. If $-s-n\notin {\mathbb {N}}_0$, then $p_s=0$ and the decomposition in (30) is unique. If $-s-n\in {\mathbb {N}}_0$, then the decomposition in (30) is unique up to adding a kernel in ${\mathcal {P}}^s(\mathcal {T}M;E,F)$ to $k_s$ and subtracting it from $k_\infty$ in turn.

#### Proposition 3.7

Let *E* and *F* be vector bundles over a filtered manifold *M* of homogeneous dimension *n*, see (29). Consider $A\in \Psi ^s(E,F)$ where *s* is some complex number, and let $k\in \Gamma ^{-\infty }(F\boxtimes E')$ denote the corresponding Schwartz kernel. Then, the following hold true:

- If $\Re s\le 0$, then *A* induces a continuous operator $A:L^2_c(E)\rightarrow L^2_\text {loc}(F)$.
- If $\Re s<0$, then *A* induces a compact operator $A:L^2_c(E)\rightarrow L^2_\text {loc}(F)$.
- If $\Re s<-n/2$, then *A* induces a continuous operator $A:L^2_c(E)\rightarrow \Gamma (F)$, cf. Remark 2.1.
- If $\Re s<-n$, then the kernel *k* is continuous. If, moreover, *M* is closed and $E=F$, then $A:L^2(E)\rightarrow L^2(E)$ is trace class and
  $$\begin{aligned} {{\,\mathrm{tr}\,}}_{L^2(E)}(A)=\int _M{{\,\mathrm{tr}\,}}_E(\iota ^*k)=\int _{x\in M}{{\,\mathrm{tr}\,}}_{E_x}(k(x,x)) \end{aligned}$$
  where $\iota ^*k\in \Gamma ^\infty ({{\,\mathrm{end}\,}}(E)\otimes |\Lambda |_M)$ denotes the restriction of the kernel to the diagonal.

#### Proof

To show (a), suppose $\Re s\le 0$. The symbol estimate in [79, Corollary 45] implies that the full symbol of *A*, i.e., the fiber-wise Fourier transform of the full cosymbol, with respect to exponential coordinates as in Remark 3.4, is in the standard class ${\mathcal {S}}^{s/m}_{1/m,0}$ where *m* is such that $\mathfrak tM=\bigoplus _{j=1}^m{\mathfrak {t}}^{-j}M$, cf. [3, Proposition 10.22]. This symbol class is not invariant under general coordinate change. Nevertheless, boundedness on $L^2$ is a well-known consequence, see, for instance, [72, Theorem 6.1].

To show (b) suppose $\Re s<0$. Using Remark 3.4 and Lemma 3.6 we see that the kernel provides a family $k(x,-)\in L^1_\text {loc}(F_x\otimes E')$, smoothly parametrized by $x\in M$; and the same holds true for the transposed kernel, see Proposition 3.1(e). In particular, given two compact subsets *K* and *L* of *M*, there exists a constant $C\ge 0$ such that $\sup _{x\in L}\int _K|k(x,y)|dy\le C$ and $\sup _{y\in K}\int _L|k(x,y)|dx\le C$. Hence, according to Schur’s lemma, see [72, Lemma 9.1] or [33, Lemma 15.2] for instance, the operator norm of the composition

31 $$\begin{aligned} L^2_K(E)\subseteq L^2_c(E)\xrightarrow {A}L^2_\text {loc}(F)\rightarrow L^2_L(F) \end{aligned}$$

is bounded by *C*, i.e., $\Vert A\psi \Vert _{L^2_L(F)}\le C\Vert \psi \Vert _{L^2_K(E)}$ for all $\psi \in L^2_K(E)$. Writing $k=\chi k+(1-\chi )k$, where $\chi \in C^\infty (M\times M,[0,1])$ and $\chi \equiv 1$ in a neighborhood of the diagonal, we obtain a decomposition $A=A'+R$ where *R* is a smoothing operator with kernel $(1-\chi )k$ and $A'\in \Psi ^s(E,F)$ has kernel $\chi k$. Given $\varepsilon >0$, we may choose $\chi$ such that $\sup _{x\in L}\int _K|\chi k(x,y)|dy\le \varepsilon$ and $\sup _{y\in K}\int _L|\chi k(x,y)|dx\le \varepsilon$ and, consequently, $\Vert A'\psi \Vert _{L^2_L(F)}\le \varepsilon \Vert \psi \Vert _{L^2_K(E)}$. We conclude that the composition in (31) can be approximated by smoothing operators. Since the latter are compact, we conclude that the composition in (31) is compact too.

To show (c) we suppose $\Re s<-n/2$. Using Remark 3.4 and Lemma 3.6 we see that the kernel provides a smooth family $k(x,-)\in L^2_\text {loc}(F_x\otimes E')$, parametrized by $x\in M$. In particular, given two compact subsets *K* and *L* of *M*, there exists a constant $C\ge 0$ such that $\sup _{x\in L}\int _K|k(x,y)|^2dy\le C^2$. Using the Cauchy–Schwarz inequality, we obtain $\sup _{x\in L}|(A\psi )(x)|\le C\Vert \psi \Vert _{L^2_K(E)}$ for all $\psi \in L^2_K(E)$. Hence, *A* maps $L^2_c(E)$ continuously into $\Gamma (F)$.

To show (d), we suppose $\Re s<-n$. Using Remark 3.4 and Lemma 3.6 we see that the kernel provides a family $k(x,-)\in \Gamma (F_x\otimes E')$, smoothly parametrized by $x\in M$. Clearly, this implies that *k* is continuous. The remaining assertions are now obvious. $\square$

For Melin’s calculus, Proposition 3.7(a) can be found in [52, Corollary 6.14].

### Parametrices and Rockland condition

In this section, we establish a Rockland type result characterizing (left) invertible cosymbols and the existence of (left) parametrices in terms of irreducible unitary representations of the osculating groups, see Theorem 3.11. As an application, we construct operators of arbitrary (complex) order which are invertible in the Heisenberg calculus, up to smoothing operators. The latter play a crucial role in the construction of the Heisenberg Sobolev scale.

For every $x\in M$ we let

$$\begin{aligned} \Sigma _x^s(E,F):= \left\{ k\in \frac{{\mathcal {K}}({\mathcal {T}}_xM;E_x,F_x)}{{\mathcal {K}}^\infty ({\mathcal {T}}_xM;E_x,F_x)}: (\delta _\lambda )_*k=\lambda ^sk\text { for all }\lambda >0\right\} \end{aligned}$$

denote the space principal cosymbols of order *s* at *x*. Restriction provides a linear map ${{\,\mathrm{ev}\,}}_x:\Sigma ^s(E,F)\rightarrow \Sigma ^s_x(E,F)$ which is compatible with convolution and transposition. Composing this with the principal symbol map, we obtain

$$\begin{aligned} \sigma ^s_x:\Psi ^s(E,F)\rightarrow \Sigma ^s_x(E,F). \end{aligned}$$

We will refer to $\sigma ^s_x(A)$ as the principal cosymbol of $A\in \Psi ^s(E,F)$ at *x*.

Following [17] and [59, Section 3.3.2], we will now formulate a (matrix) Rockland type condition for cosymbols in $\Sigma _x^s(E,F)$. Let ${\mathcal {P}}({\mathcal {T}}_xM)=\bigoplus _{j=0}^\infty {\mathcal {P}}^{-n-j}({\mathcal {T}}_xM)$ denote the space of polynomial volume densities on ${\mathcal {T}}_xM$, where *n* denotes the homogeneous dimension of *M*, see (29). Recall that ${\mathcal {P}}({\mathcal {T}}_xM)$ is invariant under translation and inversion. For a finite dimensional vector space $E_0$, we let ${\mathcal {S}}_0({\mathcal {T}}_xM;E_0)$ denote the subspace of all *f* in the $E_0$-valued Schwartz space ${\mathcal {S}}({\mathcal {T}}_xM;E_0)$ such that $\int _{{\mathcal {T}}_xM}pf=0$ for all $p\in {\mathcal {P}}({\mathcal {T}}_xM)$.

In view of Lemma 3.6, every cosymbol $a\in \Sigma _x^s(E,F)$ can be represented in the form

32 $$\begin{aligned} a=k+p\log |\exp ^{-1}(-)| \end{aligned}$$

where $k\in {\mathcal {K}}({\mathcal {T}}_xM;E_x,F_x)$ is homogeneous of order *s*, that is $(\delta _\lambda )_*k=\lambda ^sk$ for all $\lambda >0$, and $p\in {\mathcal {P}}^s({\mathcal {T}}_xM;E_x,F_x)$. If $-s-n\notin {\mathbb {N}}_0$, then $p=0$ and *k* is unique. If $-s-n\in {\mathbb {N}}_0$, then *p* is unique but *k* comes with an ambiguity in ${\mathcal {P}}^s({\mathcal {T}}_xM;E_x,F_x)$. Hence, the restriction of the left regular representation,

33 $$\begin{aligned} {\mathcal {S}}_0({\mathcal {T}}_xM;E_x)\rightarrow {\mathcal {S}}_0({\mathcal {T}}_xM;F_x),\qquad f\mapsto a*f, \end{aligned}$$

does not depend on the representative for $a\in \Sigma ^s_x(E,F)$, provided it is of the form (32).

Suppose $\pi :{\mathcal {T}}_xM\rightarrow U({\mathcal {H}})$ is a non-trivial irreducible unitary representation of the osculating group ${\mathcal {T}}_xM$ on a Hilbert space ${\mathcal {H}}$. Let ${\mathcal {H}}_0$ denote the subspace of ${\mathcal {H}}$ spanned by elements of the form $\pi (fdg)v$ where $v\in {\mathcal {H}}$, $f\in {\mathcal {S}}_0({\mathcal {T}}_xM)$, *dg* denotes a (left) invariant volume density on ${\mathcal {T}}_xM$, and $\pi (fdg):=\int _{{\mathcal {T}}_xM}\pi (g)f(g)dg\in {\mathcal {K}}({\mathcal {H}})$. Since ${\mathcal {H}}_0$ non-trivial and invariant under $\pi (g)$ for all $g\in {\mathcal {T}}_xM$, the subspace ${\mathcal {H}}_0$ is dense in ${\mathcal {H}}$. Note that ${\mathcal {H}}_0\otimes E_x$ is spanned by vectors of the form $\pi (fdg)v$ where $v\in {\mathcal {H}}$, $f\in {\mathcal {S}}_0({\mathcal {T}}_xM;E_x)$, and $\pi (fdg):=\int _{{\mathcal {T}}_xM}\pi (g)f(g)dg\in {\mathcal {K}}({\mathcal {H}},{\mathcal {H}}\otimes E_x)$.

Still assuming a representative of the form (32), we define an unbounded operator $\pi (a)$ from ${\mathcal {H}}\otimes E_x$ to ${\mathcal {H}}\otimes F_x$ by

$$\begin{aligned} \pi (a):{\mathcal {H}}_0\otimes E_x\rightarrow {\mathcal {H}}_0\otimes F_x,\qquad \pi (a)\pi (fdg)v:=\pi (a*fdg)v, \end{aligned}$$

for all $f\in {\mathcal {S}}_0({\mathcal {T}}_xM;E_x)$ and $v\in {\mathcal {H}}$, cf. (33). As explained in [17, Section 2], this definition of $\pi (a)$ is unambiguous. Moreover, $\pi (a)$ is closeable, for $\pi (a^*)$ is a densely defined adjoint. We denote its closure by ${\bar{\pi }}(a)$. It is well known, see [17, 36], or [59, Proposition 3.3.6], that the domain of definition of ${\bar{\pi }}(a)$ contains the space of smooth vectors, ${\mathcal {H}}_\infty \otimes E_x$, and this subspace is mapped into ${\mathcal {H}}_\infty \otimes F_x$ by ${\bar{\pi }}(a)$. Furthermore, on ${\mathcal {H}}_\infty \otimes E_x$, we have

34 $$\begin{aligned} {\bar{\pi }}(ba)={\bar{\pi }}(b){\bar{\pi }}(a) \end{aligned}$$

whenever $a\in \Sigma ^s_x(E,F)$ and $b\in \Sigma ^{{\tilde{s}}}_x(F,G)$. Moreover, with respect to inner products on $E_x$ and $F_x$ and the associated inner products on ${\mathcal {H}}\otimes E_x$ and ${\mathcal {H}}\otimes F_x$, we have

35 $$\begin{aligned} {\bar{\pi }}(a)^*={\bar{\pi }}(a^*) \end{aligned}$$

on ${\mathcal {H}}_\infty \otimes F_x$. If *a* is the cosymbol of a differential operator then, on ${\mathcal {H}}_\infty \otimes E_x$, the operator ${\bar{\pi }}(a)$ coincides with $\pi (a)$ considered in Sect. 2.2, see [59, Remark 3.3.7], and thus the following definition is consistent with Definition 2.5.

#### Definition 3.8

(*Matrix Rockland condition*) A principal cosymbol $a\in \Sigma _x^s(E,F)$ at $x\in M$ is said to satisfy the *Rockland condition* if, for every non-trivial irreducible unitary representation $\pi :{\mathcal {T}}_xM\rightarrow U({\mathcal {H}})$, the unbounded operator ${\bar{\pi }}(a)$ is injective on ${\mathcal {H}}_\infty \otimes E_x$. An operator $A\in \Psi ^s(E,F)$ is said to satisfy the Rockland condition if its principal cosymbol, $\sigma ^s_x(A)\in \Sigma ^s_x(E,F)$, satisfies the Rockland condition at each point $x\in M$.

We will need the following matrix version of a Rockland [62] type theorem due to Christ et al., see [17, Theorem 6.2] or [36] for the order zero case. For differential operators such a statement can be found in van Erp’s thesis [75].

#### Lemma 3.9

(Christ et al.) A principal cosymbol $a\in \Sigma _x^s(E,F)$ at $x\in M$ satisfies the Rockland condition iff it admits a left inverse $b\in \Sigma _x^{-s}(F,E)$, that is, $ba=1$.

#### Proof

The necessity of the Rockland condition is obvious, see (34). To prove the non-trivial implication, we will present an elementary argument, reducing the statement to the well-known scalar case due to Christ et al. , see [17, Theorem 6.2]. For $s=0$ this has been proved in [36].

With respect to bases of $E_x$ and $F_x$, the cosymbol $a\in \Sigma ^s_x(E,F)$ corresponds to a matrix *A* with entries in $\Sigma ^s_x$, the space of principal cosymbols of order *s* at *x* for scalar operators. Moreover, ${\bar{\pi }}(a)$ corresponds to the matrix ${\bar{\pi }}(A)$ obtained by applying the representation $\pi$ to each entry of *A*, that is, $({\bar{\pi }}(A))_{ij}={\bar{\pi }}(A_{ij})$. Hence, *a* satisfies the Rockland condition in Definition 3.8 iff the matrix ${\bar{\pi }}(A)$ acts injectively on $({\mathcal {H}}_\infty )^m$. Here $m:=\dim (E_x)$ is the number of columns of *A*. Using induction on *m*, we will show that there exists a matrix *B* with entries in $\Sigma ^{-s}_x$ such that $BA=I_m$ where $I_m$ denotes the $m\times m$ unit matrix.

Clearly, $A^*A$ is an $(m\times m)$-matrix with entries in $\Sigma ^{2s}_x$ which satisfies the Rockland condition, see (34) and (35). Let $y:=\sum _j(A_{j1})^*A_{j1}\in \Sigma ^{2s}_x$ denote the entry in the upper left corner of $A^*A$. Clearly, *y* satisfies the (scalar) Rockland condition. Hence, according to [17, Theorem 6.2], there exists $z\in \Sigma ^{-2s}_x$ such that $zy=1$. Since $y^*=y$, we also have $yz=1$, whence *z* is invertible. Hence, the diagonal matrix $D:=zI_m$ is invertible and, thus, $DA^*A$ satisfies the Rockland condition. The matrix $DA^*A$ has entries in $\Sigma ^0_x$ and, by construction, its entry in the upper left corner is 1. Performing elementary row operations, we find an invertible matrix *L* with entries in $\Sigma ^0_x$ such that

$$\begin{aligned} LDA^*A=\begin{pmatrix}1&{}*\\ 0&{}Y\end{pmatrix} \end{aligned}$$

where *Y* is an $(m-1)\times (m-1)$-matrix with entries in $\Sigma ^0_x$. Since *L* is invertible, $LDA^*A$ satisfies the Rockland condition and, thus, *Y* satisfies the Rockland condition too. By induction, there exists an $(m-1)\times (m-1)$-matrix *Z* with entries in $\Sigma ^0_x$ such that $ZY=I_{m-1}$. The matrix $$C:=\begin{pmatrix}1&{}0\\ 0&{}Z\end{pmatrix}$$ has entries in $\Sigma ^0_x$ and, by construction,

$$\begin{aligned} CLDA^*A=\begin{pmatrix}1&{}*\\ 0&{}I_{m-1}\end{pmatrix}. \end{aligned}$$

Performing further elementary row operations, we find an invertible matrix *U* with entries in $\Sigma ^0_x$ such that $UCLDA^*A=I_m$. Hence, the matrix $B:=UCLDA^*$ has entries in $\Sigma ^{-s}_x$ and satisfies $BA=I_m$. $\square$

#### Lemma 3.10

Let *E* and *F* be two vector bundles over a filtered manifold *M*. Consider $a\in \Sigma ^s(E,F)$ and suppose ${{\,\mathrm{ev}\,}}_{x_0}(a)$ is left invertible at some point $x_0\in M$, that is, there exists $b_{x_0}\in \Sigma _{x_0}^{-s}(F,E)$ such that $b_{x_0}{{\,\mathrm{ev}\,}}_{x_0}(a)=1$. Then, there exists an open neighborhood *U* of $x_0$ and $b\in \Sigma ^{-s}(F|_U,E|_U)$ such that $ba|_U=1$. Moreover, a similar statement involving right inverses holds true.

#### Proof

If the bundle of osculating algebras is locally trivial, then this follows from [17], at least in the scalar case. To handle general bundles of osculating algebras, we will proceed as in [59, Section 3.3.3] where Ponge considers (in general non-contact) Heisenberg manifolds with varying osculating algebras, see also [52, Section 6].

Choose ${\tilde{b}}\in \Sigma ^{-s}(F,E)$ such that ${{\,\mathrm{ev}\,}}_{x_0}({\tilde{b}})=b_{x_0}$. Putting $c:={\tilde{b}}a$, we have $c\in \Sigma ^0(E)$ and ${{\,\mathrm{ev}\,}}_{x_0}(c)=1$. It suffices to find an open neighborhood *U* of $x_0$ in *M* such that $c|_U$ is invertible in $\Sigma ^0(E|_U)$, for then $b:=c|_U^{-1}{\tilde{b}}$ is the desired local left inverse of *a*.

We will identify $\Sigma ^0(E)={\hat{\Sigma }}^0(E)$ where ${\hat{\Sigma }}^0(E)$ denotes the space of all $k\in {\mathcal {K}}(\mathcal {T}M;E,E)$ which are strictly homogeneous of order zero, that is, $(\delta _\lambda )_*k=k$ for all $\lambda >0$, see Lemma 3.6. Convolution with $k\in {\hat{\Sigma }}^0_x(E)$ gives rise to a bounded operator on $L^2({\mathcal {T}}_xM)\otimes E_x$, see [34, Theorem 6.19]. We fix an auxiliary fiber-wise Hermitian inner product on *E*, as well as a smooth family of invariant volume densities on the osculating groups ${\mathcal {T}}_xM$, and let $|||k|||_x$ denote the operator norm with respect to the associated Hermitian inner product on $L^2({\mathcal {T}}_xM)\otimes E_x$.

We fix a fiber-wise homogeneous norm $|-|$ on $\mathcal {T}M$ which is smooth on $\mathcal {T}M\setminus M$. This permits decomposing each $k\in {\hat{\Sigma }}^0_x(E)$ uniquely in the form $k=c_x(k)\delta _x+{{\,\mathrm{pv}\,}}_x(k)$ where $c_x(k)\in {{\,\mathrm{end}\,}}(E_x)$, and ${{\,\mathrm{pv}\,}}_x(k)\in {\hat{\Sigma }}^0_x(E)$ is the principal value distribution

$$\begin{aligned} \langle {{\,\mathrm{pv}\,}}_x(k),\psi \rangle :=\lim _{\varepsilon \rightarrow 0}\int _{\{g\in {\mathcal {T}}_xM:|g|\ge \varepsilon \}}k\psi , \end{aligned}$$

see [34, Proposition 6.13]. For each $r\in {\mathbb {N}}_0$ we consider the norm

$$\begin{aligned} \Vert k\Vert _{x,r}:=|c_x(k)|+\left( \int _{{\mathcal {S}}_xM}|j^r(k|_{{\mathcal {S}}_xM})|^2\mu _x\right) ^{1/2}, \end{aligned}$$

where $k\in {\hat{\Sigma }}^0_x(E)$. Here $j^r(k|_{{\mathcal {S}}_xM})$ denotes the *r*-jet of the restriction of *k* to the sphere ${\mathcal {S}}_xM:=\{g\in {\mathcal {T}}_xM:|g|=1\}$, and we use smooth fiber-wise Hermitian metric on the *r*-jet bundle $J^r({\mathcal {S}}_xM,E_x)$ which depends smoothly on *x*, and we are using a smooth volume density $\mu _x$ on the sphere ${\mathcal {S}}_xM$ which depends smoothly on *x*.

There exists $r_0\in {\mathbb {N}}_0$ and constants $C_x\ge 0$ such that $|||k|||_x\le C_x\Vert k\Vert _{x,r_0}$ for all $k\in {\hat{\Sigma }}^0_x(E)$. This follows from a result due to Folland and Stein, see [34, Theorem 6.19], and the Sobolev embedding theorem. Ponge observed, see [59, Lemma 3.3.13], that the proof of Folland and Stein allows choosing the constants $C_x$ uniformly over compact subsets of *M*. To make this more precise, we put, for every compact subset *L* of *M* and each $k\in {\hat{\Sigma }}^0(E)$,

$$\begin{aligned} |||k|||_L:=\sup _{x\in L}|||{{\,\mathrm{ev}\,}}_x(k)|||_x\qquad \text {and}\qquad \Vert k\Vert _{L,r}:=\sup _{x\in L}\Vert {{\,\mathrm{ev}\,}}_x(k)\Vert _{x,r}. \end{aligned}$$

Then, there exists a constant $C_L\ge 0$ such that

36 $$\begin{aligned} |||k|||_L\le C_L\Vert k\Vert _{L,r_0} \end{aligned}$$

holds for all $k\in {\hat{\Sigma }}^0(E)$.^2^ Moreover, see [59, Lemma 3.3.15] and [17, Lemma 5.7], for every $r\ge r_0$ there exists a constant $C_{L,r}\ge 0$ such that

$$\begin{aligned} \Vert k_2*k_1\Vert _{L,r}\le C_{L,r}\Bigl (\Vert k_2\Vert _{L,r}\cdot |||k_1|||_L+|||k_2|||_L\cdot \Vert k_1\Vert _{L,r}\Bigr ) \end{aligned}$$

holds for all $k_1,k_2\in {\hat{\Sigma }}^0(E)$. As in [16, Lemma 4], this gives

$$\begin{aligned} \Vert k^{2i}\Vert ^{1/2i}_{L,r}\le (2C_{L,r})^{1/2i}\sqrt{|||k^i|||^{1/i}_L}\sqrt{\Vert k^i\Vert _{L,r}^{1/i}}\le (2C_{L,r})^{1/2i}\sqrt{|||k|||}_L\sqrt{\Vert k^i\Vert _{L,r}^{1/i}} \end{aligned}$$

and passing to the limit, we obtain

37 $$\begin{aligned} \lim _{i\rightarrow \infty }\Vert k^i\Vert ^{1/i}_{L,r}\le |||k|||_L \end{aligned}$$

for all $r\ge r_0$ and all $k\in {\hat{\Sigma }}^0(E)$.

To invert *c* we write $c=1-k$ where $k\in {\hat{\Sigma }}^0(E)$. Then ${{\,\mathrm{ev}\,}}_{x_0}(k)=0$, and there exists a compact neighborhood *L* of $x_0$ such that $|||k|||_L<1$, see (36). In view of (37), the Neumann series $\sum _{i=0}^\infty k^i$ converges with respect to the norm $\Vert -\Vert _{L,r}$ for all $r\ge r_0$. For each $x\in L$ we conclude that ${{\,\mathrm{ev}\,}}_x(c)$ is invertible in ${\hat{\Sigma }}^0_x(E)$ with inverse ${{\,\mathrm{ev}\,}}_x(c)^{-1}=\sum _{i=0}^\infty {{\,\mathrm{ev}\,}}_x(k)^i$. This also shows that ${{\,\mathrm{ev}\,}}_x(c)^{-1}$ depends continuously on $x\in L$. Proceeding as in the proof of [59, Proposition 3.3.11], we see that ${{\,\mathrm{ev}\,}}_x(c)^{-1}$ actually depends smoothly on *x* in the interior *U* of *L*, see also [17, Proposition 5.10]. Hence, $c|_U$ is invertible in ${\hat{\Sigma }}^0(E|_U)$ with inverse $c|_U^{-1}=\sum _{i=0}^\infty k|_U^i$. $\square$

Combining these results, we obtain the following Rockland [62] type theorem generalizing Melin’s result for scalar differential operators [52, Theorem 7.1], see also [17, Theorem 2.5(d)], [43, Theorem 0.1] and [59, Theorem 3.3.10 and 5.4.1].

#### Theorem 3.11

Let *E* and *F* be vector bundles over a filtered manifold *M*. Let *s* be a complex number, and suppose $A\in \Psi ^s(E,F)$ satisfies the Rockland condition. Then, there exists a left parametrix $B\in \Psi ^{-s}_\text {prop}(F,E)$ such that $BA-{{\,\mathrm{id}\,}}$ is a smoothing operator. In particular, *A* is hypoelliptic. If, moreover, *M* is closed, then $\ker (A)$ is a finite dimensional subspace of $\Gamma ^\infty (E)$.

#### Proof

According to Lemma 3.9 the principal cosymbol $\sigma ^s_x(A)$ admits a left inverse at each point $x\in M$. Hence, in view of Lemma 3.10, we see that the principal cosymbol $\sigma ^s(A)$ locally admits left inverses. Using a smooth partition of unity on *M*, we obtain a global left inverse $b\in \Sigma ^{-s}(F,E)$ such that $b\sigma ^s(A)=1$. Applying Proposition 3.1(g), we obtain $B\in \Psi ^{-s}_\text {prop}(F,E)$ such that $BA-{{\,\mathrm{id}\,}}$ is a smoothing operator. The remaining assertions are now obvious, cf. the proof of Corollary 2.8 above. $\square$

Note that Theorem 2.6 follows immediately from Theorem 3.11.

#### Corollary 3.12

Let *E* be a vector bundle over a closed filtered manifold *M*. Suppose $A\in \Psi ^s(E)$ satisfies the Rockland condition and is formally self-adjoint, $A^*=A$, with respect to an $L^2$ inner product of the form (11), and let *Q* denote the orthogonal projection onto the (finite dimensional) subspace $\ker (A)\subseteq \Gamma ^\infty (E)$. Then, $A+Q$ is invertible with inverse $(A+Q)^{-1}\in \Psi ^{-s}(E)$.

#### Proof

Proceeding exactly as in the proof of Corollary 2.9, we start with a left parametrix $B\in \Psi ^{-s}(E)$, see Theorem 3.11, and observe that the injective and formally self-adjoint parametrix *P* constructed there is contained in $\Psi ^{-s}(E)$. As explained in the proof of Corollary 2.9, $G:=(A+Q)P\in \Psi ^0(E)$ is invertible, and $G^{-1}-{{\,\mathrm{id}\,}}$ is a smoothing operator. Clearly, this implies $G^{-1}\in \Psi ^0(E)$, whence $(A+Q)^{-1}=PG^{-1}\in \Psi ^{-s}(E)$. $\square$

Combining these observations with a result from [17] permits constructing operators of arbitrary order which are invertible up to smoothing operators. More precisely, we have:

#### Lemma 3.13

Let *E* be a vector bundle over a filtered manifold *M*. Then, for every complex number *s*, there exist $\Lambda \in \Psi ^s_\text {prop}(E)$ and $\Lambda '\in \Psi ^{-s}_\text {prop}(E)$ such that $\Lambda \Lambda '-{{\,\mathrm{id}\,}}$ and $\Lambda '\Lambda -{{\,\mathrm{id}\,}}$ are both smoothing operators. Moreover, $\Lambda$ and $\Lambda '$ may be chosen so that they act injectively on $\Gamma ^{-\infty }_c(E)$. For closed *M*, there exist $\Lambda \in \Psi ^s(E)$ and $\Lambda '\in \Psi ^{-s}(E)$ such that $\Lambda \Lambda '={{\,\mathrm{id}\,}}=\Lambda '\Lambda$.

#### Proof

For each $x\in M$, we fix an invertible cosymbol $a_x\in \Sigma ^{s/4}_x(E)$ at *x*, see [17, Theorem 6.1]. We extend these to cosymbols ${\tilde{a}}_x\in \Sigma ^{s/4}(E)$ such that ${{\,\mathrm{ev}\,}}_x({\tilde{a}}_x)=a_x$. By Lemma 3.10, for each $x\in M$, there exists an open neighborhood $U_x$ of *x* such that ${\tilde{a}}_x|_{U_x}$ is invertible in $\Sigma ^{s/4}(E|_{U_x})$. Fix a smooth partition of unity $\lambda _x$, $x\in M$, such that ${{\,\mathrm{supp}\,}}(\lambda _x)\subseteq U_x$ for each $x\in M$. With respect to a fiber-wise Hermitian metric on *E*, we consider

$$\begin{aligned} a:=\sum _{x\in M}\lambda _x{\tilde{a}}_x^*{\tilde{a}}_x\in \Sigma ^{s/2}(E). \end{aligned}$$

We claim that *a* is an invertible cosymbol. To see this note first that, in view of Lemma 3.10, it suffices to show that ${{\,\mathrm{ev}\,}}_y(a)$ admits an inverse in $\Sigma ^{-s/2}_y(E)$ for each $y\in M$. For fixed $y\in M$, there exists $x\in M$ such that $\lambda _x(y)>0$ and, by construction, ${{\,\mathrm{ev}\,}}_y({\tilde{a}}_x)$ is invertible. Using (34) and (35) we conclude that ${{\,\mathrm{ev}\,}}_y(a)$ satisfies the Rockland condition and, thus, admits a left inverse in $\Sigma ^{-s/2}_y(E)$, see Lemma 3.9. Actually, ${{\,\mathrm{ev}\,}}_y(a)$ is invertible since ${{\,\mathrm{ev}\,}}_y(a)={{\,\mathrm{ev}\,}}_y(a)^*$. This shows that *a* is invertible; hence, there exists $a'\in \Sigma ^{-s/2}(E)$ such that $aa'=1=a'a$.

According to Proposition 3.1(b) there exist $A\in \Psi ^{s/2}_\text {prop}(E)$ such that $\sigma ^{s/2}(A)=a$. Using Proposition 3.1(g) we obtain $A'\in \Psi ^{-s/2}_\text {prop}(E)$ such that $R:=A'A-{{\,\mathrm{id}\,}}$ is a smoothing operator. Proposition 3.1(g) also gives $A''\in \Psi _\text {prop}^{-s/2}(E)$ such that $AA''-{{\,\mathrm{id}\,}}$ is a smoothing operator. Since $A''$ differs from $A'$ by a smoothing operator, $AA'-{{\,\mathrm{id}\,}}$ is a smoothing operator too.

Consider $\Lambda \in \Psi ^s_\text {prop}(E)$ and $\Lambda '\in \Psi ^{-s}_\text {prop}(E)$ defined by

$$\begin{aligned} \Lambda :=A^*A+R^*R\qquad \text {and}\qquad \Lambda ':=A'(A')^*+RR^*, \end{aligned}$$

where the adjoints are with respect to an $L^2$ inner product of the form (11). One readily verifies that $\Lambda '\Lambda -{{\,\mathrm{id}\,}}$ and $\Lambda \Lambda '-{{\,\mathrm{id}\,}}$ are both smoothing operators. In particular, $\Lambda$ is hypoelliptic and, thus, every distributional section in the kernel of $\Lambda$ has to be smooth. Using (10), we conclude, $\ker (\Lambda |_{\Gamma ^{-\infty }_c(E)})\subseteq \ker (A)\cap \ker (R)\subseteq \ker ({{\,\mathrm{id}\,}})=0$. Analogously, $\ker (\Lambda '|_{\Gamma ^{-\infty }_c(E)})\subseteq \ker ((A')^*)\cap \ker (R^*)\subseteq \ker ({{\,\mathrm{id}\,}})=0$ in view of $R^*=A^*(A')^*-{{\,\mathrm{id}\,}}$. If the underlying manifold is closed, then $\Lambda ^{-1}\in \Psi ^{-s}(E)$ according to Corollary 3.12. $\square$

#### Remark 3.14

(*Right parametrix*) If $A\in \Psi ^s(E,F)$ and $A^t$ satisfies the Rockland condition, then there exists a right parametrix $B\in \Psi _\text {prop}^{-s}(F,E)$ such that $AB-{{\,\mathrm{id}\,}}$ is a smoothing operator. Indeed, by Theorem 3.11 there exists $B'\in \Psi _\text {prop}^{-s}(E',F')$ such that $B'A^t-{{\,\mathrm{id}\,}}$ is a smoothing operator. Hence, $B:=(B')^t$ is the desired right parametrix for *A*.

#### Corollary 3.15

If $A\in \Psi ^s(E,F)$ is such that *A* and $A^t$ both satisfy the Rockland condition, then there exists a parametrix $B\in \Psi _\text {prop}^{-s}(F,E)$ such that $AB-{{\,\mathrm{id}\,}}$ and $BA-{{\,\mathrm{id}\,}}$ are both smoothing operators. In particular, the principal cosymbol $\sigma ^s(A)$ is invertible.

#### Proof

This follows immediately from Theorem 3.11 and Remark 3.14 since every left parametrix differs from any right parametrix by a smoothing operator. $\square$

#### Remark 3.16

For $A\in \Psi ^s(E,F)$ and $x\in M$ the following are equivalent:

- $A^t$ satisfies the Rockland condition at *x*.
- $A^*$ satisfies the Rockland condition at *x*.
- ${\bar{\pi }}(\sigma _x^s(A)):{\mathcal {H}}_\infty \otimes E_x\rightarrow {\mathcal {H}}_\infty \otimes F_x$ has dense image, for all non-trivial irreducible unitary representations $\pi :{\mathcal {T}}_xM\rightarrow U({\mathcal {H}})$.
- ${\bar{\pi }}(\sigma _x^s(A)):{\mathcal {H}}_\infty \otimes E_x\rightarrow {\mathcal {H}}_\infty \otimes F_x$ is onto, for all non-trivial irreducible unitary representations $\pi :\mathcal {T}_xM\rightarrow U({\mathcal {H}})$.

Indeed, the equivalence (a)$\Leftrightarrow$(b) is clear in view of (12). The equivalence (b)$\Leftrightarrow$(c) follows from ${\bar{\pi }}(\sigma ^s_x(A))^*={\bar{\pi }}(\sigma ^{{\bar{s}}}_x(A^*))$, see (35) and Remark 3.3. To see the implication (a)$\Rightarrow$(d) suppose $A^t$ satisfies the Rockland condition at *x*. According to Lemma 3.9, there exists $b\in \Sigma ^{-s}_x(E',F')$ such that $b\sigma ^s_x(A^t)=1$. Transposing this equation, we obtain $\sigma ^s_x(A)b^t=1$, see Proposition 3.1(e). In view of (34), this implies (d), for ${\bar{\pi }}(b^t)$ maps ${\mathcal {H}}_\infty \otimes F_x$ into ${\mathcal {H}}_\infty \otimes E_x$.

### The Heisenberg Sobolev scale

The properties of the operator class $\Psi ^s(E,F)$ discussed above permit introducing a Heisenberg Sobolev scale on filtered manifolds which can be used to refine the hypoellipticity results in Sect. 2.3, see Corollaries 3.24 and 3.25 at the end of this section. The main properties of this Sobolev scale are summarized in Proposition 3.17, a refined regularity statement including maximal hypoelliptic estimates can be found in Corollary 3.20.

For non-degenerate CR manifolds the origins of this Sobolev scale can be traced back to a paper of Folland and Stein [33] where $L^p$ Sobolev spaces for integral *s* are constructed using differential operators, see [33, Section 15], and maximal hypoelliptic estimates for Kohn’s Laplacian are established, see [33, Theorem 16.6] and also [33, Theorem 16.7]. For Heisenberg manifolds satisfying only the bracket generating condition $H+[H,H]=TM$, a full Sobolev scale has been constructed by Ponge using complex powers of subLaplacians, see [59, Section 5.5] and [59, Propositions 5.5.9 ad 5.5.14]. Maximal hypoelliptic estimates can also be found in [44] and the work of Beals and Greiner, see [3, Theorem 18.31]. A full Sobolev scale for stratified Lie groups has been constructed in [32].

For every real *s* we let $H^s_\text {loc}(E)$ denote the space of all distributional sections $\psi \in \Gamma ^{-\infty }(E)$ such that $A\psi \in L^2_\text {loc}(F)$ for all $A\in \Psi ^s_\text {prop}(E,F)$ and all vector bundles *F*. We equip $H^s_\text {loc}(E)$ with the coarsest topology such that the maps $A:H^s_\text {loc}(E)\rightarrow L^2_\text {loc}(F)$ are continuous, for all $A\in \Psi _\text {prop}^s(E,F)$. Analogously, let $H^s_c(E)$ denote the space of compactly supported distributional sections $\psi \in \Gamma _c^{-\infty }(E)$ such that $A\psi \in L^2_\text {loc}(F)$ for all $A\in \Psi ^s(E,F)$, and equip $H^s_c(E)$ with the coarsest topology such that the corresponding maps $A:H^s_c(E)\rightarrow L^2_\text {loc}(F)$ are continuous, for all $A\in \Psi ^s(E,F)$ and all vector bundles *F*.

#### Proposition 3.17

Let *E* and *F* be vector bundles over a filtered manifold *M*.

- $H^s_\text {loc}(E)$ is a complete locally convex vector space, and we have continuous inclusions
  $$\begin{aligned} \Gamma ^\infty (E)\subseteq H^{s_2}_\text {loc}(E)\subseteq H^{s_1}_\text {loc}(E)\subseteq \Gamma ^{-\infty }(E) \end{aligned}$$
  whenever $s_1\le s_2$. Moreover, $H^0_\text {loc}(E)=L^2_\text {loc}(E)$ as locally convex spaces.
- $H^s_c(E)$ is a complete locally convex vector space, and we have continuous inclusions
  $$\begin{aligned} \Gamma ^\infty _c(E)\subseteq H^{s_2}_c(E)\subseteq H^{s_1}_c(E)\subseteq \Gamma ^{-\infty }_c(E) \end{aligned}$$
  whenever $s_1\le s_2$. Moreover, $H^0_c(E)=L^2_c(E)$ as locally convex spaces.
- The canonical pairing $\Gamma ^\infty _c(E')\times \Gamma ^\infty (E)\rightarrow {\mathbb {C}}$ extends to a pairing
  $$\begin{aligned} H^{-s}_c(E')\times H^s_\text {loc}(E)\rightarrow {\mathbb {C}}\end{aligned}$$
  inducing linear bijections $H^s_\text {loc}(E)^*=H^{-s}_c(E')$ and $H^{-s}_c(E')^*=H^s_\text {loc}(E)$. If, moreover, *M* is closed, then $H^s_c(E)=H^s_\text {loc}(E)$ is a Hilbert space we denote by $H^s(E)$, and the pairing induces an isomorphism of Hilbert spaces, $H^s(E)^*=H^{-s}(E')$.
- Each $A\in \Psi ^r(E,F)$ restricts to continuous operator $A:H^s_c(E)\rightarrow H_\text {loc}^{s-\Re (r)}(F)$. On a closed manifold we obtain a bounded operator $A:H^s(E)\rightarrow H^{s-\Re (r)}(F)$.
- Assume *M* closed and let $\Lambda _s\in \Psi ^s(E)$ be invertible with $\Lambda _s^{-1}\in \Psi ^{-s}(E)$, see Lemma 3.13. If $\langle \!\langle -,-\rangle \!\rangle _{L^2(E)}$ is any Hermitian inner product generating the topology on $L^2(E)$, then
  38 $$\begin{aligned} \langle \!\langle \psi _1,\psi _2\rangle \!\rangle _{H^s(E)}:=\langle \!\langle \Lambda _s\psi _1,\Lambda _s\psi _2\rangle \!\rangle _{L^2(E)}, \end{aligned}$$
  $\psi _1,\psi _2\in H^s(E)$, is a Hermitian inner product generating the topology on $H^s(E)$.
- If $s_1<s_2$, then the inclusions $H^{s_2}_\text {loc}(E)\subseteq H^{s_1}_\text {loc}(E)$ and $H^{s_2}_c(E)\subseteq H^{s_1}_c(E)$ are compact. For closed *M* we obtain a compact inclusion $H^{s_2}(E)\subseteq H^{s_1}(E)$.
- If *M* has homogeneous dimension *n*, see (29), then we have continuous Sobolev embeddings $H^s_\text {loc}(E)\subseteq \Gamma ^r(E)$ and $H^s_c(E)\subseteq \Gamma ^r_c(E)$ for all $r\in {\mathbb {N}}_0$ with $r<s-n/2$, see Remark 2.1. In particular, we obtain isomorphisms of locally convex spaces,
  $$\begin{aligned} \Gamma ^\infty (E)=\bigcap _s H^s_\text {loc}(E) \qquad \text {and}\qquad \Gamma _c^\infty (E)=\bigcap _s H^s_c(E), \end{aligned}$$
  as well as $\Gamma ^{-\infty }(E)=\bigcup _sH^s_\text {loc}(E)$ and $\Gamma ^{-\infty }_c(E)=\bigcup _sH^s_c(E)$.

#### Proof

The proof is a routine extension, cf. for instance [72, Section §7] for the classical case, of the results established in the preceding sections. For each complex number *s* we choose operators $\Lambda _s\in \Psi ^s_\text {prop}(E)$, $\Lambda '_s\in \Psi ^{-s}_\text {prop}(E)$, and $R_s\in {\mathcal {O}}^{-\infty }_\text {prop}(E)$ such that, see Lemma 3.13,

$$\begin{aligned} \Lambda _s'\Lambda _s={{\,\mathrm{id}\,}}+R_s. \end{aligned}$$

We have a continuous inclusion $\Gamma ^\infty (E)\subseteq H^s_\text {loc}(E)$ since every operator $A\in \Psi ^s_\text {prop}(E,F)$ induces a continuous map $A:\Gamma ^\infty (E)\rightarrow \Gamma ^\infty (F)$, see Proposition 3.1(a), and the inclusion $\Gamma ^\infty (F)\subseteq L^2_\text {loc}(F)$ is continuous. Using Proposition 3.1(a) we see that the composition $H^s_\text {loc}(E)\xrightarrow {\Lambda _s}L^2_\text {loc}(E)\subseteq \Gamma ^{-\infty }(E)\xrightarrow {\Lambda '_s}\Gamma ^{-\infty }(E)$ is continuous. Moreover, ${\mathcal {O}}^{-\infty }_\text {prop}(E)\subseteq \Psi ^s_\text {prop}(E)$, see Proposition 3.1(c), and thus the composition $H^s_\text {loc}(E)\xrightarrow {R_s}L^2_\text {loc}(E)\subseteq \Gamma ^{-\infty }(E)$ is continuous. We conclude that ${{\,\mathrm{id}\,}}=\Lambda _s'\Lambda _s-R_s$ induces a continuous map $H^s_\text {loc}(E)\subseteq \Gamma ^{-\infty }(E)$. To see that we have continuous inclusions $H^{s_2}_\text {loc}(E)\subseteq H^{s_1}_\text {loc}(E)$ for all $s_1\le s_2$, we have to show that each $A\in \Psi ^{s_1}_\text {prop}(E,F)$ induces a continuous operator $A:H^{s_2}_\text {loc}(E)\rightarrow L^2_\text {loc}(E)$. To achieve that, note that $A=\Lambda '_{s_2-s_1}\Lambda _{s_2-s_1}A-R_{s_2-s_1}A$. Moreover, $\Lambda _{s_2-s_1}A\in \Psi ^{s_2}_\text {prop}(E,F)$, see Proposition 3.1(d), and thus $\Lambda _{s_2-s_1}A:H^{s_2}_\text {loc}(E)\rightarrow L^2_\text {loc}(F)$ is continuous. Moreover, $\Lambda '_{s_2-s_1}$ induces a continuous operator $L^2_\text {loc}(F)\rightarrow L^2_\text {loc}(F)$ since $s_1-s_2\le 0$, see Proposition 3.7(a). Furthermore, $R_{s_2-s_1}A\in {\mathcal {O}}^{-\infty }_\text {prop}(E,F)\subseteq \Psi ^{s_2}_\text {prop}(E,F)$ in view of Proposition 3.1(c) and thus $R_{s_2-s_1}A:H^{s_2}_\text {loc}(E)\rightarrow L^2_\text {loc}(F)$ is continuous. We conclude that $A=\Lambda '_{s_2-s_1}\Lambda _{s_2-s_1}A-R_{s_2-s_1}A$ induces a continuous map $H^{s_2}_\text {loc}(E)\rightarrow L^2_\text {loc}(F)$, whence the continuous inclusion $H^{s_2}_\text {loc}(E)\subseteq H^{s_1}_\text {loc}(E)$. The completeness of $H^s_\text {loc}(E)$ follows from the continuity of the inclusion $H^s_\text {loc}(E)\subseteq \Gamma ^{-\infty }(E)$, the completeness of the spaces $\Gamma ^{-\infty }(E)$, the fact that each $A\in \Psi ^s_\text {prop}(E,F)$ induces a continuous operator $A:\Gamma ^{-\infty }(E)\rightarrow \Gamma ^{-\infty }(F)$, see Proposition 3.1(a), and the completeness of the spaces $L^2_\text {loc}(F)$. We have a continuous inclusion $H^0_\text {loc}(E)\subseteq L^2_\text {loc}(E)$ since ${{\,\mathrm{id}\,}}\in \Psi ^0_\text {prop}(E)$, see Proposition 3.1(f). By Proposition 3.7(a), we also have the converse continuous inclusion $L^2_\text {loc}(E)\subseteq H^0_\text {loc}(E)$. This shows $H^0_\text {loc}(E)=L^2_\text {loc}(E)$ and completes the proof of (a). Part (b) can be proved analogously.

To see (c), note that the canonical pairing can be written as

39 $$\begin{aligned} \langle \phi ,\psi \rangle =\langle (\Lambda '_s)^t\phi ,\Lambda _s\psi \rangle -\langle \phi ,R_s\psi \rangle , \end{aligned}$$

where $\phi \in \Gamma ^\infty _c(E')$, $\psi \in \Gamma ^\infty _c(E)$, and $(\Lambda '_s)^t\in \Psi ^{-s}_\text {prop}(E')$ according to Proposition 3.1(e). Recall that the canonical pairing extends to a pairing $L^2_c(E')\times L^2_\text {loc}(E)\rightarrow {\mathbb {C}}$. Since $\Lambda _s:H^s_\text {loc}(E)\rightarrow L^2_\text {loc}(E)$ and $(\Lambda _s')^t:H^{-s}_c(E')\rightarrow L^2_c(E')$ are continuous, we see that the term $\langle (\Lambda '_s)^t\phi ,\Lambda _s\psi \rangle$ extends to a separately continuous bilinear map $H^{-s}_c(E')\times H^s_\text {loc}(E)\rightarrow {\mathbb {C}}$. Since $R_s$ induces a continuous operator $R_s:\Gamma ^{-\infty }_\text {loc}(E)\rightarrow \Gamma ^\infty _\text {loc}(E)$, the term $\langle \phi ,R_s\psi \rangle$ actually extends to a separately continuous bilinear map $\Gamma ^{-\infty }_c(E')\times \Gamma ^{-\infty }_\text {loc}(E)\rightarrow {\mathbb {C}}$. Using (a), (b) and (39) we conclude that the canonical pairing extends to a separately continuous bilinear map $H^{-s}_c(E')\times H^s_\text {loc}(E)\rightarrow {\mathbb {C}}$. Let us now verify that the induced linear map

40 $$\begin{aligned} H^s_\text {loc}(E)\rightarrow H^{-s}_c(E')^* \end{aligned}$$

is bijective. Since the inclusion ${\mathcal {D}}(E)=\Gamma ^\infty _c(E')\subseteq H^{-s}_c(E')$ is continuous, we obtain a continuous map $H^{-s}_c(E')^*\rightarrow {\mathcal {D}}(E)^*=\Gamma ^{-\infty }(E)$ which, when composed with (40), yields the canonical inclusion $H^s_\text {loc}(E)\subseteq \Gamma ^{-\infty }(E)$. This immediately implies that (40) is injective. To see that it is onto, suppose $\alpha \in H^{-s}_c(E')^*$. The preceding considerations show that there exists $\psi \in \Gamma ^{-\infty }(E)$ such that $\langle \phi ,\psi \rangle =\alpha (\phi )$ for all $\phi \in {\mathcal {D}}(E)$. It remains to show that $\psi \in H^s_\text {loc}(E)$. To check this, let $A\in \Psi ^s_\text {prop}(E,F)$. Then $\langle {\tilde{\phi }},A\psi \rangle =\langle A^t{\tilde{\phi }},\psi \rangle =(\alpha \circ A^t)({\tilde{\phi }})$ extends continuously to all ${\tilde{\phi }}\in L^2_c(F')$. Since the pairing $L^2_c(F')\times L^2_\text {loc}(F)\rightarrow {\mathbb {C}}$ induces a linear bijection $L^2_\text {loc}(F)=L^2_c(F')^*$, we conclude $A\psi \in L^2_\text {loc}(F)$, whence $\psi \in H^s_\text {loc}(E)$. Analogously, one can verify that the induced linear map $H^{-s}_c(E')\rightarrow H^s_\text {loc}(E)^*$ is a bijection. If *M* is closed, then $\Lambda _s$ may be assumed to be invertible with inverse $\Lambda _s^{-1}\in \Psi ^{-s}(E)$, see Lemma 3.13; hence, $\Lambda _s$ induces a topological isomorphism $H^s(E)\cong L^2(E)$, whence $H^s(E)$ is a Hilbert space. This also implies that the canonical pairing induces an isomorphism of Hilbert spaces, $H^s(E)^*=H^{-s}(E')$, for we have $\langle \phi ,\psi \rangle =\langle (\Lambda _s^{-1})^t\phi ,\Lambda _s\psi \rangle$ and the canonical pairing induces an isomorphism of Hilbert spaces $L^2(E)^*=L^2(E')$.

The mapping properties in (d) are immediate consequences of Proposition 3.1(d), provided *r* is real. For complex *r*, we use Lemma 3.13 and Proposition 3.7(a).

The statement in (e) is now obvious, for $\Lambda _s:H^s(E)\rightarrow L^2(E)$ is a topological isomorphism with inverse induced by $\Lambda _s^{-1}:L^2(E)=H^0(E)\rightarrow H^s(E)$, see (d).

To prove (f), note that $\Lambda _{s_1}\Lambda _{s_2}'\in \Psi ^{s_1-s_2}_\text {prop}(E)$ and $s_1-s_2<0$; hence, $\Lambda _{s_1}\Lambda _{s_2}'$ induces a compact operator on $L^2_\text {loc}(E)$, see Proposition 3.7(b). Hence, the operator $\Lambda _{s_1}'\Lambda _{s_1}\Lambda _{s_2}'\Lambda _{s_2}:H^{s_2}_\text {loc}(E)\rightarrow H^{s_1}_\text {loc}(E)$ is compact. The latter operator differs from the canonical inclusion by a properly smoothing operator for we have $\Lambda _{s_1}'\Lambda _{s_1}\Lambda _{s_2}'\Lambda _{s_2}={{\,\mathrm{id}\,}}+R_{s_1}+R_{s_2}+R_{s_1}R_{s_2}$. Moreover, properly supported smoothing operators induce compact operators on each Sobolev space $H^s_\text {loc}(E)$ for the continuous inclusion $\Gamma ^\infty (E)\subseteq H^s_\text {loc}(E)$ is compact in view of the Heine–Borel property of $\Gamma ^\infty (E)$ which asserts that the identical map on $\Gamma ^\infty (E)$ is compact (Arzelà–Ascoli). We conclude that the inclusion $H^{s_2}_\text {loc}(E)\subseteq H^{s_1}_\text {loc}(E)$ is compact. Similarly, one shows that the inclusion $H^{s_2}_c(E)\subseteq H^{s_1}_c(E)$ is compact.

To prove (g), we have to show that every differential operator $D\in {\mathcal {D}}{\mathcal {O}}^r(E,F)$ of Heisenberg order at most $r<s-n/2$ induces a continuous operator $H^s_\text {loc}(E)\rightarrow \Gamma (F)$. By Proposition 3.7(c), $D\Lambda _s'\in \Psi _\text {prop}^{r-s}(E,F)$ induces a continuous operator $D\Lambda _s':L^2_\text {loc}(E)\rightarrow \Gamma (F)$ and, thus, $D\Lambda _s'\Lambda _s:H^s_\text {loc}(E)\rightarrow \Gamma (F)$ is continuous. Since the inclusion $\Gamma ^\infty (E)\subseteq \Gamma (E)$ is continuous, $DR_s\in {\mathcal {O}}^{-\infty }_\text {prop}(E,F)$ induces a continuous operator $DR_s:L^2(E)\rightarrow \Gamma (F)$. Using $D=D\Lambda _s'\Lambda _s+DR_s$, we conclude that $D:H^s_\text {loc}(E)\rightarrow \Gamma (F)$ is indeed continuous, whence the continuous inclusion $H^s_\text {loc}(E)\subseteq \Gamma ^r(E)$. Analogously, one verifies the continuous inclusion $H^s_c(E)\subseteq \Gamma _c^r(E)$, provided $r<s-n/2$. $\square$

#### Remark 3.18

For non-degenerate CR manifolds a boundedness statement as in Proposition 3.17(d) can be traced back to [33, Theorem 15.19]. For Heisenberg manifolds satisfying the bracket generating condition $H+[H,H]=TM$ a similar statement has been established in [59, Proposition 5.5.8].

#### Remark 3.19

Given $r\in {\mathbb {N}}_0$ one might wonder if the space $H^r_\text {loc}(E)$ can be characterized as the space of all $\psi \in \Gamma ^{-\infty }(E)$ such that $D\psi \in L^2_\text {loc}(F)$ for all differential operators $D\in {\mathcal {D}}{\mathcal {O}}^r(E,F)$ of Heisenberg order at most *r*; and if these differential operators suffice to generate the topology on $H^r_\text {loc}(E)$. Moreover, one might ask if $\Gamma ^r(E)$ includes (continuously) in $H^r_\text {loc}(E)$, see Remark 2.1. For general *M* and *r* neither of these properties will hold true. For instance, if $M={\mathbb {R}}\times N$ is filtered such that $TM=T^{-2}M\supseteq T^{-1}M={\mathbb {R}}\times TN$, then differential operators of Heisenberg order 1 do not suffice to characterize $H^1_\text {loc}(E)$ since they do not capture any derivative in the ${\mathbb {R}}$ direction. Filtered manifolds of this type occur naturally when studying heat operators $\partial _t+\Delta$. However, if *r* is a common multiple of $\{1,2,\dotsc ,m\}$ where *m* is such that $\mathfrak tM=\bigoplus _{j=1}^m{\mathfrak {t}}^{-j}M$, then the universal differential operator $j^r\in {\mathcal {D}}{\mathcal {O}}^r(E,J^rE)$, see Remark 2.2, is Rockland and we obtain affirmative answers to all the questions above. If $T^{-1}M$ is bracket generating, this holds true for all *r*, cf. [3, 33, 59].

This Sobolev scale permits us to refine the hypoellipticity statement in Theorem 3.11, providing a common generalization of well-known results, see, for instance, [59, Propositions 5.5.9 and 5.5.14], [33, Theorem 16.6], [44], and [3, Theorem 18.31].

#### Corollary 3.20

(Regularity and maximal hypoelliptic estimate) Let *E* and *F* be vector bundles over a filtered manifold *M*, and suppose $A\in \Psi ^k(E,F)$ satisfies the Rockland condition where *k* is some complex number. If $\psi \in \Gamma ^{-\infty }_c(E)$ and $A\psi \in H_\text {loc}^{s-\Re (k)}(F)$ for some real number *s*, then $\psi \in H^s_c(E)$. If, moreover, *M* is closed and ${\tilde{s}}\le s$, then there exists a constant $C=C_{A,s,{\tilde{s}}}\ge 0$ such that the maximal hypoelliptic estimate

$$\begin{aligned} \Vert \psi \Vert _{H^s(E)}\le C\left( \Vert \psi \Vert _{H^{{\tilde{s}}}(E)}+\Vert A\psi \Vert _{H^{s-\Re (k)}(F)}\right) \end{aligned}$$

holds for all $\psi \in H^s(E)$. Here we are using any norms generating the Hilbert space topologies on the corresponding Heisenberg Sobolev spaces. If, moreover, *Q* denotes the orthogonal projection, with respect to an inner product of the form (11), onto the (finite dimensional) subspace $\ker (A)\subseteq \Gamma ^\infty (E)$, then there exists a constant $C=C_{A,s}\ge 0$ such that the maximal hypoelliptic estimate

$$\begin{aligned} \Vert \psi \Vert _{H^s(E)}\le C\left( \Vert Q\psi \Vert _{\ker (A)}+\Vert A\psi \Vert _{H^{s-\Re (k)}(F)}\right) \end{aligned}$$

holds for all $\psi \in H^s(E)$. Here $\Vert -\Vert _{\ker (A)}$ denotes any norm on $\ker (A)$.

#### Proof

For the first part use a left parametrix as in Theorem 3.11 and Proposition 3.17(a) &(d). To see the second hypoelliptic estimate, observe that $A^*A\in \Psi ^{2\Re (k)}(E)$ satisfies the Rockland condition and $\ker (A^*A)=\ker (A)$. Hence, $B:=(A^*A+Q)^{-1}A^*\in \Psi ^{-k}(F,E)$, see Corollary 3.12, and ${{\,\mathrm{id}\,}}=Q+BA$. $\square$

#### Remark 3.21

Let us complement the regularity statement above with the following description of the topologies on the Heisenberg Sobolev spaces. Suppose $A\in \Psi ^k_\text {prop}(E,F)$ satisfies the Rockland condition and put $s=\Re (k)$. Then, the topologies on $H^s_\text {loc}(E)$ and $H^s_c(E)$ coincide with the topologies induced from the embeddings

$$\begin{aligned} H^s_\text {loc}(E)\xrightarrow {(A,{{\,\mathrm{id}\,}})}L^2_\text {loc}(F)\times \Gamma ^{-\infty }(E) \qquad \text {and}\qquad H^s_c(E)\xrightarrow {(A,{{\,\mathrm{id}\,}})}L^2_c(F)\times \Gamma ^{-\infty }_c(E), \end{aligned}$$

respectively. Moreover, if $B\in \Psi ^{-r}_\text {prop}(F,E)$ is a left parametrix such that $R:=BA-{{\,\mathrm{id}\,}}$ is a smoothing operator, see Theorem 3.11, the topologies on $H^s_\text {loc}(E)$ and $H^s_c(E)$ coincide with the topologies induced from the embeddings

$$\begin{aligned} H^s_\text {loc}(E)\xrightarrow {(A,R)}L^2_\text {loc}(F)\times \Gamma ^\infty (E) \qquad \text {and}\qquad H^s_c(E)\xrightarrow {(A,R)}L^2_c(F)\times \Gamma ^\infty _c(E), \end{aligned}$$

respectively. Indeed, $B-R$ provides a continuous left inverse for the first two inclusions; and $B-{{\,\mathrm{id}\,}}$ provides a continuous left inverse for the other two inclusions.

Accordingly, the Hodge type decomposition for formally self-adjoint Rockland operators on closed filtered manifolds in Corollary 2.9 admits the following refinement:

#### Corollary 3.22

(Hodge decomposition) Let *E* be a vector bundle over a closed filtered manifold *M*. Suppose $A\in \Psi ^k(E)$ satisfies the Rockland condition and is formally self-adjoint, $A^*=A$, with respect to an $L^2$ inner product of the form (11). Moreover, let *Q* denote the orthogonal projection onto the (finite dimensional) subspace $\ker (A)\subseteq \Gamma ^\infty (E)$. Then, for every real number *s*, we have a topological isomorphism and a Hodge type decomposition

$$\begin{aligned} A+Q:H^{s+\Re (k)}(E)\xrightarrow \cong H^s(E), \qquad H^s(E)=\ker (A)\oplus A\bigl (H^{s+\Re (k)}(E)\bigr ). \end{aligned}$$

#### Proof

This follows from Corollary 3.12 and Proposition 3.17(d). $\square$

#### Corollary 3.23

(Fredholm operators and index) Let *E* and *F* be a vector bundles over a closed filtered manifold *M*. Suppose $A\in \Psi ^k(E,F)$ is such that *A* and $A^t$ both satisfy the Rockland condition, cf. Remark 3.16. Then, for every real number *s*, we have an induced Fredholm operator $A:H^s(E)\rightarrow H^{s-\Re (k)}(F)$ whose index is independent of *s* and can be expressed as

$$\begin{aligned} {{\,\mathrm{ind}\,}}(A)=\dim \ker (A)-\dim \ker (A^t). \end{aligned}$$

Moreover, this index only depends on the Heisenberg principal cosymbol $\sigma ^k(A)\in \Sigma ^k(E,F)$.

#### Proof

According to Remark 3.15 there exists a parametrix $B\in \Psi ^{-k}(F,E)$ such that $BA-{{\,\mathrm{id}\,}}$ and $AB-{{\,\mathrm{id}\,}}$ are both smoothing operators. Since the inclusion $\Gamma ^\infty (E)\subseteq H^s(E)$ is compact, see Proposition 3.17(f), smoothing operators are compact on the Sobolev spaces $H^s(E)$ and $H^{s-\Re (k)}(F)$, respectively. Hence, $B:H^{s-\Re (k)}(F)\rightarrow H^s(E)$ provides an inverse of *A* mod compact operators. Consequently, $A:H^s(E)\rightarrow H^{s-\Re (k)}(F)$ is Fredholm. Moreover, the canonical pairing induces a canonical isomorphism ${{\,\mathrm{coker}\,}}(A)=\ker (A^t)$, see Proposition 3.17(c), whence the index formula above. By regularity, $\ker (A)$ and $\ker (A^t)$ consist of smooth sections and, thus, do not depend on *s*. If $A'\in \Psi ^k(E,F)$ is another operator with the same Heisenberg principal cosymbol, $\sigma ^k(A')=\sigma ^k(A)$, then $A'-A\in \Psi ^{k-1}(E,F)$ in view of Proposition 3.1(b); hence, $A'-A:H^s(E)\rightarrow H^{s-\Re (k)}(F)$ is a compact operator according to Proposition 3.17(d) &(f), and thus ${{\,\mathrm{ind}\,}}(A')={{\,\mathrm{ind}\,}}(A)$. $\square$

Let us now apply these results to the Rockland sequences considered in Sect. 2.3. Recall that the Rumin–Seshadri operators $\Delta _i$ satisfy the Rockland condition, see Lemma 2.14. Hence, the refined regularity statement in Corollary 3.20 applies to $\Delta _i$. Moreover, since $\Delta _i$ is formally self-adjoint, we have Hodge type decompositions and maximal hypoelliptic estimates for $\Delta _i$ as in Corollary 3.22, provided the underlying manifold is closed. For the Rockland sequences, we immediately obtain the following refinement of Corollary 2.15.

#### Corollary 3.24

(Regularity and maximal hypoelliptic estimate) In the situation of Corollary 2.15, if $\psi \in \Gamma ^{-\infty }(E_i)$ is such that $A_i\psi \in H_\text {loc}^{s-k_i}(E_{i+1})$ and $A_{i-1}^*\in H_\text {loc}^{s-k_{i-1}}(E_{i-1})$ for some real number *s*, then $\psi \in H^s_\text {loc}(E_i)$. Moreover, if *M* is closed, then there exists a constant $C=C_{A_i,s}\ge 0$ such that the maximal hypoelliptic estimate

$$\begin{aligned} \Vert \psi \Vert _{H^s(E_i)}\le C\Bigl (\Vert A_{i-1}^*\psi \Vert _{H^{s-k_{i-1}}(E_{i-1})}+\Vert Q_i\psi \Vert _{\ker (\Delta _i)}+\Vert A_i\psi \Vert _{H^{s-k_i}(E_{i+1})}\Bigr ) \end{aligned}$$

holds for all $\psi \in H^s(E_i)$. Here $\Vert -\Vert _{H^s(E_i)}$ are any norms generating the Hilbert space topology on the Heisenberg Sobolev spaces $H^s(E_i)$, $Q_i$ denotes the orthogonal projection onto the (finite dimensional) subspace $\ker (\Delta _i)=\ker (A_{i-1}^*)\cap \ker (A_i)\subseteq \Gamma ^\infty (E_i)$, and $\Vert -\Vert _{\ker (\Delta _i)}$ denotes any norm on $\ker (\Delta _i)$.

For Rockland complexes over closed manifolds we immediately obtain the following Hodge decomposition, refining the statement in Corollary 2.16:

#### Corollary 3.25

(Hodge decomposition) In the situation of Corollary 2.16 we have

$$\begin{aligned} H^s(E_i)=A_{i-1}(H^{s+k_{i-1}}(E_{i-1}))\oplus \ker (\Delta _i)\oplus A_i^*(H^{s+k_i}(E_{i+1})) \end{aligned}$$

and $\ker (A_i|_{H^s(E_i)})=A_{i-1}(H^{s+k_{i-1}}(E_{i-1}))\oplus \ker (\Delta _i)$, for every real number *s*.

#### Remark 3.26

For the ${\bar{\partial }}$ complex on a non-degenerate CR manifold the preceding statement can be found in [33, Theorem 17.1] for $s=0$.

The preceding two corollaries remain true for Rockland sequences of pseudodifferential operators. We will formulate this precisely and more generally in Sect. 5.

## Graded hypoelliptic sequences

The de Rham differentials on a filtered manifold will in general have Heisenberg order strictly larger than one, and the de Rham complex will in general not be Rockland in the sense of Definition 2.10. The de Rham complex is, however, Rockland in an appropriate graded sense. More generally, this remains true for the sequence obtained by extending a linear connection to vector bundle valued differential forms, provided the curvature satisfies an algebraic condition, see Proposition 4.15. Regarding these de Rham sequences as graded Rockland sequences, allows extracting (splitting off) graded Rockland sequences of (higher order) differential operators acting between vector bundles of smaller rank, see Theorem 4.17 and Corollary 4.18. This construction generalizes a construction for the de Rham complex on contact manifolds [65, 66] and more general C–C spaces [67, 69] due to Rumin. Since all curved Bernstein–Gelfand–Gelfand (BGG) sequences [8, 11] of regular parabolic geometries appear in this way, the latter are all graded Rockland, see Corollary 4.20. In some cases, the sequences thus obtain are even Rockland in the ungraded sense of Sect. 2.3. In particular, this happens for the BGG sequences associated with a generic rank two distribution in dimension five, see Example 4.21.

### Filtered vector bundles and differential operators

In this section, we introduce the filtration by graded Heisenberg order on differential operators acting between sections of filtered vector bundles over filtered manifolds. We discuss the corresponding graded Heisenberg principal symbol and establish some of its basic properties. For trivially filtered vector bundles these concepts reduce to the filtration by Heisenberg order and the principal Heisenberg symbol discussed in Sect. 2.1.

Let *M* be a filtered manifold. Suppose *E* is a filtered vector bundle over *M*, i.e., a smooth vector bundle which comes equipped with a filtration by smooth subbundles,

$$\begin{aligned} \cdots \supseteq E^{p-1}\supseteq E^p\supseteq E^{p+1}\supseteq \cdots . \end{aligned}$$

We will always assume that the filtration is full, that is, $E=\bigcup _pE^p$ and $\bigcap _pE^p=0$. Put ${{\,\mathrm{gr}\,}}_p(E):=E^p/E^{p+1}$ and let ${{\,\mathrm{gr}\,}}(E):=\bigoplus _p{{\,\mathrm{gr}\,}}_p(E)$ denote the associated graded vector bundle equipped with the filtration by the subbundles ${{\,\mathrm{gr}\,}}^q(E):=\bigoplus _{q\le p}{{\,\mathrm{gr}\,}}_p(E)$. By a *splitting of the filtration* on *E* we mean a filtration-preserving vector bundle isomorphism $S_E:{{\,\mathrm{gr}\,}}(E)\rightarrow E$ which induces the identity on the level of associated graded, ${{\,\mathrm{gr}\,}}(S_E)={{\,\mathrm{id}\,}}_{{{\,\mathrm{gr}\,}}(E)}$. More explicitly, $S_E$ maps ${{\,\mathrm{gr}\,}}_p(E)$ into $E^p$ such that the composition with the projection $E^p\rightarrow E^p/E^{p+1}={{\,\mathrm{gr}\,}}_p(E)$ is the identity. Such splittings always exist, in fact the space of all splittings is convex, hence contractible.

Suppose *F* is another filtered vector bundle over *M*, and let $S_F:{{\,\mathrm{gr}\,}}(F)\rightarrow F$ be a splitting for its filtration. A differential operator $A\in {\mathcal {D}}{\mathcal {O}}(E,F)$ is said to have *graded Heisenberg order* at most *k* if the operator $S_F^{-1}AS_E\in {\mathcal {D}}{\mathcal {O}}({{\,\mathrm{gr}\,}}(E),{{\,\mathrm{gr}\,}}(F))$ has the following property: The component $(S_F^{-1}AS_E)_{q,p}\in {\mathcal {D}}{\mathcal {O}}({{\,\mathrm{gr}\,}}_p(E),{{\,\mathrm{gr}\,}}_q(F))$ in the decomposition according to the gradings, $S_F^{-1}AS_E=\sum _{p,q}(S_F^{-1}AS_E)_{q,p}$, has Heisenberg order at most $k+q-p$. Consider the Heisenberg principal symbols of these components,

$$\begin{aligned} \sigma _x^{k+q-p}((S_F^{-1}AS_E)_{q,p}):C^\infty ({\mathcal {T}}_xM,{{\,\mathrm{gr}\,}}_p(E_x))\rightarrow C^\infty ({\mathcal {T}}_xM,{{\,\mathrm{gr}\,}}_q(F_x)), \end{aligned}$$

see Sect. 2.1, and define the *graded Heisenberg principal symbol*

$$\begin{aligned} {\tilde{\sigma }}_x^k(A):C^\infty ({\mathcal {T}}_xM,{{\,\mathrm{gr}\,}}(E_x))\rightarrow C^\infty ({\mathcal {T}}_xM,{{\,\mathrm{gr}\,}}(F_x)) \end{aligned}$$

by

$$\begin{aligned} {\tilde{\sigma }}^k_x(A):=\sum _{p,q}\sigma ^{k+q-p}_x\bigl ((S_F^{-1}AS_E)_{q,p}\bigr ). \end{aligned}$$

This is a left invariant differential operator which is *homogeneous of degree k in the graded sense*, that is, ${\tilde{\sigma }}^k_x(A)\circ l_g^*=l_g^*\circ {\tilde{\sigma }}_x^k(A)$ and

$$\begin{aligned} {\tilde{\sigma }}^k_x(A)\circ \delta ^{E_x}_\lambda \circ \delta _{\lambda ,x}^* =\lambda ^k\cdot \delta ^{F_x}_\lambda \circ \delta _{\lambda ,x}^*\circ {\tilde{\sigma }}^k_x(A) \end{aligned}$$

for all $g\in {\mathcal {T}}_xM$ and $\lambda >0$, see (16). Here $\delta ^{E_x}_\lambda \in {{\,\mathrm{Aut}\,}}({{\,\mathrm{gr}\,}}(E_x))$ denotes the isomorphism given by multiplication with $\lambda ^p$ on the component ${{\,\mathrm{gr}\,}}_p(E_x)$. Equivalently, the graded Heisenberg principal symbol at *x* can be considered as an element of the degree $-k$ component of ${\mathcal {U}}({\mathfrak {t}}_xM)\otimes \hom ({{\,\mathrm{gr}\,}}(E_x),{{\,\mathrm{gr}\,}}(F_x))$, that is,

$$\begin{aligned} {\tilde{\sigma }}^k_x(A)\in \bigl ({\mathcal {U}}({\mathfrak {t}}_xM)\otimes \hom ({{\,\mathrm{gr}\,}}(E_x),{{\,\mathrm{gr}\,}}(F_x))\bigr )_{-k} := \bigoplus _{p,q}{\mathcal {U}}_{-k+q-p}({\mathfrak {t}}_xM)\otimes \hom ({{\,\mathrm{gr}\,}}_p(E_x),{{\,\mathrm{gr}\,}}_q(F_x)). \end{aligned}$$

Note that any two splittings of a filtered vector bundle differ (multiplicatively) by a filtration-preserving vector bundle isomorphism inducing the identity on the associated graded. Thus, the filtration of differential operators by graded Heisenberg order does not depend on the choice of splittings $S_E$ and $S_F$, and the graded principal Heisenberg symbol is independent of this choice too. We obtain a short exact sequence

$$\begin{aligned} 0\rightarrow {\widetilde{{\mathcal {D}}{\mathcal {O}}}}^{k-1}(E,F)\rightarrow {\widetilde{{\mathcal {D}}{\mathcal {O}}}}^k(E,F)\xrightarrow {{\tilde{\sigma }}^k}\Gamma ^\infty \Bigl (\bigl ({\mathcal {U}}(\mathfrak tM)\otimes \hom ({{\,\mathrm{gr}\,}}(E),{{\,\mathrm{gr}\,}}(F))\bigr )_{-k}\Bigr )\rightarrow 0 \end{aligned}$$

where ${\widetilde{{\mathcal {D}}{\mathcal {O}}}}^k(E,F)$ denotes the space of differential operators in ${\mathcal {D}}{\mathcal {O}}(E,F)$ which are of graded Heisenberg order at most *k*.

If *G* is another filtered vector bundle, and $B\in {\mathcal {D}}{\mathcal {O}}(F,G)$ is a differential operator of graded Heisenberg order at most *l*, then the composition $BA\in {\mathcal {D}}{\mathcal {O}}(E,G)$ has graded Heisenberg order at most $l+k$ and

41 $$\begin{aligned} {\tilde{\sigma }}^{l+k}_x(BA)={\tilde{\sigma }}^l_x(B){\tilde{\sigma }}_x^k(A). \end{aligned}$$

This follows readily from (8). Moreover, the transposed operator $A^t\in {\mathcal {D}}{\mathcal {O}}(F',E')$ is of graded Heisenberg order at most *k* and

42 $$\begin{aligned} {\tilde{\sigma }}^k_x(A^t)={\tilde{\sigma }}_x^k(A)^t. \end{aligned}$$

Here the filtration on the bundle $E'=E^*\otimes |\Lambda |_M=\hom (E,|\Lambda |_M)$ is defined such that a section of $E'$ is in filtration degree *p* iff it pairs $E^q$ into the $(p+q)$-th filtration subspace of the trivially filtered line bundle $|\Lambda |_M=|\Lambda |_M^0\supseteq |\Lambda |_M^1=0$, i.e., iff it vanishes on $E^{-p+1}$. Note that the canonical isomorphism ${{\,\mathrm{gr}\,}}_p(E')=\hom (E^{-p}/E^{-p+1},|\Lambda |_M)={{\,\mathrm{gr}\,}}_{-p}(E)'$ provides a canonical isomorphism of filtered vector bundles ${{\,\mathrm{gr}\,}}(E')={{\,\mathrm{gr}\,}}(E)'$.^3^ If $S_E:{{\,\mathrm{gr}\,}}(E)\rightarrow E$ is a splitting of the filtration on *E*, then $S_E^t:E'\rightarrow {{\,\mathrm{gr}\,}}(E)'={{\,\mathrm{gr}\,}}(E')$ is filtration-preserving and $S_{E'}:=(S_E^t)^{-1}:{{\,\mathrm{gr}\,}}(E')\rightarrow E'$ is a splitting of the filtration on $E'$. Moreover, $(S_{E'}^{-1}A^tS_{F'})_{p,q}=((S_F^{-1}AS_E)_{-q,-p})^t=((S_F^{-1}AS_E)^t)_{p,q}$ and thus (42) follows at once from (8).

#### Remark 4.1

Every differential operator $A\in {\widetilde{{\mathcal {D}}{\mathcal {O}}}}^k(E,F)$ of graded Heisenberg order at most *k* maps $\Gamma ^\infty (E^p)$ into $\Gamma ^\infty (F^{p-k})$. Indeed, $(S_F^{-1}AS_E)_{q,p}=0$ for $k+q-p<0$ since there are no non-trivial differential operators of negative order. Moreover, the induced operator ${{\,\mathrm{gr}\,}}_k(A):\Gamma ^\infty ({{\,\mathrm{gr}\,}}_p(E))\rightarrow \Gamma ^\infty ({{\,\mathrm{gr}\,}}_{p-k}(F))$ is tensorial and the corresponding vector bundle homomorphism ${{\,\mathrm{gr}\,}}_k(A):{{\,\mathrm{gr}\,}}_p(E)\rightarrow {{\,\mathrm{gr}\,}}_{p-k}(F)$ coincides with the corresponding component of the graded principal Heisenberg symbol, $({\tilde{\sigma }}^k(A))_{p-k,p}$. If *A* is of graded Heisenberg order at most 0, then the associated graded will also be denoted by ${\tilde{A}}:={{\,\mathrm{gr}\,}}(A):={{\,\mathrm{gr}\,}}_0(A)$.

Generalizing Definition 2.10 we have:

#### Definition 4.2

(*Graded Rockland sequences of differential operators*) Let $E_i$ be filtered vector bundles over a filtered manifold *M*. A sequence of differential operators

$$\begin{aligned} \cdots \rightarrow \Gamma ^\infty (E_{i-1})\xrightarrow {A_{i-1}}\Gamma ^\infty (E_i)\xrightarrow {A_i}\Gamma ^\infty (E_{i+1})\rightarrow \cdots \end{aligned}$$

which are of graded Heisenberg order at most $k_i$, respectively, is said to be *graded Rockland sequence* if, for each $x\in M$ and every non-trivial irreducible unitary representation $\pi :{\mathcal {T}}_xM\rightarrow U({\mathcal {H}})$ of the osculating group ${\mathcal {T}}_xM$ the sequence

$$\begin{aligned} \cdots \rightarrow {\mathcal {H}}_\infty \otimes {{\,\mathrm{gr}\,}}(E_{i-1,x})\xrightarrow {\pi ({\tilde{\sigma }}^{k_{i-1}}_x(A_{i-1}))} {\mathcal {H}}_\infty \otimes {{\,\mathrm{gr}\,}}(E_{i,x})\xrightarrow {\pi ({\tilde{\sigma }}^{k_i}_x(A_i))} {\mathcal {H}}_\infty \otimes {{\,\mathrm{gr}\,}}(E_{i+1,x})\rightarrow \cdots \end{aligned}$$

is weakly exact, i.e., the image of each arrow is contained and dense in the kernel of the subsequent arrow. Here ${\mathcal {H}}_\infty$ denotes the subspace of smooth vectors in the Hilbert space ${\mathcal {H}}$.

### Differential projectors

In this section we consider a sequence of differential operators acting between sections of filtered vector bundles over a filtered manifold. Following the BGG machinery [8, 11, 13] we will present a construction which permits us to extract (split off) sequences of differential operators acting between sections of certain subbundles. If the original sequence was graded Rockland, then so is the new one, see Proposition 4.5(b). For the de Rham complex a similar construction can be found in [67, 69].

The construction is based on the following simple fact which will be used repeatedly below. A similar argument can be found in the construction of curved BGG sequences, see [11] or [8, Theorem 5.2], and in Rumin’s work, see [69, Lemma 2.5] or [67, Lemma 1].

#### Lemma 4.3

Let *E* and *F* be filtered vector bundles over a filtered manifold *M*. Suppose $A:\Gamma ^\infty (E)\rightarrow \Gamma ^\infty (F)$ is a differential operator of graded Heisenberg order at most zero and suppose the induced vector bundle homomorphism, ${\tilde{A}}:{{\,\mathrm{gr}\,}}(E)\rightarrow {{\,\mathrm{gr}\,}}(F)$, is invertible, cf. Remark 4.1. Then, *A* is invertible and its inverse, $A^{-1}:\Gamma ^\infty (F)\rightarrow \Gamma ^\infty (E)$, is a differential operator of graded Heisenberg order at most zero.

#### Proof

Choose splittings of the filtrations $S_E:{{\,\mathrm{gr}\,}}(E)\rightarrow E$ and $S_F:{{\,\mathrm{gr}\,}}(F)\rightarrow F$. Then, the differential operator

$$\begin{aligned} {{\,\mathrm{id}\,}}-AS_E{\tilde{A}}^{-1}S_F^{-1}:\Gamma ^\infty (F)\rightarrow \Gamma ^\infty (F) \end{aligned}$$

is nilpotent for it induces zero on the associated graded. Hence, the inverse of *A* can be expressed using the finite Neumann series,

43 $$\begin{aligned} A^{-1}=S_E{\tilde{A}}^{-1}S_F^{-1}\sum _{n=0}^\infty \Bigl ({{\,\mathrm{id}\,}}-AS_E{\tilde{A}}^{-1}S_F^{-1}\Bigr )^n. \end{aligned}$$

In view of this formula, $A^{-1}$ has graded Heisenberg order at most zero. $\square$

#### Lemma 4.4

Consider a filtered vector bundle *E* over a filtered manifold *M*. Suppose $\Box :\Gamma ^\infty (E)\rightarrow \Gamma ^\infty (E)$ is a differential operator of graded Heisenberg order at most zero, and let ${\tilde{\Box }}:{{\,\mathrm{gr}\,}}(E)\rightarrow {{\,\mathrm{gr}\,}}(E)$ denote the associated graded vector bundle endomorphism, see Remark 4.1. For each $x\in M$ let ${\tilde{P}}_x\in {{\,\mathrm{end}\,}}({{\,\mathrm{gr}\,}}(E_x))$ denote the spectral projection onto the generalized zero eigenspace of ${\tilde{\Box }}_x\in {{\,\mathrm{end}\,}}({{\,\mathrm{gr}\,}}(E_x))$. Assume that the rank of ${\tilde{P}}_x$ is locally constant in $x\in M$.

- Then, ${\tilde{P}}:{{\,\mathrm{gr}\,}}(E)\rightarrow {{\,\mathrm{gr}\,}}(E)$ is a smooth vector bundle homomorphism, ${\tilde{P}}^2={\tilde{P}}$, ${\tilde{P}}{\tilde{\Box }}={\tilde{\Box }}{\tilde{P}}$, and we obtain a decomposition of graded vector bundles,
  44 $$\begin{aligned} {{\,\mathrm{gr}\,}}(E)={{\,\mathrm{img}\,}}({\tilde{P}})\oplus \ker ({\tilde{P}}), \end{aligned}$$
  invariant under ${\tilde{\Box }}$, and such that ${\tilde{\Box }}$ is nilpotent on ${{\,\mathrm{img}\,}}({\tilde{P}})$ and invertible on $\ker ({\tilde{P}})$.
- There exists a unique filtration-preserving differential operator $P:\Gamma ^\infty (E)\rightarrow \Gamma ^\infty (E)$ such that $P^2=P$, $P\Box =\Box P$, and ${{\,\mathrm{gr}\,}}(P)={\tilde{P}}$. This operator *P* has graded Heisenberg order at most zero and provides a decomposition of filtered vector spaces,
  45 $$\begin{aligned} \Gamma ^\infty (E)={{\,\mathrm{img}\,}}(P)\oplus \ker (P), \end{aligned}$$
  which is invariant under $\Box$ and such that $\Box$ is nilpotent on ${{\,\mathrm{img}\,}}(P):=P(\Gamma ^\infty (E))$ and invertible on $\ker (P):=\{\psi \in \Gamma ^\infty (E):\Box \psi =0\}$.
- Let $S:{{\,\mathrm{gr}\,}}(E)\rightarrow E$ be a splitting of the filtration. Then
  46 $$\begin{aligned} L:\Gamma ^\infty ({{\,\mathrm{gr}\,}}(E))\rightarrow \Gamma ^\infty (E),\qquad L:=PS{\tilde{P}}+({{\,\mathrm{id}\,}}-P)S({{\,\mathrm{id}\,}}-{\tilde{P}}), \end{aligned}$$
  is an invertible differential operator of graded Heisenberg order at most zero such that ${{\,\mathrm{gr}\,}}(L)={{\,\mathrm{id}\,}}$ and $L^{-1}PL={\tilde{P}}$. Hence, *L* induces filtration-preserving isomorphisms
  $$\begin{aligned} L:\Gamma ^\infty ({{\,\mathrm{img}\,}}({\tilde{P}}))\xrightarrow \cong {{\,\mathrm{img}\,}}(P) \qquad \text {and}\qquad L:\Gamma ^\infty (\ker ({\tilde{P}}))\xrightarrow \cong \ker (P). \end{aligned}$$
  Moreover, $L^{-1}\Box L$ is a differential operator of graded Heisenberg order at most zero satisfying ${{\,\mathrm{gr}\,}}(L^{-1}\Box L)={\tilde{\Box }}$. Furthermore, $L^{-1}\Box L$ preserves the decomposition (44), its restriction to $\Gamma ^\infty ({{\,\mathrm{img}\,}}({\tilde{P}}))$ is nilpotent, and its restriction to $\Gamma ^\infty (\ker ({\tilde{P}}))$ is invertible.

#### Proof

Part (a) is well known. To prove the existence of an operator *P* as in (b) we assume, for a moment, that there exists $\varepsilon >0$ such that all non-trivial eigenvalues of ${\tilde{\Box }}_x$ lie outside the disk of radius $2\varepsilon$ centered at the origin in the complex plane for all $x\in M$. Hence, the vector bundle homomorphism $z-{\tilde{\Box }}$ is invertible, for all $0<|z|<2\varepsilon$. According to Lemma 4.3, $(z-\Box )^{-1}$ is a family of differential operators of graded Heisenberg order at most zero depending rationally on *z* for $|z|<2\varepsilon$. Hence,

47 $$\begin{aligned} P:=\frac{1}{2\pi {\mathbf {i}}}\oint _{|z|=\varepsilon }(z-\Box )^{-1}\hbox {d}z, \end{aligned}$$

defines a differential operator of graded Heisenberg order at most zero. Clearly, $P^2=P$ and $\Box P=P\Box$. Moreover, *P* is filtration-preserving and ${{\,\mathrm{gr}\,}}(P)={\tilde{P}}$ in view of ${\tilde{P}}_x=\frac{1}{2\pi {\mathbf {i}}}\oint _{|z|=\varepsilon }(z-{\tilde{\Box }}_x)^{-1}dz$. In particular, *P* gives rise to a decomposition of filtered vector spaces which is invariant under $\Box$ as indicated in (45). In general, the $\varepsilon$ used above, will only exist locally. However, since the operator in (47) does not depend on the choice of $\varepsilon$, these locally defined differential operators match up and give rise to a globally defined differential operator *P* with said properties. Using $P^2=P$ and ${\tilde{P}}^2={\tilde{P}}$, we immediately obtain $PL=L{\tilde{P}}$. In view of ${{\,\mathrm{gr}\,}}(P)={\tilde{P}}$ and ${{\,\mathrm{gr}\,}}(S)={{\,\mathrm{id}\,}}$, we have ${{\,\mathrm{gr}\,}}(L)={{\,\mathrm{id}\,}}$; hence *L* is invertible according to Lemma 4.3 and $L^{-1}$ is a differential operator of graded Heisenberg order at most zero. Since $\Box$ preserves the decomposition (45), $L^{-1}\Box L$ preserves the decomposition (44). From ${{\,\mathrm{gr}\,}}(L)={{\,\mathrm{id}\,}}$, we get ${{\,\mathrm{gr}\,}}(L^{-1}\Box L)={\tilde{\Box }}$. Using the latter it is easy to see that $\Box$ is nilpotent on ${{\,\mathrm{img}\,}}(P)$ and invertible on $\ker (P)$. Indeed, since ${\tilde{\Box }}$ is nilpotent on ${{\,\mathrm{img}\,}}({\tilde{P}})$, we conclude that $L^{-1}\Box L$ is nilpotent on $\Gamma ^\infty ({{\,\mathrm{img}\,}}({\tilde{P}}))$, and thus $\Box$ is nilpotent on ${{\,\mathrm{img}\,}}(P)$. Furthermore, since ${\tilde{\Box }}$ is invertible on $\ker ({\tilde{P}})$, Lemma 4.3 implies that $L^{-1}\Box L$ is invertible on $\Gamma ^\infty (\ker ({\tilde{P}}))$, and thus $\Box$ is invertible on $\ker (P)$. Using the latter property one readily checks the uniqueness assertion in (b). $\square$

After these preparations let us now turn to the construction of the sequences mentioned at the beginning of this section. Let $E_i$ be filtered vector bundles over a filtered manifold *M*, and consider a sequence of differential operators,

48 $$\begin{aligned} \cdots \rightarrow \Gamma ^\infty (E_{i-1})\xrightarrow {A_{i-1}}\Gamma ^\infty (E_i)\xrightarrow {A_i}\Gamma ^\infty (E_{i+1})\rightarrow \cdots , \end{aligned}$$

such that $A_i$ is of graded Heisenberg order at most $k_i$. Suppose

49 $$\begin{aligned} \cdots \leftarrow \Gamma ^\infty (E_{i-1})\xleftarrow {\delta _i}\Gamma ^\infty (E_i)\xleftarrow {\delta _{i+1}}\Gamma ^\infty (E_{i+1})\leftarrow \cdots \end{aligned}$$

is a sequence of differential operators such that $\delta _i$ is of graded Heisenberg order at most $-k_{i-1}$. Then, the differential operators

50 $$\begin{aligned} \Box _i:\Gamma ^\infty (E_i)\rightarrow \Gamma ^\infty (E_i),\qquad \Box _i:=A_{i-1}\delta _i+\delta _{i+1}A_i, \end{aligned}$$

are of graded Heisenberg order at most zero, see Sect. 4.1.^4^

We let ${\tilde{\Box }}_i:{{\,\mathrm{gr}\,}}(E_i)\rightarrow {{\,\mathrm{gr}\,}}(E_i)$, ${\tilde{\Box }}_i:={{\,\mathrm{gr}\,}}(\Box _i)$, denote the associated graded vector bundle homomorphism, see Remark 4.1. Moreover, for each $x\in M$, we let ${\tilde{P}}_{x,i}:{{\,\mathrm{gr}\,}}(E_{x,i})\rightarrow {{\,\mathrm{gr}\,}}(E_{x,i})$ denote the spectral projection onto the generalized zero eigenspace of ${\tilde{\Box }}_{x,i}\in {{\,\mathrm{end}\,}}({{\,\mathrm{gr}\,}}(E_{x,i}))$. Note that the projectors ${\tilde{P}}_{x,i}$ preserve the grading on ${{\,\mathrm{gr}\,}}(E_{x,i})$. We assume that the rank of ${\tilde{P}}_{x,i}$ is locally constant in *x*. Consequently, these fiber-wise projectors provide a smooth vector bundle projector ${\tilde{P}}_i:{{\,\mathrm{gr}\,}}(E_i)\rightarrow {{\,\mathrm{gr}\,}}(E_i)$ and we obtain a decomposition of graded vector bundles,

51 $$\begin{aligned} {{\,\mathrm{gr}\,}}(E_i)={{\,\mathrm{img}\,}}({\tilde{P}}_i)\oplus \ker ({\tilde{P}}_i), \end{aligned}$$

which is invariant under ${\tilde{\Box }}_i$, see Lemma 4.4(a). By construction, ${\tilde{\Box }}_i$ is nilpotent on ${{\,\mathrm{img}\,}}({\tilde{P}}_i)$ and invertible on $\ker ({\tilde{P}}_i)$. We let ${\tilde{A}}_i:{{\,\mathrm{gr}\,}}_*(E_i)\rightarrow {{\,\mathrm{gr}\,}}_{*-k_i}(E_{i+1})$ and ${\tilde{\delta }}_i:{{\,\mathrm{gr}\,}}_*(E_i)\rightarrow {{\,\mathrm{gr}\,}}_{*+k_{i-1}}(E_{i-1})$ denote the associated graded vector bundle homomorphisms of $A_i$ and $\delta _i$, respectively, see Remark 4.1. Clearly, ${\tilde{\Box }}_i={\tilde{A}}_{i-1}{\tilde{\delta }}_i+{\tilde{\delta }}_{i+1}{\tilde{A}}_i$, see (50). If ${\tilde{A}}_i{\tilde{A}}_{i-1}=0$ for all *i*, then ${\tilde{\Box }}_{i+1}{\tilde{A}}_i={\tilde{A}}_i{\tilde{\Box }}_i$, ${\tilde{P}}_{i+1}{\tilde{A}}_i={\tilde{A}}_i{\tilde{P}}_i$, and, thus, ${\tilde{A}}_i$ preserves the decompositions (51). Similarly, if ${\tilde{\delta }}_{i-1}{\tilde{\delta }}_i=0$ for all *i*, then ${\tilde{\Box }}_{i-1}{\tilde{\delta }}_i={\tilde{\delta }}_i{\tilde{\Box }}_i$, ${\tilde{P}}_{i-1}{\tilde{\delta }}_i={\tilde{\delta }}_i{\tilde{P}}_i$, and ${\tilde{\delta }}_i$ preserves the decompositions in (51).

According to Lemma 4.4(b), there exists a unique filtration-preserving differential operator

$$\begin{aligned} P_i:\Gamma ^\infty (E_i)\rightarrow \Gamma ^\infty (E_i) \end{aligned}$$

such that $P_i^2=P_i$, $P_i\Box _i=\Box _iP_i$, and ${{\,\mathrm{gr}\,}}(P_i)={\tilde{P}}_i$. These projectors are of graded Heisenberg order at most zero and provide a decomposition $\Gamma ^\infty (E_i)={{\,\mathrm{img}\,}}(P_i)\oplus \ker (P_i)$ such that $\Box _i$ is nilpotent on ${{\,\mathrm{img}\,}}(P_i)$ and invertible on $\ker (P_i)$.

We fix splittings for the filtrations, $S_i:{{\,\mathrm{gr}\,}}(E_i)\rightarrow E_i$, and consider the differential operators

$$\begin{aligned} L_i:\Gamma ^\infty ({{\,\mathrm{gr}\,}}(E_i))\rightarrow \Gamma ^\infty (E_i),\qquad L_i:=P_iS_i{\tilde{P}}_i+({{\,\mathrm{id}\,}}-P_i)S_i({{\,\mathrm{id}\,}}-{\tilde{P}}_i). \end{aligned}$$

In view of Lemma 4.4(c), $L_i$ is an invertible differential operator of graded Heisenberg order at most zero such that ${{\,\mathrm{gr}\,}}(L_i)={{\,\mathrm{id}\,}}$ and $L_i^{-1}P_iL_i={\tilde{P}}_i$. Moreover, $L^{-1}_i\Box _iL_i$ is a differential operator of graded Heisenberg order at most zero such that ${{\,\mathrm{gr}\,}}(L_i^{-1}\Box _iL_i)={\tilde{\Box }}_i$. Furthermore, $L^{-1}_i\Box _iL_i$ preserves the decomposition (51), its restriction to $\Gamma ^\infty ({{\,\mathrm{img}\,}}({\tilde{P}}_i))$ is nilpotent and its restriction to $\Gamma ^\infty (\ker ({\tilde{P}}_i))$ is invertible.

Conjugating the original sequence (48) by $L_i$, we obtain two sequences,

52 $$\begin{aligned} \cdots \rightarrow \Gamma ^\infty ({{\,\mathrm{img}\,}}({\tilde{P}}_{i-1}))\xrightarrow {D_{i-1}}\Gamma ^\infty ({{\,\mathrm{img}\,}}({\tilde{P}}_i))\xrightarrow {D_i}\Gamma ^\infty ({{\,\mathrm{img}\,}}({\tilde{P}}_{i+1}))\rightarrow \cdots \end{aligned}$$

and

53 $$\begin{aligned} \cdots \rightarrow \Gamma ^\infty (\ker ({\tilde{P}}_{i-1}))\xrightarrow {B_{i-1}}\Gamma ^\infty (\ker ({\tilde{P}}_i))\xrightarrow {B_i}\Gamma ^\infty (\ker ({\tilde{P}}_{i+1}))\rightarrow \cdots , \end{aligned}$$

where

54 $$\begin{aligned} D_i:={\tilde{P}}_{i+1}L_{i+1}^{-1}A_iL_i|_{\Gamma ^\infty ({{\,\mathrm{img}\,}}({\tilde{P}}_i))} \quad \text {and}\quad B_i:=({{\,\mathrm{id}\,}}-{\tilde{P}}_{i+1})L_{i+1}^{-1}A_iL_i|_{\Gamma ^\infty (\ker ({\tilde{P}}_i))} \end{aligned}$$

are differential operators of graded Heisenberg order at most $k_i$. This generalizes a construction for the de Rham complex due to Rumin, see [69, Theorem 2.6] and [67, Theorem 1].

#### Proposition 4.5

In this situation, the following hold true:

- If ${\tilde{\sigma }}^{k_i}_x(A_i){\tilde{\sigma }}^{k_{i-1}}_x(A_{i-1})=0$ for all *i* and *x*, then
  $$\begin{aligned} {\tilde{\sigma }}^{k_i}_x(L^{-1}_{i+1}A_iL_i)={\tilde{\sigma }}^{k_i}_x(D_i)\oplus {\tilde{\sigma }}^{k_i}_x(B_i). \end{aligned}$$
- If the sequence (48) is graded Rockland, then so are the sequences (52) and (53).
- If $A_iA_{i-1}=0$ for all *i*, then the operator $L_{i+1}^{-1}A_iL_i$ decouples,
  $$\begin{aligned} L^{-1}_{i+1}A_iL_i=D_i\oplus B_i, \end{aligned}$$
  and we have $D_iD_{i-1}=0$, $B_iB_{i-1}=0$. In this situation, $G_i:\Gamma ^\infty (\ker ({\tilde{P}}_i))\rightarrow \Gamma ^\infty (\ker ({\tilde{P}}_i))$,
  $$\begin{aligned} G_i:=B_{i-1}({{\,\mathrm{id}\,}}-{\tilde{P}}_{i-1}){\tilde{\delta }}_i{\tilde{\Box }}_i^{-1} +({{\,\mathrm{id}\,}}-{\tilde{P}}_i){\tilde{\delta }}_{i+1}{\tilde{\Box }}_{i+1}^{-1}{\tilde{A}}_i, \end{aligned}$$
  is an invertible differential operator of graded Heisenberg order at most zero with ${{\,\mathrm{gr}\,}}(G_i)={{\,\mathrm{id}\,}}$ that conjugates the complex (53) into an acyclic tensorial complex, namely,
  $$\begin{aligned} G_{i+1}^{-1}B_iG_i={\tilde{A}}_i|_{\Gamma ^\infty (\ker ({\tilde{P}}_i))}. \end{aligned}$$
  Moreover, the restriction $L_i:\Gamma ^\infty ({{\,\mathrm{img}\,}}({\tilde{P}}_i))\rightarrow \Gamma ^\infty (E_i)$ provides a chain map, that is, $A_iL_i|_{\Gamma ^\infty ({{\,\mathrm{img}\,}}({\tilde{P}}_i))}=L_{i+1}D_i$, which induces an isomorphism between the cohomologies of (52) and (48). More precisely, $\pi _i:={\tilde{P}}_iL_i^{-1}:\Gamma ^\infty (E_i)\rightarrow \Gamma ^\infty ({{\,\mathrm{img}\,}}({\tilde{P}}_i))$, is a chain map, $D_i\pi _i=\pi _{i+1}A_i$, which is an inverse of $L_i$ up to homotopy, i.e., $\pi _iL_i|_{\Gamma ^\infty ({{\,\mathrm{img}\,}}({\tilde{P}}_i))}={{\,\mathrm{id}\,}}$ and ${{\,\mathrm{id}\,}}-L_i\pi _i=A_{i-1}h_i+h_{i+1}A_i$ where $h_i:\Gamma ^\infty (E_i)\rightarrow \Gamma ^\infty (E_{i-1})$ is a differential operator of graded Heisenberg order at most $-k_{i-1}$ given by $h_i:=L_{i-1}G_{i-1}({{\,\mathrm{id}\,}}-{\tilde{P}}_{i-1}){\tilde{\delta }}_i{\tilde{\Box }}_i^{-1}G_i^{-1}({{\,\mathrm{id}\,}}-{\tilde{P}}_i)L_i^{-1}$.

#### Proof

To show part (c) suppose $A_iA_{i-1}=0$ for all *i*. Then, $\Box _{i+1}A_i=A_i\Box _i$, see (50), and thus $P_{i+1}A_i=A_iP_i$, see (47). Using the relation $L_i^{-1}P_iL_i={\tilde{P}}_i$ from Lemma 4.4(c), we obtain ${\tilde{P}}_{i+1}(L_{i+1}^{-1}A_iL_i)=(L_{i+1}^{-1}A_iL_i){\tilde{P}}_i$, hence ${\tilde{P}}_{i+1}(L_{i+1}^{-1}A_iL_i)({{\,\mathrm{id}\,}}-{\tilde{P}}_i)=0=({{\,\mathrm{id}\,}}-{\tilde{P}}_{i+1})(L_{i+1}^{-1}A_iL_i){\tilde{P}}_i$,

$$\begin{aligned} L^{-1}_{i+1}A_iL_i={\tilde{P}}_{i+1}(L^{-1}_{i+1}A_iL_i){\tilde{P}}_i +({{\,\mathrm{id}\,}}-{\tilde{P}}_{i+1})(L^{-1}_{i+1}A_iL_i)({{\,\mathrm{id}\,}}-{\tilde{P}}_i), \end{aligned}$$

and thus $L^{-1}_{i+1}A_iL=D_i\oplus B_i$, cf. (54). Clearly, $A_iA_{i-1}=0$ implies $D_iD_{i-1}=0$, $B_iB_{i-1}=0$, and $B_iG_i=G_{i+1}{\tilde{A}}_i$. Clearly, ${{\,\mathrm{gr}\,}}(G_i)={{\,\mathrm{id}\,}}$ and, thus, $G_i$ is invertible according to Lemma 4.3. The remaining assertions in (c) are now straight forward.

To show part (a) suppose ${\tilde{\sigma }}^{k_i}_x(A_i){\tilde{\sigma }}^{k_{i-1}}_x(A_{i-1})=0$ for all *i* and all $x\in M$. Using the multiplicativity of the graded Heisenberg principal symbol, see (41), we conclude ${\tilde{\sigma }}^0_x(\Box _{i+1}){\tilde{\sigma }}^{k_i}_x(A_i)={\tilde{\sigma }}_x^{k_i}(A_i){\tilde{\sigma }}^0_x(\Box _i)$, see (50), and thus ${\tilde{\sigma }}^0_x(P_{i+1}){\tilde{\sigma }}^{k_i}_x(A_i)={\tilde{\sigma }}^{k_i}_x(A_i){\tilde{\sigma }}^0_x(P_i)$, see (47). Combining this with $L_i^{-1}P_iL_i={\tilde{P}}_i$ from Lemma 4.4(c), we obtain ${\tilde{\sigma }}_x^{k_i}\bigl ({\tilde{P}}_{i+1}(L_{i+1}^{-1}A_iL_i)\bigr )={\tilde{\sigma }}^{k_i}_x\bigl ((L_{i+1}^{-1}A_iL_i){\tilde{P}}_i\bigr )$, hence ${\tilde{\sigma }}^{k_i}_x\bigl ({\tilde{P}}_{i+1}(L_{i+1}^{-1}A_iL_i)({{\,\mathrm{id}\,}}-{\tilde{P}}_i)\bigr )=0={\tilde{\sigma }}^{k_i}_x\bigl (({{\,\mathrm{id}\,}}-{\tilde{P}}_{i+1})(L_{i+1}^{-1}A_iL_i){\tilde{P}}_i\bigr )$,

$$\begin{aligned} {\tilde{\sigma }}^{k_i}_x(L^{-1}_{i+1}A_iL_i) ={\tilde{\sigma }}^{k_i}_x\bigl ({\tilde{P}}_{i+1}(L^{-1}_{i+1}A_iL_i){\tilde{P}}_i\bigr ) +{\tilde{\sigma }}^{k_i}_x\bigl (({{\,\mathrm{id}\,}}-{\tilde{P}}_{i+1})(L^{-1}_{i+1}A_iL_i)({{\,\mathrm{id}\,}}-{\tilde{P}}_i)\bigr ), \end{aligned}$$

and thus $${\tilde{\sigma }}^{k_i}_x(L^{-1}_{i+1}A_iL_i)={\tilde{\sigma }}^{k_i}_x(D_i)\oplus {\tilde{\sigma }}^{k_i}_x(B_i)$$.

To see (b) suppose the sequence (48) is graded Rockland, that is, the graded Heisenberg principal symbol sequences $\sigma ^{k_i}_x(A_i)$ are weakly exact in every non-trivial irreducible unitary representation of ${\mathcal {T}}_xM$. Clearly, the conjugated sequence ${\tilde{\sigma }}^{k_i}_x(L_{i+1}^{-1}A_iL_i)={\tilde{\sigma }}^0_x(L_{i+1})^{-1}{\tilde{\sigma }}^{k_i}_x(A_i){\tilde{\sigma }}^0_x(L_i)$ has the same property. Note that ${\tilde{\sigma }}^{k_i}_x(A_i){\tilde{\sigma }}^{k_{i-1}}_x(A_{i-1})=0$ since this relation holds true in every non-trivial irreducible unitary representation of ${\mathcal {T}}_xM$. Hence, part (a), permits us to conclude that the graded Heisenberg principal symbol sequences ${\tilde{\sigma }}^{k_i}_x(D_i)$ and ${\tilde{\sigma }}^{k_i}_x(B_i)$ are weakly exact in every non-trivial irreducible unitary representation of ${\mathcal {T}}_xM$ too. $\square$

### Splitting operators

The sequences (52) and (53) constructed above depend on the operators $A_i$ and $\delta _i$, see (48) and (49) but also on the splittings $S_i:{{\,\mathrm{gr}\,}}(E_i)\rightarrow E_i$. We will now specialize to a situation in which one can construct a variant of the sequence (52) which does not depend on the splittings $S_i$, but only on $A_i$ and $\delta _i$. The sequences obtain in this way, see Proposition 4.8, generalize the curved BGG sequences [8, 11] discussed in Sect. 4.6.

We continue to use the notation from the preceding sections. In particular, $E_i$ are filtered vector bundles over a filtered manifold *M*, and we consider a sequence,

55 $$\begin{aligned} \cdots \rightarrow \Gamma ^\infty (E_{i-1})\xrightarrow {A_{i-1}}\Gamma ^\infty (E_i)\xrightarrow {A_i}\Gamma ^\infty (E_{i+1})\rightarrow \cdots , \end{aligned}$$

where $A_i$ is a differential operator of graded Heisenberg order at most $k_i$.

#### Definition 4.6

(*Codifferential of Kostant type*) A sequence of vector bundle homomorphisms,

56 $$\begin{aligned} \cdots \leftarrow E_{i-1}\xleftarrow {\delta _i}E_i\xleftarrow {\delta _{i+1}}E_{i+1}\leftarrow \cdots , \end{aligned}$$

will be called a *codifferential of Kostant type* for the sequence (55) if it has the following properties:

(i) $$\delta _i\delta _{i+1}=0$$ for all *i*.
(ii) $\delta _i$ maps the filtration space $E_i^p$ into $E_{i-1}^{p+k_{i-1}}$.^5^
(iii) There exist splittings of the filtrations, $S_i:{{\,\mathrm{gr}\,}}(E_i)\rightarrow E_i$, such that ${\tilde{\delta }}_i=S_{i-1}^{-1}\delta _iS_i$.^6^
(iv) ${\tilde{\delta }}_{i,x}{\tilde{P}}_{i,x}=0$ for each $x\in M$.

Here ${\tilde{A}}_i:{{\,\mathrm{gr}\,}}_*(E_i)\rightarrow {{\,\mathrm{gr}\,}}_{*-k_i}(E_{i+1})$, ${\tilde{\delta }}_i:{{\,\mathrm{gr}\,}}_*(E_i)\rightarrow {{\,\mathrm{gr}\,}}_{*+k_{i-1}}(E_{i-1})$, and ${\tilde{\Box }}_i:{{\,\mathrm{gr}\,}}_*(E_i)\rightarrow {{\,\mathrm{gr}\,}}_*(E_i)$ denote the vector bundle homomorphisms induced by $A_i$, $\delta _i$ and $\Box _i=A_{i-1}\delta _i+\delta _{i+1}A_i$ on the associated graded vector bundles, respectively, see Remark 4.1. Moreover, ${\tilde{P}}_{i,x}:E_{i,x}\rightarrow E_{i,x}$ denotes the spectral projection onto the generalized zero eigenspace of ${\tilde{\Box }}_{i,x}$.

#### Lemma 4.7

Consider a sequence of vector bundle homomorphisms $\delta _i$ as in (56) which is a codifferential of Kostant type for the sequence (55), see Definition 4.6, and assume that the rank of $\delta _{x,i}$ is locally constant in *x*, for each *i*. Then, the following hold true:

- The rank of ${\tilde{P}}_{i,x}$ is locally constant in *x*, these families provide smooth vector bundle projectors, ${\tilde{P}}_i:{{\,\mathrm{gr}\,}}(E_i)\rightarrow {{\,\mathrm{gr}\,}}(E_i)$, such that ${\tilde{\delta }}_i{\tilde{\delta }}_{i+1}=0$, ${\tilde{\Box }}_{i-1}{\tilde{\delta }}_i={\tilde{\delta }}_i{\tilde{\Box }}_i$, ${\tilde{P}}_i{\tilde{\Box }}_i={\tilde{\Box }}_i{\tilde{P}}_i$, ${\tilde{P}}_{i-1}{\tilde{\delta }}_i={\tilde{\delta }}_i{\tilde{P}}_i=0$, and we have a decomposition of graded vector bundles,
  57 $$\begin{aligned} \ker ({\tilde{\delta }}_i)={{\,\mathrm{img}\,}}({\tilde{P}}_i)\oplus {{\,\mathrm{img}\,}}({\tilde{\delta }}_{i+1}). \end{aligned}$$
- Let $P_i:\Gamma ^\infty (E_i)\rightarrow \Gamma ^\infty (E_i)$ denote the unique filtration-preserving differential operator such that $P_i^2=P_i$, $P_i\Box _i=\Box _iP_i$ and ${{\,\mathrm{gr}\,}}(P_i)={\tilde{P}}_i$, see Lemma 4.4(b). Then, $\Box _{i-1}\delta _i=\delta _i\Box _i$, $P_{i-1}\delta _i=\delta _iP_i=0$, and we obtain a decomposition of filtered spaces,
  58 $$\begin{aligned} \Gamma ^\infty (\ker (\delta _i))={{\,\mathrm{img}\,}}(P_i)\oplus \Gamma ^\infty ({{\,\mathrm{img}\,}}(\delta _{i+1})). \end{aligned}$$
  Moreover, the quotient bundle ${\mathcal {H}}_i:=\ker (\delta _i)/{{\,\mathrm{img}\,}}(\delta _{i+1})$ is a filtered vector bundle and $P_i$ factors to a differential operator of graded Heisenberg order at most zero,
  $$\begin{aligned} {\bar{L}}_i:\Gamma ^\infty ({\mathcal {H}}_i)\xrightarrow \cong {{\,\mathrm{img}\,}}(P_i)\subseteq \Gamma ^\infty (E_i), \end{aligned}$$
  which is inverse to the restriction of the canonical projection ${\bar{\pi }}_i:\ker (\delta _i)\rightarrow {\mathcal {H}}_i$, that is, ${\bar{\pi }}_i{\bar{L}}_i={{\,\mathrm{id}\,}}$ and ${\bar{L}}_i{\bar{\pi }}_i=P_i$.
- Assume, moreover, ${\tilde{A}}_{i+1}{\tilde{A}}_i=0$ for all *i*. Then ${\tilde{\Box }}_{i+1}{\tilde{A}}_i={\tilde{A}}_i{\tilde{\Box }}_i$, ${\tilde{P}}_{i+1}{\tilde{A}}_i={\tilde{A}}_i{\tilde{P}}_i$, ${\tilde{\delta }}_{i+1}{\tilde{A}}_i{\tilde{P}}_i=0$, and
  59 $$\begin{aligned} {{\,\mathrm{img}\,}}({\tilde{P}}_i)=\ker ({\tilde{\Box }}_i) =\ker ({\tilde{\delta }}_i)\cap \ker ({\tilde{\delta }}_{i+1}{\tilde{A}}_i). \end{aligned}$$
  Moreover, $$\delta _{i+1}A_iP_i=0$$, and
  60 $$\begin{aligned} {{\,\mathrm{img}\,}}(P_i)=\ker (\Box _i)=\ker (\delta _i)\cap \ker (\delta _{i+1}A_i). \end{aligned}$$

#### Proof

In view of ${\tilde{\delta }}_{i-1,x}{\tilde{\delta }}_{i,x}=0$ and ${\tilde{\delta }}_{i,x}{\tilde{P}}_{i,x}=0$ we also have ${\tilde{\Box }}_{i-1,x}{\tilde{\delta }}_{i,x}={\tilde{\delta }}_{i,x}{\tilde{\Box }}_{i,x}$ and ${\tilde{P}}_{i-1,x}{\tilde{\delta }}_{i,x}={\tilde{\delta }}_{i,x}{\tilde{P}}_{i,x}=0$. Moreover, since ${\tilde{A}}_{i-1,x}{\tilde{\delta }}_{i,x}+{\tilde{\delta }}_{i+1,x}{\tilde{A}}_{i,x}={\tilde{\Box }}_{i,x}$ is invertible on $\ker ({\tilde{P}}_{i,x})$, it is straight forward to establish the finite dimensional decomposition $\ker ({\tilde{\delta }}_{i,x})={{\,\mathrm{img}\,}}({\tilde{P}}_{i,x})\oplus {{\,\mathrm{img}\,}}({\tilde{\delta }}_{i+1,x})$ for each $x\in M$. Since ${\tilde{\delta }}_{i,x}$ is assumed to have locally constant rank, the same must be true for ${\tilde{P}}_{i,x}$. Hence, ${\tilde{P}}_i$ is a smooth vector bundle homomorphism, and (57) is a decomposition of smooth vector bundles. The remaining assertions in (a) are obvious.

Clearly, $\delta _i\Box _i=\Box _{i-1}\delta _i$ and $\delta _iP_i=P_{i-1}\delta _i$, see (50) and (47). To show $\delta _iP_i=0$, we consider the operator

61 $$\begin{aligned} L_i:\Gamma ^\infty ({{\,\mathrm{gr}\,}}(E_i))\rightarrow \Gamma ^\infty (E_i),\qquad L_i=P_iS_i{\tilde{P}}_i+({{\,\mathrm{id}\,}}-P_i)S_i({{\,\mathrm{id}\,}}-{\tilde{P}}_i), \end{aligned}$$

cf. (46) in Lemma 4.4, where the splittings $S_i$ are as in Definition 4.6(iii). Then $L_{i-1}^{-1}\delta _iL_i={\tilde{\delta }}_i$ and $L^{-1}_iP_iL_i={\tilde{P}}_i$. The assertion $\delta _iP_i=0$ thus follows from ${\tilde{\delta }}_i{\tilde{P}}_i=0$, see Definition 4.6(iv). Since $A_{i-1}\delta _i+\delta _{i+1}A_i=\Box _i$ is invertible on $\ker (P_i)$, it is now straight forward to establish the decomposition (58). Clearly, ${{\,\mathrm{img}\,}}(\delta _{i+1})$, $\ker (\delta _i)$ and ${\mathcal {H}}_i=\ker (\delta _i)/{{\,\mathrm{img}\,}}(\delta _{i+1})$ are smooth vector bundles. Moreover, $P_i$ factorizes to a linear map ${\bar{L}}_i:\Gamma ^\infty ({\mathcal {H}}_i)\rightarrow {{\,\mathrm{img}\,}}(P_i)$ such that ${\bar{L}}_i{\bar{\pi }}_i=P_i$ and ${\bar{\pi }}_i{\bar{L}}_i={{\,\mathrm{id}\,}}$. To see that ${\bar{L}}_i$ is a differential operator of graded Heisenberg order at most zero, it suffices to observe that ${\bar{L}}_i$ can be written (locally) as the composition of $P_i$ with a filtration-preserving vector bundle homomorphism ${\mathcal {H}}_i\rightarrow \ker (\delta _i)$.

If ${\tilde{A}}_i{\tilde{A}}_{i-1}=0$, then the assertions ${\tilde{\Box }}_{i+1}{\tilde{A}}_i={\tilde{A}}_i{\tilde{\Box }}_i$, ${\tilde{P}}_{i+1}{\tilde{A}}_i={\tilde{A}}_i{\tilde{P}}_i$, and ${\tilde{\delta }}_{i+1}{\tilde{A}}_i{\tilde{P}}_i=0$, as well as the equalities in (59) are now obvious. Using $\Box _i=\delta _{i+1}A_i+A_{i-1}\delta _i$, $\delta _iP_i=0=P_{i-1}\delta _i$, $\Box _iP_i=P_i\Box _i$ and $P^2_i=P_i$, we obtain $\delta _{i+1}A_iP_i=\Box _iP_i=P_i\Box _iP_i=0$, whence the equalities in (60). The remaining assertions follow at once. $\square$

#### Proposition 4.8

Let $E_i$ be filtered vector bundles over a filtered manifold *M*, and consider a sequence of differential operators,

62 $$\begin{aligned} \cdots \rightarrow \Gamma ^\infty (E_{i-1})\xrightarrow {A_{i-1}}\Gamma ^\infty (E_i)\xrightarrow {A_i}\Gamma ^\infty (E_{i+1})\rightarrow \cdots , \end{aligned}$$

such that $A_i$ is of graded Heisenberg order at most $k_i$ and ${\tilde{A}}_i{\tilde{A}}_{i-1}=0$ for all *i*. Moreover, let

$$\begin{aligned} \cdots \leftarrow E_{i-1}\xleftarrow {\delta _i}E_i\xleftarrow {\delta _{i+1}}E_{i+1}\leftarrow \cdots \end{aligned}$$

be a codifferential of Kostant type for the sequence (62), see Definition 4.6. Assume that each $\delta _i$ has locally constant rank, and let ${\bar{\pi }}_i:\ker (\delta _i)\rightarrow {\mathcal {H}}_i:=\ker (\delta _i)/{{\,\mathrm{img}\,}}(\delta _{i+1})$ denote the canonical vector bundle projection. Then, the following hold true:

- There exists a unique differential operator ${\bar{L}}_i:\Gamma ^\infty ({\mathcal {H}}_i)\rightarrow \Gamma ^\infty (E_i)$ such that $\delta _i{\bar{L}}_i=0$, ${\bar{\pi }}_i{\bar{L}}_i={{\,\mathrm{id}\,}}$, and $\delta _{i+1}A_i{\bar{L}}_i=0$. Moreover, ${\bar{L}}_i$ is of graded Heisenberg order at most zero, and the differential operator
  63 $$\begin{aligned} {\bar{D}}_i:\Gamma ^\infty ({\mathcal {H}}_i)\rightarrow \Gamma ^\infty ({\mathcal {H}}_{i+1}),\qquad {\bar{D}}_i:={\bar{\pi }}_{i+1}A_i{\bar{L}}_i, \end{aligned}$$
  is of graded Heisenberg order at most $k_i$.
- If the sequence (62) is graded Rockland, then so is the sequence:
  64 $$\begin{aligned} \cdots \rightarrow \Gamma ^\infty ({\mathcal {H}}_{i-1})\xrightarrow {{\bar{D}}_{i-1}}\Gamma ^\infty ({\mathcal {H}}_i)\xrightarrow {{\bar{D}}_i}\Gamma ^\infty ({\mathcal {H}}_{i+1})\rightarrow \cdots . \end{aligned}$$
- If $A_iA_{i-1}=0$, then ${\bar{D}}_i{\bar{D}}_{i-1}=0$, and ${\bar{L}}_i:\Gamma ^\infty ({\mathcal {H}}_i)\rightarrow \Gamma ^\infty (E_i)$ is a chain map, $A_i{\bar{L}}_i={\bar{L}}_{i+1}{\bar{D}}_i$, inducing an isomorphism between the cohomologies of (64) and (62).
- There exist invertible differential operators, $V_i:\Gamma ^\infty ({{\,\mathrm{img}\,}}({\tilde{P}}_i))\rightarrow \Gamma ^\infty ({\mathcal {H}}_i)$ with inverse $V_i^{-1}:\Gamma ^\infty ({\mathcal {H}}_i)\rightarrow \Gamma ^\infty ({{\,\mathrm{img}\,}}({\tilde{P}}_i))$, both of graded Heisenberg order at most zero, such that
  $$\begin{aligned} V_{i+1}^{-1}{\bar{D}}_iV_i=D_i, \end{aligned}$$
  where $D_i:\Gamma ^\infty ({{\,\mathrm{img}\,}}({\tilde{P}}_i))\rightarrow \Gamma ^\infty ({{\,\mathrm{img}\,}}({\tilde{P}}_{i+1}))$ denotes the operator considered in Sect. 4.2, see (54), associated with splittings $S_i$ as in Definition 4.6(iii).

#### Proof

Part (a) follows immediately from Lemma 4.7(b) &(c). Recall the differential operator $L_i$ associated with the splittings $S_i$, see (61). The differential operator $V_i:\Gamma ^\infty ({{\,\mathrm{img}\,}}({\tilde{P}}_i))\rightarrow \Gamma ^\infty ({\mathcal {H}}_i)$, $V_i:={\bar{\pi }}_iL_i|_{{{\,\mathrm{img}\,}}({\tilde{P}}_i)}$ has graded Heisenberg order at most zero and so does its inverse, $V_i^{-1}:\Gamma ^\infty ({\mathcal {H}}_i)\rightarrow \Gamma ^\infty ({{\,\mathrm{img}\,}}({\tilde{P}}_i))$, $V_i^{-1}=L_i^{-1}{\bar{L}}_i$. Clearly, $V_{i+1}^{-1}{\bar{D}}_iV_i=D_i$, whence (d). Combining this with Proposition 4.5(b) &(c), we obtain the statements in (b) in (c). $\square$

The operators ${\bar{L}}_i$ in Proposition 4.8(a) are direct generalizations of the well-known splitting operators in parabolic geometry, see [12, Theorem 2.4]. The operators ${\bar{D}}_i$ generalize the BGG operators.

#### Remark 4.9

If the filtration on each ${\mathcal {H}}_i$ is trivial, then (64) is a Rockland sequence in the ungraded sense of Definition 2.10.

#### Remark 4.10

For the Kostant type codifferential $\delta _i=0$ all statements in Proposition 4.8 are trivially true, ${\bar{\pi }}_i={{\,\mathrm{id}\,}}={\bar{L}}_i$.

#### Remark 4.11

If ${\tilde{A}}_i{\tilde{A}}_{i-1}=0$, then the rank of a Kostant type codifferential is bounded by

65 $$\begin{aligned} \sum _i{{\,\mathrm{rank}\,}}(\delta _{i,x})\le \sum _i{{\,\mathrm{rank}\,}}({\tilde{A}}_{i,x}). \end{aligned}$$

Indeed, from ${\tilde{\delta }}_i{\tilde{\delta }}_{i+1}=0$ and ${\tilde{\delta }}_i{\tilde{P}}_i=0$ we get $\ker ({\tilde{\delta }}_{i,x})={{\,\mathrm{img}\,}}({\tilde{P}}_{i,x})\oplus {{\,\mathrm{img}\,}}({\tilde{\delta }}_{i+1,x})$ and, consequently, $\ker (\delta _{i,x})/{{\,\mathrm{img}\,}}(\delta _{i+1,x})\cong \ker ({\tilde{\delta }}_{i,x})/{{\,\mathrm{img}\,}}({\tilde{\delta }}_{i+1,x})\cong {{\,\mathrm{img}\,}}({\tilde{P}}_{i,x})$. Moreover, ${\tilde{A}}_{i,x}$ preserves the decomposition ${{\,\mathrm{gr}\,}}(E_i)={{\,\mathrm{img}\,}}({\tilde{P}}_{i,x})\oplus \ker ({\tilde{P}}_{i,x})$ and its restriction to $\ker ({\tilde{P}}_{i,x})$ is acyclic since ${\tilde{\Box }}_{i,x}$ is invertible on $\ker ({\tilde{P}}_{i,x})$. We conclude

66 $$\begin{aligned} \dim \bigl (\ker ({\tilde{A}}_{i,x})/{{\,\mathrm{img}\,}}({\tilde{A}}_{i-1,x})\bigr ) \le \dim \bigl (\ker (\delta _{i,x})/{{\,\mathrm{img}\,}}(\delta _{i+1,x})\bigr ) \end{aligned}$$

for all *i*. To show (65) it thus remains to recall that the equation $\dim (C)=2{{\,\mathrm{rank}\,}}(d)+\dim (\ker (d)/{{\,\mathrm{img}\,}}(d))$ holds for every finite dimensional vector space *C* and every linear map $d:C\rightarrow C$ satisfying $d^2=0$. We have equality in (65) if and only if we have equality in (66) for all *i*. In this case, the codifferential is of maximal rank and ${\mathcal {H}}_{x,i}\cong \ker ({\tilde{A}}_{i,x})/{{\,\mathrm{img}\,}}({\tilde{A}}_{i-1,x})$.

#### Remark 4.12

If ${\tilde{A}}_i{\tilde{A}}_{i-1}=0$, then there exists a Kostant type codifferential of maximal rank. To construct such a codifferential, fix fiber-wise graded Hermitian inner products on the graded vector bundles ${{\,\mathrm{gr}\,}}(E_i)$, let ${\tilde{A}}_i^*:{{\,\mathrm{gr}\,}}_*(E_{i+1})\rightarrow {{\,\mathrm{gr}\,}}_{*-k_i}(E_i)$ denote the fiber-wise adjoint of ${\tilde{A}}_i:{{\,\mathrm{gr}\,}}_*(E_i)\rightarrow {{\,\mathrm{gr}\,}}_{*+k_i}(E_{i+1})$, and consider $\delta _i:=S_{i-1}{\tilde{A}}_{i-1}^*S_i^{-1}$ where $S_i:{{\,\mathrm{gr}\,}}(E_i)\rightarrow E_i$ are some splittings for the filtrations. In this case ${\tilde{\Box }}_i={\tilde{A}}_i^*{\tilde{A}}_i+{\tilde{A}}_{i-1}{\tilde{A}}_{i-1}^*$, hence ${{\,\mathrm{img}\,}}({\tilde{P}}_{i,x})=\ker ({\tilde{\Box }}_{i,x})=\ker ({\tilde{A}}_{i,x})\cap \ker ({\tilde{\delta }}_{i,x})$ and

$$\begin{aligned} {{\,\mathrm{gr}\,}}(E_{i,x})={{\,\mathrm{img}\,}}({\tilde{A}}_{i-1,x})\oplus {{\,\mathrm{img}\,}}({\tilde{P}}_{i,x})\oplus {{\,\mathrm{img}\,}}({\tilde{\delta }}_{i+1,x}) \end{aligned}$$

for each $x\in M$. We conclude that $\delta$ is a Kostant type codifferential of maximal rank, see Definition 4.6 and Remark 4.11. If the dimension of $\ker ({\tilde{A}}_{i,x})/{{\,\mathrm{img}\,}}({\tilde{A}}_{i-1,x})$ is locally constant for all *i*, then $\delta _{i,x}$ has locally constant rank for all *i*; hence, $\delta _i$ meets the assumptions in Proposition 4.8, we obtain a sequence of operators ${\bar{D}}_i$ as in (64), acting between sections of the vector bundles ${\mathcal {H}}_i\cong \ker ({\tilde{A}}_i)/{{\,\mathrm{img}\,}}({\tilde{A}}_{i-1})$, and this is a graded Rockland sequence provided the original sequence $A_i$ was.

### Linear connections on filtered manifolds

In this section we consider a linear connection on a filtered vector bundle over a filtered manifold and its extension to bundle valued differential forms. We will show that this induces a graded Rockland sequence in the sense of Definition 4.2 provided the linear connection is filtration-preserving and its curvature is contained in filtration degree one, see Proposition 4.15.

Suppose *E* is a filtered vector bundle over a filtered manifold *M*. We consider the induced filtration on the vector bundles $\Lambda ^kT^*M\otimes E$. To be explicit, $\psi \in \Lambda ^kT^*_xM\otimes E_x$ is in filtration degree *p* iff, for all tangent vectors $X_i\in T^{p_i}_xM$, we have $\psi (X_1,\dotsc ,X_k)\in E_x^{p+p_1+\cdots +p_k}$. Let us introduce the following notation for the associated graded vector bundle:

67 $$\begin{aligned} C^k(M;E):= {{\,\mathrm{gr}\,}}(\Lambda ^kT^*M\otimes E)=\Lambda ^k{\mathfrak {t}}^*M\otimes {{\,\mathrm{gr}\,}}(E). \end{aligned}$$

We will denote the grading by $C^k(M;E)=\bigoplus _pC^k(M;E)_p$.

Suppose $\nabla$ is a linear connection on *E* such that $\nabla _X\psi \in \Gamma ^\infty (E^{p+q})$ for all $X\in \Gamma ^\infty (T^pM)$ and $\psi \in \Gamma ^\infty (E^q)$. In other words, $\nabla :\Gamma ^\infty (E)\rightarrow \Omega ^1(M;E)$, is assumed to be filtration-preserving. It is easy to see that filtration-preserving connections always exist, the space of all such connections is affine over the space of filtration-preserving vector bundle homomorphisms $E\rightarrow T^*M\otimes E$. The Leibniz rule implies that the induced operator on the associated graded bundles, $\omega :={{\,\mathrm{gr}\,}}(\nabla ):\Gamma ^\infty ({{\,\mathrm{gr}\,}}(E))\rightarrow \Gamma ^\infty ({{\,\mathrm{gr}\,}}(T^*M\otimes E))$, is tensorial, i.e.,

$$\begin{aligned} \omega \in \Gamma ^\infty \bigl (C^1(M;{{\,\mathrm{end}\,}}(E))_0\bigr ) \end{aligned}$$

where $C^1(M;{{\,\mathrm{end}\,}}(E))={{\,\mathrm{gr}\,}}(T^*M\otimes {{\,\mathrm{end}\,}}(E))={\mathfrak {t}}^*M\otimes {{\,\mathrm{end}\,}}({{\,\mathrm{gr}\,}}(E))$ with 0-th grading component $C^1(M;{{\,\mathrm{end}\,}}(E))_0=\bigoplus _{p,q}({\mathfrak {t}}^pM)^*\otimes \hom ({{\,\mathrm{gr}\,}}_q(E),{{\,\mathrm{gr}\,}}_{p+q}(E))$. Let

68 $$\begin{aligned} d^\nabla :\Omega ^*(M;E)\rightarrow \Omega ^{*+1}(M;E) \end{aligned}$$

denote the usual extension of $\nabla$ characterized by the Leibniz rule,

69 $$\begin{aligned} d^\nabla (\alpha \wedge \psi )=d\alpha \wedge \psi +(-1)^k\alpha \wedge d^\nabla \psi , \end{aligned}$$

for all $\alpha \in \Omega ^k(M)$ and all $\psi \in \Omega ^*(M;E)$. Recall the explicit formula

70 $$\begin{aligned} (d^\nabla \psi )(X_0,\dotsc ,X_k)= & {} \sum _{i=0}^k(-1)^i\nabla _{X_i}\psi (X_0,\dotsc ,{\hat{i}},\dotsc ,X_k) \nonumber \\&+\sum _{0\le i<j\le k}(-1)^{i+j}\psi ([X_i,X_j],X_0,\dotsc ,{\hat{i}},\dotsc ,{\hat{j}},\dotsc ,X_k)\nonumber \\ \end{aligned}$$

for $\psi \in \Omega ^k(M;E)$ and vector fields $X_0,\dotsc ,X_k$.

#### Lemma 4.13

Let $\nabla :\Gamma ^\infty (E)\rightarrow \Omega ^1(M;E)$ be a filtration-preserving linear connection on a filtered vector bundle *E* over a filtered manifold *M*. Then, the extension $d^\nabla :\Omega ^k(M;E)\rightarrow \Omega ^{k+1}(M;E)$ is a differential operator of graded Heisenberg order at most zero. Moreover, the graded principal Heisenberg symbol at $x\in M$ fits into the following commutative diagram:Here, the horizontal identifications are obtained by tensorizing the identity on ${{\,\mathrm{gr}\,}}(E_x)$ with the identification $C^\infty ({\mathcal {T}}_xM,\Lambda ^k{\mathfrak {t}}_x^*M)=\Omega ^k({\mathcal {T}}_xM)$ induced by left trivialization of the tangent bundle of the group ${\mathcal {T}}_xM$. Moreover, $\omega _x\in C^1_x(M;{{\,\mathrm{end}\,}}(E))_0$ is considered as a left invariant ${{\,\mathrm{end}\,}}({{\,\mathrm{gr}\,}}(E_x))$-valued 1-form on ${\mathcal {T}}_xM$.

#### Proof

This follows readily from (70) and (9). Alternatively, this can be understood as follows. Let $A:E\rightarrow T^*M\otimes E$ be a filtration-preserving vector bundle homomorphism. Then, $\nabla +A$ is another filtration-preserving linear connection on *E*, and $\omega ^{\nabla +A}=\omega ^\nabla +{\tilde{A}}$ where ${\tilde{A}}={{\,\mathrm{gr}\,}}(A):{{\,\mathrm{gr}\,}}(E)\rightarrow {{\,\mathrm{gr}\,}}(T^*M\otimes E)$ denotes the associate graded vector bundle homomorphism induced by *A*. Moreover, $d^{\nabla +A}=d^\nabla +A\wedge -$ and thus ${\tilde{\sigma }}^0_x(d^{\nabla +A})={\tilde{\sigma }}^0_x(d^\nabla )+{\tilde{A}}_x\wedge -$. We conclude that the statement of the lemma holds for $\nabla$ iff it holds for $\nabla +A$. Since this statement is local, and since the space of filtration-preserving linear connections on *E* is affine over the space of filtration-preserving vector bundle homomorphisms $E\rightarrow T^*M\otimes E$, we may w.l.o.g. assume that $\nabla$ is the trivial connection on a trivial bundle $E=M\times E_0$. By compatibility with direct sums, it suffices to consider the trivial line bundle $E=M\times {\mathbb {C}}$; that is, we may assume $d^\nabla =d$, the de Rham differential on $\Omega ^*(M)$. In view of the Leibniz rule, it suffices to show that $d:\Omega ^k(M)\rightarrow \Omega ^{k+1}(M)$ has graded Heisenberg order at most zero and ${\tilde{\sigma }}^0_x(d)=d$, for $k=0,1$. This, however, can readily be checked. $\square$

Using the Leibniz rule (or Lemma 4.13 above), one readily checks that the differential operator (68) is filtration-preserving. Moreover, the operator induced on the associated graded is tensorial, given by a vector bundle homomorphism of bidegree (1, 0),

71 $$\begin{aligned} \partial ^\omega :C^k(M;E)_p\rightarrow C^{k+1}(M;E)_p,\qquad \partial ^\omega :={{\,\mathrm{gr}\,}}(d^\nabla ), \end{aligned}$$

which can be characterized as the unique extension of $C^0(M;E)\xrightarrow \omega C^1(M;E)$ satisfying the Leibniz rule

72 $$\begin{aligned} \partial ^\omega (\alpha \wedge \psi ) =\partial \alpha \wedge \psi +(-1)^k\alpha \wedge \partial ^\omega \psi , \end{aligned}$$

for all $\alpha \in \Lambda ^k{\mathfrak {t}}^*_xM$ and $\psi \in C_x^*(M;E)$. Here $\partial :\Lambda ^k{\mathfrak {t}}^*M\rightarrow \Lambda ^{k+1}{\mathfrak {t}}^*M$ denotes the fiber-wise Chevalley–Eilenberg differential. More explicitly, we have

73 $$\begin{aligned} \partial ^\omega =\partial \otimes {{\,\mathrm{id}\,}}_E+\omega \wedge -. \end{aligned}$$

#### Lemma 4.14

For each $x\in M$ the following are equivalent:

- The curvature $F^\nabla _x\in \Omega _x^2(M;{{\,\mathrm{end}\,}}(E))$ is contained in filtration degree one, that is, for all $X_i\in T_x^{p_i}M$ and $\psi \in E^p_x$ we have $F_x^\nabla (X_1,X_2)\psi \in E^{p+p_1+p_2+1}_x$.
- $\omega _x:{\mathfrak {t}}_xM\rightarrow {{\,\mathrm{end}\,}}({{\,\mathrm{gr}\,}}(E_x))$ provides a graded representation of the graded nilpotent Lie algebra ${\mathfrak {t}}_xM$ on the graded vector space ${{\,\mathrm{gr}\,}}(E_x)$.
- $$(\partial _x^\omega )^2=0$$.
- $${\tilde{\sigma }}^0_x(d^\nabla )^2=0$$.

In this case, the Chevalley–Eilenberg differential of the Lie algebra ${\mathfrak {t}}_xM$ with coefficients in the representation ${{\,\mathrm{gr}\,}}(E_x)$ coincides with (71) at the point *x*.

#### Proof

Recall that $(d^\nabla )^2=F^\nabla \wedge -$ is tensorial. Using (41) and Remark 4.1, we obtain

$$\begin{aligned} {\tilde{\sigma }}^0_x(d^\nabla )^2 ={\tilde{\sigma }}_x^0((d^\nabla )^2) ={{\,\mathrm{gr}\,}}_x((d^\nabla )^2) ={{\,\mathrm{gr}\,}}_x(d^\nabla )^2 =(\partial _x^\omega )^2, \end{aligned}$$

whence the equivalences (a)$\Leftrightarrow$(c)$\Leftrightarrow$(d). To see the equivalence (b)$\Leftrightarrow$(c), we note that (72) and $\partial ^2=0$ give $(\partial _x^\omega )^2(\alpha \wedge \psi )=\alpha \wedge (\partial _x^\omega )^2\psi$. Furthermore, using (73) one readily shows

$$\begin{aligned} \bigl ((\partial _x^\omega )^2\phi \bigr )(X,Y)=\bigl (\omega (X)\omega (Y)-\omega (Y)\omega (X)-\omega ([X,Y])\bigr )\phi , \end{aligned}$$

for $$\phi \in E_x=C^0_x(M;E)$$ and $$X,Y\in {\mathfrak {t}}_xM$$. $$\square $$

For flat connections (on nilpotent Lie groups) the following is due to Rumin, see [69, Theorem 5.2] and [67, Theorem 3].

#### Proposition 4.15

Let $\nabla :\Gamma ^\infty (E)\rightarrow \Omega ^1(M;E)$ be a filtration-preserving linear connection on a filtered vector bundle *E* over a filtered manifold *M*. If the curvature of $\nabla$ is contained in filtration degree one, see Lemma 4.14, then

74 $$\begin{aligned} \cdots \rightarrow \Omega ^{k-1}(M;E)\xrightarrow {d^\nabla }\Omega ^k(M;E) \xrightarrow {d^\nabla }\Omega ^{k+1}(M;E)\rightarrow \cdots \end{aligned}$$

is a graded Rockland sequence.

#### Proof

Fix $x\in M$, consider the nilpotent Lie group $G:={\mathcal {T}}_xM$ with Lie algebra ${\mathfrak {g}}:={\mathfrak {t}}_xM$ and the ${\mathfrak {g}}$-module $V:={{\,\mathrm{gr}\,}}(E_x)$, see Lemma 4.14. Hence, $C_x^k(M;E)=\Lambda ^k{\mathfrak {g}}^*\otimes V$. According to Lemma 4.13, the Heisenberg principal symbol sequence

$$\begin{aligned} \cdots \rightarrow C^\infty (G,\Lambda ^k{\mathfrak {g}}^*\otimes V)\xrightarrow {{\tilde{\sigma }}_x^0(d^\nabla )} C^\infty (G,\Lambda ^{k+1}{\mathfrak {g}}^*\otimes V)\rightarrow \cdots \end{aligned}$$

is isomorphic to the Chevalley–Eilenberg complex of the Lie algebra ${\mathfrak {g}}$ with values in the ${\mathfrak {g}}$-representation $C^\infty (G)\otimes V$. More explicitly, if $X_j$ is a basis of ${\mathfrak {g}}$ and $\alpha ^j$ denotes the dual basis of ${\mathfrak {g}}^*$ then, in ${\mathcal {U}}({\mathfrak {g}})\otimes \hom \bigl (\Lambda ^k{\mathfrak {g}}^*\otimes V,\Lambda ^{k+1}{\mathfrak {g}}^*\otimes V\bigr )$, we have

75 $$\begin{aligned} {\tilde{\sigma }}_x^0(d^\nabla )=\sum _jX_j\otimes e_{\alpha ^j}+\sum _j1\otimes e_{d\alpha ^j}i_{X_j}+1\otimes \omega _x \end{aligned}$$

Here $e_{\alpha ^j}\in \hom \bigl (\Lambda ^k{\mathfrak {g}}^*\otimes V,\Lambda ^{k+1}{\mathfrak {g}}^*\otimes V\bigr )$ denotes the exterior product with $\alpha ^j\in {\mathfrak {g}}^*$; $i_{X_j}\in \hom \bigl (\Lambda ^k{\mathfrak {g}}^*\otimes V,\Lambda ^{k-1}{\mathfrak {g}}^*\otimes V\bigr )$ denotes the contraction with $X_j\in {\mathfrak {g}}$; $e_{d\alpha ^j}\in \hom \bigl (\Lambda ^{k-1}{\mathfrak {g}}^*\otimes V,\Lambda ^{k+1}{\mathfrak {g}}^*\otimes V\bigr )$ denotes the exterior product with $d\alpha ^j\in \Lambda ^2{\mathfrak {g}}^*$; and $\omega _x\in \hom \bigl (\Lambda ^k{\mathfrak {g}}^*\otimes V,\Lambda ^{k+1}{\mathfrak {g}}^*\otimes V\bigr )$ denotes the exterior product with the representation $\omega _x:{\mathfrak {g}}\rightarrow {{\,\mathrm{end}\,}}(V)$.

Suppose $\pi :G\rightarrow U({\mathcal {H}})$ is a non-trivial irreducible unitary representation on a Hilbert space ${\mathcal {H}}$, and let ${\mathcal {H}}_\infty$ denote the space of smooth vectors. Using (75) one readily checks, see Sect. 2.2, that the sequence

$$\begin{aligned} \cdots \rightarrow {\mathcal {H}}_\infty \otimes \Lambda ^k{\mathfrak {g}}^*\otimes V\xrightarrow {\pi ({\tilde{\sigma }}^0_x(d^\nabla ))}{\mathcal {H}}_\infty \otimes \Lambda ^{k+1}{\mathfrak {g}}^*\otimes V\rightarrow \cdots \end{aligned}$$

is isomorphic to the Chevalley–Eilenberg complex of the Lie algebra ${\mathfrak {g}}$ with values in the ${\mathfrak {g}}$-representation ${\mathcal {H}}_\infty \otimes V$. Consequently, it remains to show that the Lie algebra cohomology of ${\mathfrak {g}}$ with coefficients in the ${\mathfrak {g}}$-module ${\mathcal {H}}_\infty \otimes V$ vanishes, that is, $H^*({\mathfrak {g}};{\mathcal {H}}_\infty \otimes V)=0$.

Since ${\mathfrak {g}}$ is nilpotent, there exists a 1-dimensional subspace $W\subseteq V$ on which ${\mathfrak {g}}$ acts trivially. The corresponding short exact sequence of ${\mathfrak {g}}$-modules, $0\rightarrow W\rightarrow V\rightarrow V/W\rightarrow 0$, yields a short exact sequence of ${\mathfrak {g}}$-modules $0\rightarrow {\mathcal {H}}_\infty \rightarrow {\mathcal {H}}_\infty \otimes V\rightarrow {\mathcal {H}}_\infty \otimes V/W\rightarrow 0$ which, in turn, gives rise to a long exact sequence:

$$\begin{aligned} \cdots \rightarrow H^q({\mathfrak {g}};{\mathcal {H}}_\infty )\rightarrow H^q({\mathfrak {g}};{\mathcal {H}}_\infty \otimes V)\rightarrow H^q({\mathfrak {g}};{\mathcal {H}}_\infty \otimes V/W)\xrightarrow \partial H^{q+1}({\mathfrak {g}};{\mathcal {H}}_\infty )\rightarrow \cdots \end{aligned}$$

Hence, by induction on the dimension of *V*, it suffices to show $H^q({\mathfrak {g}};{\mathcal {H}}_\infty )=0$, for all *q*. The statement thus follows from Lemma 4.16. $\square$

#### Lemma 4.16

Consider a non-trivial irreducible unitary representation of a finite dimensional simply connected nilpotent Lie group *G* on a Hilbert space ${\mathcal {H}}$, and the associated representation of the corresponding Lie algebra ${\mathfrak {g}}$ on the space of smooth vectors ${\mathcal {H}}_\infty$. Then, the Lie algebra cohomology of ${\mathfrak {g}}$ with coefficients in ${\mathcal {H}}_\infty$ is trivial, that is, $H^*({\mathfrak {g}};{\mathcal {H}}_\infty )=0$.

#### Proof

The proof proceeds by induction on the dimension of ${\mathfrak {g}}$. Since ${\mathfrak {g}}$ is nilpotent, there exists a 1-dimensional central subalgebra ${\mathfrak {z}}\subseteq {\mathfrak {g}}$. Recall that there is a Hochschild–Serre spectral sequence [46] converging to $H^*({\mathfrak {g}};{\mathcal {H}}_\infty )$ with $E_2$-term

$$\begin{aligned} E_2^{p,q}\cong H^p\bigl ({\mathfrak {g}}/{\mathfrak {z}};H^q({\mathfrak {z}};{\mathcal {H}}_\infty )\bigr ). \end{aligned}$$

Since the representation of *G* on the Hilbert space ${\mathcal {H}}$ is irreducible, ${\mathfrak {z}}$ acts by scalars on ${\mathcal {H}}_\infty$, see [49, Theorem 5 in Appendix V]. If this action is non-trivial, then $H^*({\mathfrak {z}};{\mathcal {H}}_\infty )=0$ and, consequently, the $E_2$-term vanishes. We may thus assume that the action of ${\mathfrak {z}}$ on ${\mathcal {H}}_\infty$ is trivial. Hence, $H^*({\mathfrak {z}};{\mathcal {H}}_\infty )=H^0({\mathfrak {z}};{\mathcal {H}}_\infty )\oplus H^1({\mathfrak {z}};{\mathcal {H}}_\infty )\cong {\mathcal {H}}_\infty \oplus {\mathcal {H}}_\infty$ as ${\mathfrak {g}}/{\mathfrak {z}}$-modules.

Consider the closed connected central subgroup $Z:=\exp ({\mathfrak {z}})$ in *G*, and note that *G*/*Z* is a simply connected nilpotent Lie group with Lie algebra ${\mathfrak {g}}/{\mathfrak {z}}$. Since ${\mathcal {H}}_\infty$ is dense in ${\mathcal {H}}$, the subgroup *Z* acts trivially on ${\mathcal {H}}$, see [49, Theorem 4 in Appendix V]. Hence, the representation of *G* factors through a representation of *G*/*Z* on ${\mathcal {H}}$. Clearly, this is a non-trivial irreducible unitary representation of *G*/*Z* whose space of smooth vectors coincides with ${\mathcal {H}}_\infty$. Hence, $H^*({\mathfrak {g}}/{\mathfrak {z}};{\mathcal {H}}_\infty )=0$, by induction. We conclude $H^p({\mathfrak {g}}/{\mathfrak {z}};H^*({\mathfrak {z}};{\mathcal {H}}_\infty ))\cong H^p({\mathfrak {g}}/{\mathfrak {z}};{\mathcal {H}}_\infty \oplus {\mathcal {H}}_\infty )=H^p({\mathfrak {g}}/{\mathfrak {z}};{\mathcal {H}}_\infty )\oplus H^p({\mathfrak {g}}/{\mathfrak {z}};{\mathcal {H}}_\infty )=0$. Thus, the $E_2$-term vanishes, and the proof is complete. $\square$

### Subcomplexes of the de Rham complex

In this section, we apply the observations and constructions from Sects. 4.2 and 4.3 to the de Rham sequence associated with a filtration-preserving linear connection. This directly leads to the main result of this section, see Theorem 4.17 and Corollary 4.18. In the subsequent Sect. 4.6, we will apply this to the curved BGG sequences in parabolic geometry which appear as a special case of the sequences considered here.

Let $\nabla$ be a filtration-preserving linear connection on a filtered vector bundle *E* over a filtered manifold *M*, and consider its extension to *E*-valued differential forms characterized by the Leibniz rule in (69),

76 $$\begin{aligned} \cdots \rightarrow \Omega ^{k-1}(M;E)\xrightarrow {d^\nabla _{k-1}}\Omega ^k(M;E)\xrightarrow {d^\nabla _k}\Omega ^{k+1}(M;E)\rightarrow \cdots . \end{aligned}$$

Consider a sequence of differential operators

77 $$\begin{aligned} \cdots \leftarrow \Omega ^{k-1}(M;E)\xleftarrow {\delta _k}\Omega ^k(M;E)\xleftarrow {\delta _{k+1}}\Omega ^{k+1}(M;E)\leftarrow \cdots \end{aligned}$$

which are of graded Heisenberg order at most zero. Then, the differential operator

$$\begin{aligned} \Box _k:\Omega ^k(M;E)\rightarrow \Omega ^k(M;E),\qquad \Box _k:=d^\nabla _{k-1}\delta _k+\delta _{k+1}d^\nabla _k, \end{aligned}$$

is of graded Heisenberg order at most zero. Let

$$\begin{aligned} \partial _k^\omega&:C^k(M;E)\rightarrow C^{k+1}(M;E),&\partial _k^\omega&:={{\,\mathrm{gr}\,}}(d^\nabla _k), \\ {\tilde{\delta }}_k&:C^k(M;E)\rightarrow C^{k-1}(M;E),&{\tilde{\delta }}_k&:={{\,\mathrm{gr}\,}}(\delta _k), \\ {\tilde{\Box }}_k&:C^k(M;E)\rightarrow C^k(M;E),&{\tilde{\Box }}_k&:={{\,\mathrm{gr}\,}}(\Box _k)=\partial ^\omega _{k-1}{\tilde{\delta }}_k+{\tilde{\delta }}_{k+1}\partial ^\omega _k, \end{aligned}$$

denote the associated graded vector bundle homomorphisms, see Remark 4.1, (67), and (71).

For each $x\in M$, let ${\tilde{P}}_{k,x}:C_x^k(M;E)\rightarrow C_x^k(M;E)$ denote the spectral projection onto the generalized zero eigenspace of ${\tilde{\Box }}_{x,k}:C_x^k(M;E)\rightarrow C^k_x(M;E)$, the restriction of ${\tilde{\Box }}_k$ to the fiber over *x*. Assume that the rank of ${\tilde{P}}_{k,x}$ is locally constant in *x* for each *k*. Then, see Lemma 4.4(a), ${\tilde{P}}_k:C^k(M;E)\rightarrow C^k(M;E)$ is a smooth vector bundle homomorphism, ${\tilde{P}}_k^2={\tilde{P}}_k$, ${\tilde{P}}_k{\tilde{\Box }}_k={\tilde{\Box }}_k{\tilde{P}}_k$, and we obtain a decomposition of graded vector bundles,

78 $$\begin{aligned} C^k(M;E)={{\,\mathrm{img}\,}}({\tilde{P}}_k)\oplus \ker ({\tilde{P}}_k), \end{aligned}$$

which is invariant under ${\tilde{\Box }}_k$. Moreover, ${\tilde{\Box }}_k$ is nilpotent on ${{\,\mathrm{img}\,}}({\tilde{P}}_k)$ and invertible on $\ker ({\tilde{P}}_k)$.

According to Lemma 4.4(b), there exists a unique filtration-preserving differential operator

$$\begin{aligned} P_k:\Omega ^k(M;E)\rightarrow \Omega ^k(M;E) \end{aligned}$$

such that $P_k^2=P_k$, $P_k\Box _k=\Box _kP_k$, and ${{\,\mathrm{gr}\,}}(P_k)={\tilde{P}}_k$. This operator $P_k$ has graded Heisenberg order at most zero and provides a decomposition of filtered vector spaces,

79 $$\begin{aligned} \Omega ^k(M;E)={{\,\mathrm{img}\,}}(P_k)\oplus \ker (P_k), \end{aligned}$$

invariant under $\Box _k$ and such that $\Box _k$ is nilpotent on ${{\,\mathrm{img}\,}}(P_k)$ and invertible on $\ker (P_k)$. If

$$\begin{aligned} C^k(M,E)={{\,\mathrm{gr}\,}}(\Lambda ^kT^*M\otimes E)\xrightarrow {S_k}\Lambda ^kT^*M\otimes E, \end{aligned}$$

are splittings of the filtrations, then according to Lemma 4.4(c)

80 $$\begin{aligned} L_k:\Gamma ^\infty (C^k(M;E))\rightarrow \Omega ^k(M;E),\qquad L_k:=P_kS_k{\tilde{P}}_k+({{\,\mathrm{id}\,}}-P_k)S_k({{\,\mathrm{id}\,}}-{\tilde{P}}_k), \end{aligned}$$

is an invertible differential operator of graded Heisenberg order at most zero such that ${{\,\mathrm{gr}\,}}(L_k)={{\,\mathrm{id}\,}}$ and $L^{-1}_kP_kL_k={\tilde{P}}_k$. Hence, $L_k$ induces filtration-preserving isomorphisms

$$\begin{aligned} L_k:\Gamma ^\infty ({{\,\mathrm{img}\,}}({\tilde{P}}_k))\xrightarrow \cong {{\,\mathrm{img}\,}}(P_k) \qquad \text {and}\qquad L_k:\Gamma ^\infty (\ker ({\tilde{P}}_k))\xrightarrow \cong \ker (P_k). \end{aligned}$$

Moreover, $L^{-1}_k\Box _kL_k$ is a differential operator of graded Heisenberg order at most zero preserving the decomposition (78) and satisfying ${{\,\mathrm{gr}\,}}(L^{-1}_k\Box _kL_k)={\tilde{\Box }}_k$. Putting

81 $$\begin{aligned} D_k:={\tilde{P}}_{k+1}L_{k+1}^{-1}d^\nabla _kL_k|_{\Gamma ^\infty ({{\,\mathrm{img}\,}}({\tilde{P}}_k))} \qquad \text {and}\qquad B_k:=({{\,\mathrm{id}\,}}-{\tilde{P}}_{k+1})L_{k+1}^{-1}d^\nabla _kL_k|_{\Gamma ^\infty (\ker ({\tilde{P}}_k))}\nonumber \\ \end{aligned}$$

we obtain two sequences of differential operators,

82 $$\begin{aligned} \cdots \rightarrow \Gamma ^\infty ({{\,\mathrm{img}\,}}({\tilde{P}}_{k-1}))\xrightarrow {D_{k-1}}\Gamma ^\infty ({{\,\mathrm{img}\,}}({\tilde{P}}_k))\xrightarrow {D_k}\Gamma ^\infty ({{\,\mathrm{img}\,}}({\tilde{P}}_{k+1}))\rightarrow \cdots \end{aligned}$$

and

83 $$\begin{aligned} \cdots \rightarrow \Gamma ^\infty (\ker ({\tilde{P}}_{k-1}))\xrightarrow {B_{k-1}}\Gamma ^\infty (\ker ({\tilde{P}}_k))\xrightarrow {B_k}\Gamma ^\infty (\ker ({\tilde{P}}_{k+1}))\rightarrow \cdots , \end{aligned}$$

all of which have graded Heisenberg order at most zero.

Combining Proposition 4.5 with Proposition 4.15, we immediately obtain:

#### Theorem 4.17

In this situation the following hold true:

- If the curvature of $\nabla$ is contained in filtration degree one, cf. Lemma 4.14, then (82) and (83) are both graded Rockland sequences.
- If the curvature of $\nabla$ vanishes, then the operator $L_{k+1}^{-1}d^\nabla _kL_k$ decouples,
  $$\begin{aligned} L^{-1}_{k+1}d^\nabla _kL_k=D_k\oplus B_k, \end{aligned}$$
  and we have $D_kD_{k-1}=0$ as well as $B_kB_{k-1}=0$ for all *k*. In this situation, $G_k:\Gamma ^\infty (\ker ({\tilde{P}}_k))\rightarrow \Gamma ^\infty (\ker ({\tilde{P}}_k))$,
  $$\begin{aligned} G_k:=B_{k-1}({{\,\mathrm{id}\,}}-{\tilde{P}}_{k-1}){\tilde{\delta }}_k{\tilde{\Box }}_k^{-1} +({{\,\mathrm{id}\,}}-{\tilde{P}}_k){\tilde{\delta }}_{k+1}{\tilde{\Box }}_{k+1}^{-1}\partial ^\omega _k, \end{aligned}$$
  is an invertible differential operator of graded Heisenberg order at most zero with ${{\,\mathrm{gr}\,}}(G_k)={{\,\mathrm{id}\,}}$ that conjugates the complex (83) into an acyclic tensorial complex, namely,
  $$\begin{aligned} G_{k+1}^{-1}B_kG_k=\partial ^\omega _k|_{\Gamma ^\infty (\ker ({\tilde{P}}_k))}. \end{aligned}$$
  Moreover, the restriction $L_k:\Gamma ^\infty ({{\,\mathrm{img}\,}}({\tilde{P}}_k))\rightarrow \Omega ^k(M;E)$ provides a chain map, $d^\nabla _kL_k|_{\Gamma ^\infty ({{\,\mathrm{img}\,}}({\tilde{P}}_k))}=L_{k+1}D_k$, inducing an isomorphism between the cohomologies of (82) and (76). More precisely, $\pi _k:={\tilde{P}}_kL_k^{-1}:\Omega ^k(M;E)\rightarrow \Gamma ^\infty ({{\,\mathrm{img}\,}}({\tilde{P}}_k))$, is a chain map, $D_k\pi _k=\pi _{k+1}d^\nabla _k$, which is an inverse of $L_k$ up to homotopy, i.e., $\pi _kL_k|_{\Gamma ^\infty ({{\,\mathrm{img}\,}}({\tilde{P}}_k))}={{\,\mathrm{id}\,}}$ and ${{\,\mathrm{id}\,}}-L_k\pi _k=d^\nabla _{k-1}h_k+h_{k+1}d^\nabla _k$ where the homotopy $h_k:\Omega ^k(M;E)\rightarrow \Omega ^{k-1}(M;E)$ is a differential operator of graded Heisenberg order at most zero given by $h_k:=L_{k-1}G_{k-1}({{\,\mathrm{id}\,}}-{\tilde{P}}_k){\tilde{\delta }}_k{\tilde{\Box }}_k^{-1}G_k^{-1}({{\,\mathrm{id}\,}}-{\tilde{P}}_k)L_k^{-1}$.

For flat connections the preceding theorem is due to Rumin, see [69, Theorems 2.6 and 5.2] or [67, Theorems 1 and 3].

Combining Proposition 4.8 with Proposition 4.15 we immediately obtain:

#### Corollary 4.18

Let $\nabla$ be a filtration-preserving linear connection on a filtered vector bundle *E* over a filtered manifold *M* whose curvature is contained in filtration degree one, and consider its extension to *E*-valued differential forms characterized by the Leibniz rule, see (69),

84 $$\begin{aligned} \cdots \rightarrow \Omega ^{k-1}(M;E)\xrightarrow {d^\nabla }\Omega ^k(M;E)\xrightarrow {d^\nabla }\Omega ^{k+1}(M;E)\rightarrow \cdots . \end{aligned}$$

Moreover, consider a codifferential of Kostant type, see Definition 4.6,

$$\begin{aligned} \cdots \leftarrow \Lambda ^{k-1}T^*M\otimes E\xleftarrow {\delta _i}\Lambda ^kT^*M\otimes E\xleftarrow {\delta _{i+1}}\Lambda ^{k+1}T^*M\otimes E\leftarrow \cdots , \end{aligned}$$

which has locally constant rank, and let ${\bar{\pi }}_k:\ker (\delta _k)\rightarrow {\mathcal {H}}_k:=\ker (\delta _k)/{{\,\mathrm{img}\,}}(\delta _{k+1})$ denote the canonical vector bundle projection. Then, the following hold true:

- There exists a unique differential operator ${\bar{L}}_k:\Gamma ^\infty ({\mathcal {H}}_k)\rightarrow \Omega ^k(M;E)$ such that $\delta _k{\bar{L}}_k=0$, ${\bar{\pi }}_k{\bar{L}}_k={{\,\mathrm{id}\,}}$, and $\delta _{k+1}d^\nabla _k{\bar{L}}_k=0$. Moreover, ${\bar{L}}_k$ is of graded Heisenberg order at most zero.
- The differential operator
  $$\begin{aligned} {\bar{D}}_k:\Gamma ^\infty ({\mathcal {H}}_k)\rightarrow \Gamma ^\infty ({\mathcal {H}}_{k+1}),\qquad {\bar{D}}_k:={\bar{\pi }}_{k+1}d^\nabla _kL_k, \end{aligned}$$
  is of graded Heisenberg order at most zero, and
  85 $$\begin{aligned} \cdots \rightarrow \Gamma ^\infty ({\mathcal {H}}_{k-1})\xrightarrow {{\bar{D}}_{k-1}}\Gamma ^\infty ({\mathcal {H}}_k)\xrightarrow {{\bar{D}}_k}\Gamma ^\infty ({\mathcal {H}}_{k+1})\rightarrow \cdots \end{aligned}$$
  is graded Rockland sequence.
- If $\nabla$ has vanishing curvature, then ${\bar{D}}_k{\bar{D}}_{k-1}=0$, and ${\bar{L}}_k:\Gamma ^\infty ({\mathcal {H}}_k)\rightarrow \Omega ^k(M;E)$ provides a chain map, $d^\nabla _k{\bar{L}}_k={\bar{L}}_{k+1}{\bar{D}}_k$, inducing an isomorphism between the cohomologies of (85) and (84).
- There exist invertible differential operators, $V_i:\Gamma ^\infty ({{\,\mathrm{img}\,}}({\tilde{P}}_i))\rightarrow \Gamma ^\infty ({\mathcal {H}}_i)$ with inverse $V_i^{-1}:\Gamma ^\infty ({\mathcal {H}}_i)\rightarrow \Gamma ^\infty ({{\,\mathrm{img}\,}}({\tilde{P}}_i))$, both of graded Heisenberg order at most zero, such that
  $$\begin{aligned} V_{i+1}^{-1}{\bar{D}}_iV_i=D_i, \end{aligned}$$
  where $D_i:\Gamma ^\infty ({{\,\mathrm{img}\,}}({\tilde{P}}_i))\rightarrow \Gamma ^\infty ({{\,\mathrm{img}\,}}({\tilde{P}}_{i+1}))$ denotes the operator considered in Theorem 4.17, see (81), corresponding to splittings $S_k$ as in Definition 4.6(iii).

The operators ${\bar{L}}_k$ and ${\bar{D}}_k$ in Corollary 4.18 are direct generalizations of the splitting operators and the curved BGG operators in parabolic geometry, respectively. This aspect will be addressed in Sect. 4.6. The differential projector $P_k$ generalizes the operator obtained by composing (5.1) with (5.2) in [8].

Detailed explanations of how Rumin’s complex [65, 66] appears among the sequences (85) considered in Corollary 4.18 can be found in [22, Example 4.21]. Hypoellipticity of this sequence has been established by Rumin, see [66, Section 3]. Let us mention that Rumin and Seshadri [70] have introduced an analytic torsion based on hypoelliptic Laplacians associated with Rumin’s complex. We expect that their construction can be extended to all (ungraded) sequences appearing in Corollary 4.18(b).

We conclude this section with a 4-dimensional geometry for which the sequence in Theorem 4.17(b) turns out to be graded Rockland but not Rockland in the ungraded sense.

#### Example 4.19

(*Engel structures*) Recall that an Engel structure [60] on a smooth 4-manifold *M* is a smooth rank two distribution $T^{-1}M\subseteq TM$ with growth vector (2, 3, 4). More explicitly, Lie brackets of sections of $T^{-1}M$ generate a rank three bundle $T^{-2}M$, and triple brackets of sections of $T^{-1}M$ generate all of *TM*. Hence, *M* is a filtered manifold,

$$\begin{aligned} TM=T^{-3}M\supseteq T^{-2}M\supseteq T^{-1}M\supseteq T^0M=0. \end{aligned}$$

One readily checks that the bundle of osculating algebras is locally trivial with typical fiber isomorphic to the graded nilpotent Lie algebra ${\mathfrak {g}}={\mathfrak {g}}_{-3}\oplus {\mathfrak {g}}_{-2}\oplus {\mathfrak {g}}_{-1}$ with non-trivial brackets

$$\begin{aligned}{}[X_1,X_2]=X_3,\qquad [X_1,X_3]=X_4,\qquad [X_2,X_3]=0, \end{aligned}$$

where $X_1,X_2$ is a basis of ${\mathfrak {g}}_{-1}$, $X_3$ is a basis of ${\mathfrak {g}}_{-2}$ and $X_4$ is a basis of ${\mathfrak {g}}_{-3}$. If *G* denotes the simply connected nilpotent Lie group with Lie algebra ${\mathfrak {g}}$, then the left invariant distribution corresponding to ${\mathfrak {g}}_{-1}$ provides an Engel structure on *G*. Locally, every Engel structure is diffeomorphic to this left invariant structure. According to a result of Vogel [80], every closed parallelizable smooth 4-manifold admits an (orientable) Engel structure.

We will exhibit explicit formulas for the graded Heisenberg principal symbol of the operators $D_k$ in Theorem 4.17 corresponding to the de Rham complex, that is, the trivial flat line bundle *E*. We work on $M=G$ equipped with the left invariant Engel structure mentioned before. We use the left invariant splitting for $S:{{\,\mathrm{gr}\,}}(\Lambda ^kT^*G)\rightarrow \Lambda ^kT^*G$ provided by the decomposition ${\mathfrak {g}}={\mathfrak {g}}_{-3}\oplus {\mathfrak {g}}_{-2}\oplus {\mathfrak {g}}_{-1}$. Moreover, we consider the left invariant codifferential $\delta _k:\Lambda ^kT^*G\rightarrow \Lambda ^{k-1}T^*G$ which, at the identity, is dual to the Chevalley–Eilenberg differential $\partial _{k-1}:\Lambda ^{k-1}{\mathfrak {g}}^*\rightarrow \Lambda ^k{\mathfrak {g}}^*$ with respect to the basis $X_1,\dotsc ,X_4$. Clearly, this is a Kostant type codifferential of maximal rank. In this situation, Theorem 4.17(b) provides a graded Rockland complex of left invariant operators, cf. [6] and [69, Section 2.3]:

86 $$\begin{aligned} C^\infty (G)\xrightarrow {D_0} C^\infty (G)^2\xrightarrow {D_1} \begin{array}{c}C^\infty (G)\\ \oplus \\ C^\infty (G)\end{array}\xrightarrow {D_2} C^\infty (G)^2\xrightarrow {D_3} C^\infty (G). \end{aligned}$$

Using matrices with entries in the universal enveloping algebra of ${\mathfrak {g}}$, and using the notation $X_{i_1\dotsc i_k}=X_{i_1}\cdots X_{i_k}$, these operators can be expressed as:

$$\begin{aligned} D_0= & {} \left( \begin{array}{c}X_1\\ X_2\end{array}\right) \\ D_1= & {} \left( \begin{array}{cc}-X_{22}&{}X_{12}-2X_3\\ \hline -X_4-X_{112}-X_{13}&{}X_{111}\end{array}\right) \\ D_2= & {} \left( \begin{array}{c|c}X_{111}&{}-X_3-X_{12}\\ 3X_4-3X_{13}+X_{112}&{}-X_{22}\end{array}\right) \\ D_3= & {} \left( \begin{array}{cc}-X_2&X_1\end{array}\right) \end{aligned}$$

A detailed verification of these claims can be found in [22, Appendix A]. The direct sum symbol in the sequence (86) indicates that its filtration is non-trivial. Correspondingly, the operators $D_1$ and $D_2$ are not homogeneous, whence the lines in their matrices separating different degrees. Evidently, the sequence (86) fails to be Rockland in the ungraded sense.

### BGG sequences

Every regular parabolic geometry has an underlying filtered manifold *M* whose bundle of osculating Lie algebras is locally trivial. The Cartan connection induces a linear connection $\nabla$ on every associated tractor bundle *E* over *M*. This linear connection is filtration-preserving and its curvature is contained in filtration degree one. Moreover, Kostant’s codifferential provides a vector bundle homomorphism $\delta :\Lambda ^kT^*M\otimes E\rightarrow \Lambda ^{k-1}T^*M\otimes E$, satisfying the assumptions in Corollary 4.18. In this situation, the operator ${\bar{L}}$ in Corollary 4.18(a) coincides with the splitting operator in [12, Theorem 2.4], see also [8, 11]. Furthermore, the sequence in Corollary 4.18(b) reduces to the “torsion free BGG sequence” in [8, Section 5] and coincides with the sequence considered in [12, Section 2.4]. For torsion free parabolic geometries this coincides with the original curved BGG sequence constructed by Čap et al. [11]. From Corollary 4.18(b), we conclude that these BGG operators have graded Heisenberg order at most zero and form a graded Rockland sequence, see Corollary 4.20.

Let us point out that there is a normalization condition for the curvature of the Cartan connection such that normal regular parabolic geometries can equivalently be described by underlying geometric structures, see Theorem 3.1.14, Section 3.1.16, and the historical remarks at the end of Section 3 in [10]. For a large class of parabolic geometries this underlying geometric structure consists merely of the underlying filtered manifold, [10, Proposition 4.3.1]. These provide intriguing classes of filtered manifolds to which one can associate graded Rockland sequences of differential operators in a natural way.

In the remaining part of this section we will, for the reader’s convenience, briefly recall basic facts on parabolic geometries and provided detailed references supporting the claims made above. We closely follow the presentation in [10, 11] and [12, Section 2].

Consider a |*k*|-graded semisimple Lie algebra

87 $$\begin{aligned} {\mathfrak {g}}={\mathfrak {g}}_{-k}\oplus \cdots \oplus {\mathfrak {g}}_{-1}\oplus {\mathfrak {g}}_0\oplus {\mathfrak {g}}_1\oplus \cdots \oplus {\mathfrak {g}}_k. \end{aligned}$$

More precisely, $[{\mathfrak {g}}_i,{\mathfrak {g}}_j]\subseteq {\mathfrak {g}}_{i+j}$ for all *i*, *j*, and the subalgebra ${\mathfrak {g}}_-:={\mathfrak {g}}_{-k}\oplus \cdots \oplus {\mathfrak {g}}_{-1}$ is generated by ${\mathfrak {g}}_{-1}$, see [10, Definition 3.1.2]. Consider the filtration

88 $$\begin{aligned} {\mathfrak {g}}={\mathfrak {g}}^{-k}\supseteq {\mathfrak {g}}^{-k+1}\supseteq \cdots \supseteq {\mathfrak {g}}^{-1}\supseteq {\mathfrak {g}}^0\supseteq {\mathfrak {g}}^1\supseteq \cdots \supseteq {\mathfrak {g}}^{k-1}\supseteq {\mathfrak {g}}^k \end{aligned}$$

where ${\mathfrak {g}}^i:={\mathfrak {g}}_i\oplus \cdots \oplus {\mathfrak {g}}_k$. The subalgebras ${\mathfrak {g}}_0$ and ${\mathfrak {p}}:={\mathfrak {g}}^0={\mathfrak {g}}_0\oplus \cdots \oplus {\mathfrak {g}}_k$ can be characterized as grading and filtration-preserving subalgebras, respectively. More precisely, ${\mathfrak {g}}_0=\{X\in {\mathfrak {g}}\mid \forall i:{{\,\mathrm{ad}\,}}_X({\mathfrak {g}}_i)\subseteq {\mathfrak {g}}_i\}$ and ${\mathfrak {p}}=\{X\in {\mathfrak {g}}\mid \forall i:{{\,\mathrm{ad}\,}}_X({\mathfrak {g}}^i)\subseteq {\mathfrak {g}}^i\}$, see [10, Lemma 3.1.3(1)]. Also note that ${\mathfrak {p}}_+:={\mathfrak {g}}^1={\mathfrak {g}}_1\oplus \cdots \oplus {\mathfrak {g}}_k$ is a nilpotent ideal in ${\mathfrak {p}}$.

Let *G* be a not necessarily connected Lie group with Lie algebra ${\mathfrak {g}}$. Then

89 $$\begin{aligned} \{g\in G\mid \forall i:{{\,\mathrm{Ad}\,}}_g({\mathfrak {g}}^i)\subseteq {\mathfrak {g}}^i\} \end{aligned}$$

is a closed subgroup of *G* with Lie algebra ${\mathfrak {p}}$, see [10, Lemma 3.1.3(2)]. Let *P* be a parabolic subgroup of *G* corresponding to the |*k*|-grading (87), i.e., a subgroup between (89) and its connected component, see [10, Definition 3.1.3]. Hence, *P* has Lie algebra ${\mathfrak {p}}$, and the corresponding Levi subgroup,

$$\begin{aligned} G_0:=\{g\in P\mid \forall i:{{\,\mathrm{Ad}\,}}_g({\mathfrak {g}}_i)\subseteq {\mathfrak {g}}_i\}, \end{aligned}$$

has Lie algebra ${\mathfrak {g}}_0$. According to [10, Theorem 3.1.3] we have a diffeomorphism

90 $$\begin{aligned} G_0\times {\mathfrak {p}}_+\cong P,\qquad (g,X)\mapsto g\exp (X). \end{aligned}$$

In particular, $P_+:=\exp ({\mathfrak {p}}_+)$ is a closed normal nilpotent subgroup of *P*, and the inclusion $G_0\subseteq P$ induces a canonical isomorphism $G_0=P/P_+$. Note that $P_+$ acts trivially on the associated graded, ${{\,\mathrm{gr}\,}}({\mathfrak {g}})$, of the filtration (88). In particular, ${{\,\mathrm{gr}\,}}({\mathfrak {g}}/{\mathfrak {p}})$ can be considered as a representation of $P/P_+=G_0$. The inclusion ${\mathfrak {g}}_-\subseteq {\mathfrak {g}}$ induces a canonical isomorphism of $G_0$-modules,

91 $$\begin{aligned} {\mathfrak {g}}_-={{\,\mathrm{gr}\,}}({\mathfrak {g}}/{\mathfrak {p}}). \end{aligned}$$

A parabolic geometry of type (*G*, *P*) consists of a principal *P*-bundle $p:{\mathcal {G}}\rightarrow M$ and a Cartan connection $\omega \in \Omega ^1({\mathcal {G}};{\mathfrak {g}})$, see [10, Definition 3.1.4 and Section 1.5]. Hence, the ${\mathfrak {g}}$-valued 1-form $\omega$ provides a *P*-equivariant trivialization of the tangent bundle $T{\mathcal {G}}$, that is, $\omega _u:T_u{\mathcal {G}}\rightarrow {\mathfrak {g}}$ is a linear isomorphism for each $u\in {\mathcal {G}}$, and $(r^g)^*\omega ={{\,\mathrm{Ad}\,}}_{g^{-1}}\omega$ for all $g\in P$, where $r^g$ denotes the principal right action of $g\in P$ on ${\mathcal {G}}$. Moreover, $\omega$ reproduces the generators of the right *P*-action, i.e., for all $X\in {\mathfrak {p}}$, we have $\omega (\zeta _X)=X$ where $\zeta _X:=\frac{d}{dt}|_0r^{\exp (tX)}$ denotes the fundamental vector field. The prototypical example of a parabolic geometry of type (*G*, *P*) is its flat model, that is, the generalized flag variety *G*/*P* with the canonical projection $G\rightarrow G/P$ and the Maurer–Cartan form on *G*.

Using the Cartan connection, the filtration (88) provides a filtration of the tangent bundle $T{\mathcal {G}}$ by *P*-invariant subbundles,

$$\begin{aligned} T{\mathcal {G}}=T^{-k}{\mathcal {G}}\supseteq T^{-k+1}{\mathcal {G}}\supseteq \cdots \supseteq T^k{\mathcal {G}}, \end{aligned}$$

where $T^i_u{\mathcal {G}}:=\omega _u^{-1}({\mathfrak {g}}^i)$ for $u\in {\mathcal {G}}$. Note that $T^0{\mathcal {G}}$ coincides with the vertical bundle of the projection $p:{\mathcal {G}}\rightarrow M$. Hence, there exists a unique filtration of *TM* by subbundles,

92 $$\begin{aligned} TM=T^{-k}M\supseteq T^{-k+1}M\supseteq \cdots \supseteq T^{-1}M, \end{aligned}$$

such that $(Tp)^{-1}(T^iM)=T^i{\mathcal {G}}$. The Cartan connection induces an isomorphism

93 $$\begin{aligned} TM\cong {\mathcal {G}}\times _P({\mathfrak {g}}/{\mathfrak {p}}) \end{aligned}$$

intertwining the filtration (92) with the filtration induced from (88). Using (91) for the associated graded we obtain an isomorphism of vector bundles,

94 $$\begin{aligned} {{\,\mathrm{gr}\,}}(TM)\cong {\mathcal {G}}_0\times _{G_0}{\mathfrak {g}}_-. \end{aligned}$$

Here ${\mathcal {G}}_0:={\mathcal {G}}/P_+$ is considered as a principal $G_0$-bundle over *M*.

We assume that the Cartan connection $\omega$ is regular, see [10, Definition 3.1.7]. Hence, the filtration on *TM*, see (92), turns *M* into a filtered manifold, and the corresponding Levi bracket ${{\,\mathrm{gr}\,}}(TM)\otimes {{\,\mathrm{gr}\,}}(TM)\rightarrow {{\,\mathrm{gr}\,}}(TM)$ induced by the Lie bracket of vector fields coincides with the algebraic bracket induced by the Lie bracket ${\mathfrak {g}}_-\otimes {\mathfrak {g}}_-\rightarrow {\mathfrak {g}}_-$ via (94). In other words, using the notation from Sect. 2.1, the Cartan connection of a regular parabolic geometry provides an isomorphism of bundles of graded nilpotent Lie algebras

95 $$\begin{aligned} \mathfrak tM\cong {\mathcal {G}}_0\times _{G_0}{\mathfrak {g}}_-. \end{aligned}$$

Recall that the Cartan connection induces a principal connection on ${\mathcal {P}}\times _PG$. More precisely, there exists a unique principal connection on the principal *G*-bundle ${\mathcal {G}}\times _PG\rightarrow M$ which restricts to the Cartan connection $\omega$ along the inclusion ${\mathcal {G}}\subseteq {\mathcal {G}}\times _PG$, see [10, Theorem 1.5.6]. Consequently, for every finite dimensional *G*-representation ${\mathbb {E}}$, the Cartan connection induces a linear connection $\nabla$ on the associated tractor bundle

$$\begin{aligned} E:={\mathcal {G}}\times _P{\mathbb {E}}=({\mathcal {G}}\times _PG)\times _G{\mathbb {E}}. \end{aligned}$$

Recall that ${\mathbb {E}}$ admits a grading, ${\mathbb {E}}={\mathbb {E}}_{-l}\oplus \cdots \oplus {\mathbb {E}}_l$, which is compatible with the grading of ${\mathfrak {g}}$, i.e., $X\cdot v\in {\mathbb {E}}_{i+j}$ for all $X\in {\mathfrak {g}}_i$ and $X\in {\mathbb {E}}_j$. Indeed, there exists a unique grading element in ${\mathfrak {g}}$ which acts by multiplication with *j* on the component ${\mathfrak {g}}_j$, see [10, Proposition 3.1.2(1)], and the eigenspaces of its action on ${\mathbb {E}}$ provide the desired decomposition. The grading of ${\mathbb {E}}$ is $G_0$-invariant since the uniqueness of the grading element implies that it is stabilized by $G_0$. Hence, the associated filtration ${\mathbb {E}}^i:=\bigoplus _{j\ge i}{\mathbb {E}}_j$ is *P*-invariant, see (90). Moreover, $P_+$ acts trivially on the associated graded, and ${{\,\mathrm{gr}\,}}({\mathbb {E}})={\mathbb {E}}$ as representations of $P/P_+=G_0$. The *P*-invariant filtration of ${\mathbb {E}}$ induces a filtration of *E* by subbundles $E^i:={\mathcal {G}}\times _P{\mathbb {E}}^i$. Clearly, the linear connection $\nabla$ is filtration-preserving, that is, for all $X\in \Gamma ^\infty (T^pM)$ and $\psi \in \Gamma ^\infty (E^q)$ we have $\nabla _X\psi \in \Gamma ^\infty (E^{p+q})$. Since the Cartan connection is assumed to be regular, its curvature $F^\nabla \in \Omega ^2(M;{{\,\mathrm{end}\,}}(E))$ is contained in filtration degree one, that is, for all $X_i\in \Gamma ^\infty (T^{p_i}M)$ and $\psi \in \Gamma ^\infty (E^q)$ we have $F^\nabla (X_1,X_2)\psi \in \Gamma ^\infty (E^{p_1+p_2+q+1})$, see [10, Corollary 3.1.8(2) and Theorem 3.1.22(3)]. The isomorphism (93) induces an isomorphism

96 $$\begin{aligned} \Lambda ^kT^*M\otimes E\cong {\mathcal {G}}\times _P(\Lambda ^k({\mathfrak {g}}/{\mathfrak {p}})^*\otimes {\mathbb {E}}) \end{aligned}$$

which intertwines the filtration on $\Lambda ^kT^*M\otimes E$ with the one induced from the filtration on $\Lambda ^k({\mathfrak {g}}/{\mathfrak {p}})^*\otimes {\mathbb {E}}$. Moreover, (91) provides an isomorphism of $G_0$-modules,

97 $$\begin{aligned} {{\,\mathrm{gr}\,}}\bigl (\Lambda ^k({\mathfrak {g}}/{\mathfrak {p}})^*\otimes {\mathbb {E}}\bigr )=C^k({\mathfrak {g}}_-;{\mathbb {E}}), \end{aligned}$$

where $C^k({\mathfrak {g}}_-;{\mathbb {E}}):=\Lambda ^k{\mathfrak {g}}_-^*\otimes {\mathbb {E}}$. Hence, (96) induces an isomorphism

98 $$\begin{aligned} {{\,\mathrm{gr}\,}}\bigl (\Lambda ^kT^*M\otimes E\bigr ) \cong {\mathcal {G}}_0\times _{G_0}C^k({\mathfrak {g}}_-;{\mathbb {E}}). \end{aligned}$$

The extension $d^\nabla :\Omega ^k(M;E)\rightarrow \Omega ^{k+1}(M;E)$ characterized by the Leibniz rule, see (69), is filtration-preserving, and via (98) we have

99 $$\begin{aligned} {{\,\mathrm{gr}\,}}(d^\nabla )={\mathcal {G}}_0\times _{G_0}\partial _{{\mathfrak {g}}_-} \end{aligned}$$

where $\partial _{{\mathfrak {g}}_-}:C^k({\mathfrak {g}}_-;{\mathbb {E}})\rightarrow C^{k+1}({\mathfrak {g}}_-;{\mathbb {E}})$ denotes the differential in the standard complex computing Lie algebra cohomology $H^*({\mathfrak {g}}_-;{\mathbb {E}})$, see Lemma 4.14.

Let $\delta _{{\mathfrak {p}}_+}:\Lambda ^k{\mathfrak {p}}_+\otimes {\mathbb {E}}\rightarrow \Lambda ^{k-1}{\mathfrak {p}}_+\otimes {\mathbb {E}}$ denote the differential in the standard complex computing the Lie algebra homology $H_*({\mathfrak {p}}_+;{\mathbb {E}})$ with coefficients in ${\mathbb {E}}$. Since $\delta _{{\mathfrak {p}}_+}$ is *P*-equivariant, and since the Killing form provides an isomorphism of *P*-modules $({\mathfrak {g}}/{\mathfrak {p}})^*\cong {\mathfrak {p}}_+$, the differential $\delta _{{\mathfrak {p}}_+}$ dualizes to a *P*-equivariant map

100 $$\begin{aligned} \delta _{{\mathfrak {g}}/{\mathfrak {p}}}:\Lambda ^k({\mathfrak {g}}/{\mathfrak {p}})^*\otimes {\mathbb {E}}\rightarrow \Lambda ^{k-1}({\mathfrak {g}}/{\mathfrak {p}})^*\otimes {\mathbb {E}}. \end{aligned}$$

Via the identification (96), it gives rise to a vector bundle homomorphism,

101 $$\begin{aligned} \delta :\Lambda ^kT^*M\otimes E\rightarrow \Lambda ^{k-1}T^*M\otimes E,\qquad \delta :={\mathcal {G}}\times _P\delta _{{\mathfrak {g}}/{\mathfrak {p}}}. \end{aligned}$$

In the literature [10–12] this homomorphism is often denoted $\partial ^*$. Clearly, $\delta ^2=0$. Moreover, $\delta$ is filtration-preserving and via (98)

102 $$\begin{aligned} {{\,\mathrm{gr}\,}}(\delta )={\mathcal {G}}_0\times _{G_0}\delta _{{\mathfrak {g}}_-}, \end{aligned}$$

where $\delta _{{\mathfrak {g}}_-}:C^k({\mathfrak {g}}_-;{\mathbb {E}})\rightarrow C^{k-1}({\mathfrak {g}}_-;{\mathbb {E}})$ is obtained from (100) by passing to the associated graded and using the identification (97), that is, $\delta _{{\mathfrak {g}}_-}={{\,\mathrm{gr}\,}}(\delta _{{\mathfrak {g}}/{\mathfrak {p}}})$. Actually, $\delta _{{\mathfrak {g}}/{\mathfrak {p}}}$ is grading preserving, hence $\delta _{{\mathfrak {g}}_-}=\delta _{{\mathfrak {g}}/{\mathfrak {p}}}$ via the isomorphism of $G_0$-modules

103 $$\begin{aligned} \Lambda ^k{\mathfrak {g}}_-^*\otimes {\mathbb {E}}=\Lambda ^k({\mathfrak {g}}/{\mathfrak {p}})^*\otimes {\mathbb {E}} \end{aligned}$$

induced by the identification ${\mathfrak {g}}_-={\mathfrak {g}}/{\mathfrak {p}}$.

Recall that a Weyl structure is a $G_0$-equivariant section of the principal $P_+$-bundle ${\mathcal {G}}\rightarrow {\mathcal {G}}_0={\mathcal {G}}/P_+$, see [10, Definition 5.1.1]. Global Weyl structures always exist, see [10, Proposition 5.1.1]. Moreover, the (contractible) space of sections of the bundle of groups ${\mathcal {G}}_0\times _{G_0}P_+$ acts free and transitively on the space of Weyl structures. Using the isomorphism of $G_0$-modules (103), every Weyl structure ${\mathcal {G}}_0\rightarrow {\mathcal {G}}$ induces a filtration-preserving isomorphism of vector bundles,

$$\begin{aligned} {\mathcal {G}}_0\times _{G_0}C^k({\mathfrak {g}}_-;{\mathbb {E}})\xrightarrow \cong {\mathcal {G}}\times _P\bigl ((\Lambda ^k({\mathfrak {g}}/{\mathfrak {p}})^*\otimes {\mathbb {E}}\bigr ), \end{aligned}$$

inducing the identity on the associated graded, see (97). Via (96) and (98) this corresponds to a splitting of the filtration

$$\begin{aligned} S:{{\,\mathrm{gr}\,}}(\Lambda ^kT^*M\otimes E)\rightarrow \Lambda ^kT^*M\otimes E \end{aligned}$$

satisfying $$\delta \circ S=S\circ {{\,\mathrm{gr}\,}}(\delta )$$.

Kostant [51] observed that $\delta _{{\mathfrak {g}}_-}$ and $\partial _{{\mathfrak {g}}_-}$ are adjoint with respect to positive definite inner products on the spaces $C^k({\mathfrak {g}}_-;{\mathbb {E}})$, see [10, Proposition 3.1.1]. Hence, the Laplacian

$$\begin{aligned} \Box _{{\mathfrak {g}}_-}:C^*({\mathfrak {g}}_-;{\mathbb {E}})\rightarrow C^*({\mathfrak {g}}_-;{\mathbb {E}}),\qquad \Box _{{\mathfrak {g}}_-}:=\delta _{{\mathfrak {g}}_-}\circ \partial _{{\mathfrak {g}}_-}+\partial _{{\mathfrak {g}}_-}\circ \delta _{{\mathfrak {g}}_-}, \end{aligned}$$

gives rise to a finite dimensional Hodge decomposition

104 $$\begin{aligned} C^*({\mathfrak {g}}_-;{\mathbb {E}})={{\,\mathrm{img}\,}}(\delta _{{\mathfrak {g}}_-})\oplus \ker (\Box _{{\mathfrak {g}}_-})\oplus {{\,\mathrm{img}\,}}(\partial _{{\mathfrak {g}}_-}). \end{aligned}$$

Using (99) and (102) we see that $\Box :\Omega ^*(M;E)\rightarrow \Omega ^*(M;E)$, $\Box =\delta \circ d^\nabla +d^\nabla \circ \delta$, is filtration-preserving, and via the identification (98) we have

105 $$\begin{aligned} {{\,\mathrm{gr}\,}}(\Box )={\mathcal {G}}_0\times _{G_0}\Box _{{\mathfrak {g}}_-}. \end{aligned}$$

For the fiber-wise projection ${\tilde{P}}:{{\,\mathrm{gr}\,}}(\Lambda ^kT^*M\otimes E)\rightarrow {{\,\mathrm{gr}\,}}(\Lambda ^kT^*M\otimes E)$ onto the (generalized) zero eigenspace of ${{\,\mathrm{gr}\,}}(\Box )$, we obtain, via (98),

$$\begin{aligned} {\tilde{P}}={\mathcal {G}}_0\times _{G_0}P_{{\mathfrak {g}}_-} \end{aligned}$$

where $P_{{\mathfrak {g}}_-}:C^*({\mathfrak {g}}_-;{\mathbb {E}})\rightarrow C^*({\mathfrak {g}}_-;{\mathbb {E}})$ denotes the projection onto $\ker (\Box _{{\mathfrak {g}}_-})$ along the decomposition (104). In particular, ${{\,\mathrm{gr}\,}}(\delta )\circ {\tilde{P}}=0$.

From the discussion above we conclude that the homomorphism $\delta$, see (101), is a Kostant type codifferential of maximal rank for the linear connection $\nabla$ on the tractor bundle *E*, see Definition 4.6 and Remark 4.11. In this situation Corollary 4.18(a) reduces to the statement in [12, Theorem 2.4], see also [8, 11]. Since $P_+$ acts trivially on $H_*({\mathfrak {p}}_+;{\mathbb {E}})$ the *P*-action on $H_*({\mathfrak {p}}_+;{\mathbb {E}})$ factors to an action by $P/P_+=G_0$ and we have canonical identifications:

$$\begin{aligned} {\mathcal {H}}_* ={\mathcal {G}}_0\times _{G_0}H_*({\mathfrak {p}}_+;{\mathbb {E}}) ={\mathcal {G}}_0\times _{G_0}\ker (\Box _{{\mathfrak {g}}_-}) ={\mathcal {G}}_0\times _{G_0}H^*({\mathfrak {g}}_-;{\mathbb {E}}). \end{aligned}$$

The corresponding sequence of differential operators in Corollary 4.18(b) coincides with one version of (curved) BGG sequences that can be found in the literature. This is called “torsion free BGG sequence” in [8, Section 5] and coincides with the sequence constructed in [12, Section 2.4]. For torsion free parabolic geometries, see [10, Section 1.5.7], this coincides with the original curved BGG sequence constructed by Čap et al. [11]. From Corollary 4.18(b) we thus obtain

#### Corollary 4.20

The (torsion free) BGG sequence associated to a regular parabolic geometry of type (*G*, *P*) and a finite dimensional *G*-representation is a graded Rockland sequence of differential operators which have graded Heisenberg order at most zero.

Kostant’s version of the Bott–Borel–Weil theorem permits us to effectively compute the homologies $H_k({\mathfrak {p}}_+;{\mathbb {E}})$ as modules over ${\mathfrak {g}}_0$. More precisely, $H_k({\mathfrak {p}}_+;{\mathbb {E}})$ decomposes as a direct sum of irreducible ${\mathfrak {g}}_0$-modules whose dominant weights can be read off the Hasse diagram of ${\mathfrak {p}}$, see [51] or [10, Theorem 3.3.5 and Proposition 3.3.6]. Moreover, the grading element acts as a scalar on each of these irreducible components which can easily be computed too, see [10, Section 3.2.12]. Consequently, representation theory permits us to determine the decomposition according to the grading $H_k({\mathfrak {p}}_+;{\mathbb {E}})=\bigoplus _pH_k({\mathfrak {p}}_+;{\mathbb {E}})_p$. Decomposing the BGG operators accordingly, ${\bar{D}}_k=\sum _{p,q}({\bar{D}}_k)_{qp}$, with

106 $$\begin{aligned} \Gamma ^\infty \bigl ({\mathcal {G}}_0\times _{G_0}H_k({\mathfrak {p}}_+;{\mathbb {E}})_p\bigr ) \xrightarrow {({\bar{D}}_k)_{qp}} \Gamma ^\infty \bigl ({\mathcal {G}}_0\times _{G_0}H_{k+1}({\mathfrak {p}}_+;{\mathbb {E}})_q\bigr ), \end{aligned}$$

one part of Corollary 4.20 asserts that the differential operator in (106) is of Heisenberg order at most $q-p$, a statement which appears to be well known. Using a frame $({\mathcal {G}}_0)_x\cong G_0$ at $x\in M$, the Heisenberg principal symbol of the operator (106) at *x* can be regarded as a left invariant differential operator which is homogeneous of order $q-p$,

$$\begin{aligned} C^\infty \bigl ({\mathcal {T}}_xM,H_k({\mathfrak {p}}_+;{\mathbb {E}})_p\bigr )\xrightarrow {\sigma ^{q-p}_x(({\bar{D}}_k)_{qp})}C^\infty \bigl ({\mathcal {T}}_xM,H_{k+1}({\mathfrak {p}}_+;{\mathbb {E}})_q\bigr ). \end{aligned}$$

The second part of Corollary 4.20 asserts that these Heisenberg principal symbols combine to form a sequence of left invariant differential operators

107 $$\begin{aligned} \cdots \rightarrow C^\infty \bigl ({\mathcal {T}}_xM,H_k({\mathfrak {p}}_+;{\mathbb {E}})\bigr ) \xrightarrow {{\tilde{\sigma }}^0_x({\bar{D}}_k)} C^\infty \bigl ({\mathcal {T}}_xM,H_{k+1}({\mathfrak {p}}_+;{\mathbb {E}})\bigr )\rightarrow \cdots \end{aligned}$$

where ${\tilde{\sigma }}_x^0({\bar{D}}_k)=\sum _{p,q}\sigma ^{q-p}_x(({\bar{D}}_k)_{qp})$, which is Rockland in the sense that it becomes exact in every non-trivial irreducible unitary representation of ${\mathcal {T}}_xM$. Up to the isomorphism ${\mathcal {T}}_xM\cong G_-:=\exp ({\mathfrak {g}}_-)$ provided by the frame, the graded Heisenberg principal symbol of a BGG operator in (107) coincides with the corresponding BGG operator on the flat model *G*/*P* restricted along the local diffeomorphism $G_-\rightarrow G/P$ obtained from the inclusion $G_-\subseteq G$.

If the homology $H_k({\mathfrak {p}}_+;{\mathbb {E}})$ is concentrated in a single degree for each *k*, that is, if there exist numbers $p_k$ such that $H_k({\mathfrak {p}}_+;{\mathbb {E}})=H_k({\mathfrak {p}}_+;{\mathbb {E}})_{p_k}$, then the corresponding BGG operator ${\bar{D}}_k:\Gamma ^\infty ({\mathcal {H}}_k)\rightarrow \Gamma ^\infty ({\mathcal {H}}_{k+1})$ is of Heisenberg order at most $p_{k+1}-p_k$ and the BGG sequence is Rockland in the ungraded sense, see Definition 2.10. If, moreover, $p_{k+1}-p_k\ge 1$, then the analytic results established in the preceding sections are applicable, see, in particular, Corollaries 2.15, 2.16, 3.24, and 3.25. Below we will discuss a classical example of this type.

#### Example 4.21

(*Generic rank two distributions in dimension five*) Let *M* be a 5-manifold equipped with a rank two distribution of Cartan type, $T^{-1}M\subseteq TM$, see [7, 14, 71]. Hence, $T^{-1}M$ is a rank two subbundle of *TM* with growth vector (2, 3, 5), that is, Lie brackets of sections of $T^{-1}M$ span a rank three subbundle $T^{-2}M$ of *TM* and triple brackets of sections of $T^{-1}M$ span all of *TM*. These geometric structures are also known as generic rank two distributions in dimension five, see [9, 71]. The topological obstructions to global existence of such a distribution are well understood in the orientable case, see [23, Theorem 1]. On open 5-manifolds, Gromov’s h-principle is applicable and establishes existence, once the topological requirements are met, see [23, Theorem 2]. It is unclear, however, if there are further geometric obstructions on closed 5-manifolds. Whether rank two distributions of Cartan type also abide by an h-principle on closed manifolds, appears to be an intriguing open question and is a major motivation for our investigation of hypoelliptic sequences.

In his celebrated paper [14] Cartan has shown that, up to isomorphism, there exists a unique regular normal parabolic geometry of type (*G*, *P*) on *M* with underlying filtration:

$$\begin{aligned} TM=T^{-3}M\supseteq T^{-2}M\supseteq T^{-1}M\supseteq T^0M=0. \end{aligned}$$

Here *G* denotes the split real form of the exceptional Lie group $G_2$ and *P* denotes the maximal parabolic subgroup corresponding to the shorter simple root. Hence, every finite dimensional representation ${\mathbb {E}}$ of *G* gives rise to a curved BGG sequence on *M*,

108 $$\begin{aligned} \Gamma ^\infty ({\mathcal {H}}_0)\xrightarrow {{\bar{D}}_0} \Gamma ^\infty ({\mathcal {H}}_1)\xrightarrow {{\bar{D}}_1} \Gamma ^\infty ({\mathcal {H}}_2)\xrightarrow {{\bar{D}}_2} \Gamma ^\infty ({\mathcal {H}}_3)\xrightarrow {{\bar{D}}_3} \Gamma ^\infty ({\mathcal {H}}_4)\xrightarrow {{\bar{D}}_4} \Gamma ^\infty ({\mathcal {H}}_5), \end{aligned}$$

where ${\mathcal {H}}_k:={\mathcal {G}}_0\times _{G_0}H_k({\mathfrak {p}}_+;{\mathbb {E}})$. We label the longer simple root of the $G_2$ root system by $\alpha _1$ and let $\alpha _2$ denote the shorter simple root. Hence, $2\alpha _1+3\alpha _2$ is the highest root, and the corresponding fundamental weights are $\lambda _1=2\alpha _1+3\alpha _2$ and $\lambda _2=\alpha _1+2\alpha _2$. Suppose ${\mathbb {E}}$ is the irreducible complex representation with highest weight $a\lambda _1+b\lambda _2$ where $a,b\in {\mathbb {N}}_0$. Then $H_k({\mathfrak {p}}_+;{\mathbb {E}})$ is an irreducible complex module of ${\mathfrak {g}}_0\subseteq {\mathfrak {g}}_0^{\mathbb {C}}\cong {\mathfrak {g}}{\mathfrak {l}}_2({\mathbb {C}})$ which can readily be determined by working out the Hasse diagram of ${\mathfrak {p}}^{\mathbb {C}}$, see [10, Section 3.2.16], and using Kostant’s version of the Bott–Borel–Weil theorem, see [10, Theorem 3.3.5 and Proposition 3.3.6]. Denoting the highest weight of $H_k({\mathfrak {p}}_+;{\mathbb {E}})$ by $a_k\lambda _1+b_k\lambda _2$, we obtain the first three columns in the following table:^7^109The grading element acts by multiplication with the scalar $p_k=3a_k+2b_k$ on $H_k({\mathfrak {p}}_+;{\mathbb {E}})$, see [10, Section 3.2.12], whence the last column. Moreover, the highest weight of $H_k({\mathfrak {p}}_+;{\mathbb {E}})$ considered as ${\mathfrak {s}}{\mathfrak {l}}_2({\mathbb {C}})$-module is $a_k$ times the fundamental weight of ${\mathfrak {s}}{\mathfrak {l}}_2({\mathbb {C}})$, hence $\dim H_k({\mathfrak {p}}_+;{\mathbb {E}})=a_k+1$, whence the remaining column. Since ${\bar{D}}_k$ is of Heisenberg order $p_{k+1}-p_k$, we conclude that ${\bar{D}}_0$ and ${\bar{D}}_4$ are of Heisenberg order $b+1$; ${\bar{D}}_1$ and ${\bar{D}}_3$ are of Heisenberg order $3(a+1)$; and ${\bar{D}}_2$ is of Heisenberg order $2(b+1)$. According to Corollary 4.20 the sequence (108) is Rockland in the ungraded sense, see Definition 2.10. In particular, the differential operators ${\bar{D}}_0^*{\bar{D}}_0$, $({\bar{D}}_0{\bar{D}}_0^*)^{3(a+1)}+({\bar{D}}_1^*{\bar{D}}_1)^{b+1}$, $({\bar{D}}_1{\bar{D}}_1^*)^{2(b+1)}+({\bar{D}}_2^*{\bar{D}}_2)^{3(a+1)}$, $({\bar{D}}_2{\bar{D}}_2^*)^{3(a+1)}+({\bar{D}}_3^*{\bar{D}}_3)^{2(b+1)}$, $({\bar{D}}_3{\bar{D}}_3^*)^{b+1}+({\bar{D}}_4^*{\bar{D}}_4)^{3(a+1)}$, and ${\bar{D}}_4{\bar{D}}_4^*$ are all hypoelliptic and maximal hypoelliptic estimates are available, see Lemma 2.14, Theorem 3.11, as well as, Corollaries 3.12, 3.20, and 3.22. Here ${\bar{D}}_k^*$ denotes the formal adjoint of ${\bar{D}}_k$ with respect to any fiber-wise Hermitian metrics on the vector bundles ${\mathcal {H}}_k$ and any volume density on *M*.

Let us finally put down explicit formulas for the Heisenberg principal symbol of the BGG operators corresponding to the trivial representation ${\mathbb {E}}$. We consider these operators as left invariant differential operators on the simply connected nilpotent Lie group $G_-$. According to the discussion above, this BGG sequence has the form

110 $$\begin{aligned} C^\infty (G_-)\xrightarrow {D_0} C^\infty (G_-)^2\xrightarrow {D_1} C^\infty (G_-)^3\xrightarrow {D_2} C^\infty (G_-)^3\xrightarrow {D_3} C^\infty (G_-)^2\xrightarrow {D_4} C^\infty (G_-)\nonumber \\ \end{aligned}$$

where $D_0$ and $D_4$ are homogeneous of degree 1; $D_1$ and $D_3$ are homogeneous of degree 3; and $D_2$ is homogeneous of degree 2, see also [6]. Using matrices with entries in the universal enveloping algebra of ${\mathfrak {g}}_-$ these operators can be expressed as:

$$\begin{aligned} D_0= & {} \left( \begin{array}{c} X_1\\ X_2 \end{array}\right) \\ D_1= & {} \left( \begin{array}{cc} -X_4-X_{112}-X_{13}&{}X_{111}\\ -X_5-X_{122}&{}X_{112}-2X_{13}\\ -X_{222}&{}X_{122}-3X_{23} \end{array}\right) \\ D_2= & {} \left( \begin{array}{ccc} -X_{12}-X_3&{}X_{11}&{} 0\\ -X_{22}&{}-3X_3&{} X_{11}\\ 0&{} -X_{22}&{}X_{12}-2X_3\\ \end{array}\right) \\ D_3= & {} \left( \begin{array}{ccc} X_{122}+X_{23}-2X_5&{}-X_{112}+X_4&{}X_{111}\\ X_{222}&{}-X_{122}+2X_{32}&{}X_{112}-3X_{13}+3X_4 \end{array}\right) \\ D_4= & {} \left( \begin{array}{cc} -X_2&X_1 \end{array}\right) \end{aligned}$$

Here $X_5,X_4|X_3|X_2,X_1$ is a graded basis of ${\mathfrak {g}}_-={\mathfrak {g}}_{-3}\oplus {\mathfrak {g}}_{-2}\oplus {\mathfrak {g}}_{-1}$ such that

$$\begin{aligned}{}[X_1,X_2]=X_3,\quad [X_1,X_3]=X_4,\quad [X_2,X_3]=X_5. \end{aligned}$$

The vertical bars above indicate that $X_1,X_2$ is a basis of ${\mathfrak {g}}_{-1}$, $X_3$ is a basis of ${\mathfrak {g}}_{-2}$, and $X_4,X_5$ is a basis of ${\mathfrak {g}}_{-3}$. Moreover, we use the notation $X_{i_1\dotsc i_k}=X_{i_1}\cdots X_{i_k}$. These formulas are derived in [22, Appendix B].

## Graded hypoelliptic analysis

In this section, we adapt the analysis discussed in Sect. 3 to the filtered setup required to deal with the sequences constructed in Sect. 4. Everything generalizes effortlessly, but one bit: Formal adjoints of graded (pseudo)differential operators are in general only available if the underlying manifold is closed. This is related to the fact that we can construct invertible $\Lambda _s\in \Psi ^s(E)$ with $\Lambda _s^{-1}\in \Psi ^{-s}(E)$ only on closed manifolds, see Lemma 3.13 and (117).

### Graded pseudodifferential operators

The concept of graded Heisenberg order for differential operators introduced in Sect. 4.1 can be generalized to pseudodifferential operators in a straight forward manner as follows. Let *E* and *F* be two filtered vector bundles over a filtered manifold *M*, suppose $A\in {\mathcal {O}}(E,F)$, and let *s* be a complex number. Choose splittings of the filtrations, $S_E:{{\,\mathrm{gr}\,}}(E)\rightarrow E$ and $S_F:{{\,\mathrm{gr}\,}}(F)\rightarrow F$ and decompose the operator accordingly, $S_F^{-1}AS_E=\sum _{q,p}(S_F^{-1}AS_E)_{q,p}$, where $(S_F^{-1}AS_E)_{q,p}\in {\mathcal {O}}({{\,\mathrm{gr}\,}}_p(E),{{\,\mathrm{gr}\,}}_q(F))$. We say *A* has *graded Heisenberg order* *s* if $(S_F^{-1}AS_E)_{q,p}\in \Psi ^{s+q-p}({{\,\mathrm{gr}\,}}_p(E),{{\,\mathrm{gr}\,}}_q(F))$ for all *p* and *q*. We let ${\tilde{\Psi }}^s(E,F)$ denote the space of pseudodifferential operators of graded Heisenberg order *s*. One readily checks that this space does not depend on the choice of splittings $S_E$ and $S_F$.

Let us define the *space of principal cosymbols of graded order* *s* by

$$\begin{aligned} {\tilde{\Sigma }}^s(E,F) :=\left\{ k\in \frac{{\mathcal {K}} (\mathcal {T}M;{{\,\mathrm{gr}\,}}(E),{{\,\mathrm{gr}\,}}(F))}{{\mathcal {K}}^\infty (\mathcal {T}M; {{\,\mathrm{gr}\,}}(E),{{\,\mathrm{gr}\,}}(F))}: (\delta _\lambda )_*k=\lambda ^s\delta _\lambda ^Fk\delta ^E_{1/\lambda }\text { for all }\lambda >0\right\} , \end{aligned}$$

where $\delta ^E_\lambda \in {{\,\mathrm{Aut}\,}}({{\,\mathrm{gr}\,}}(E))$ denotes the automorphism given by multiplication with $\lambda ^p$ on the grading component ${{\,\mathrm{gr}\,}}_p(E)$. These are essentially homogeneous kernels in a graded sense, taking the grading on ${{\,\mathrm{gr}\,}}(E)$ and ${{\,\mathrm{gr}\,}}(F)$ into account. They can be canonically identified with matrices of ordinary principal cosymbols,

111 $$\begin{aligned} {\tilde{\Sigma }}^s(E,F)=\bigoplus _{q,p}\Sigma ^{s+q-p}({{\,\mathrm{gr}\,}}_p(E),{{\,\mathrm{gr}\,}}_q(F)). \end{aligned}$$

For $A\in {\tilde{\Psi }}^s(E,F)$ we define the *graded Heisenberg principal cosymbol* ${\tilde{\sigma }}^s(A)\in {\tilde{\Sigma }}^s(E,F)$ by ${\tilde{\sigma }}^s(A):=\sum _{p,q}\sigma ^{s+q-p}\bigl ((S_F^{-1}AS_E)_{q,p}\bigr )$ where $\sigma ^{s+q-p}((S_F^{-1}AS_E)_{q,p})\in \Sigma ^s({{\,\mathrm{gr}\,}}_p(E),{{\,\mathrm{gr}\,}}_q(F))$ are the Heisenberg principal symbols of the components. One readily checks that the graded principal Heisenberg cosymbol is independent of the choice of splittings $S_E$ and $S_F$. From Proposition 3.1(b) we immediately obtain a short exact sequence:

$$\begin{aligned} 0\rightarrow {\tilde{\Psi }}^{s-1}(E,F)\rightarrow {\tilde{\Psi }}^s(E,F)\xrightarrow {{\tilde{\sigma }}^s} {\tilde{\Sigma }}^s(E,F)\rightarrow 0. \end{aligned}$$

If $A\in {\tilde{\Psi }}^s(E,F)$ and $B\in {\tilde{\Psi }}^r(F,G)$, then $BA\in {\tilde{\Psi }}^{r+s}(E,G)$ and

112 $$\begin{aligned} {\tilde{\sigma }}^{r+s}(BA)={\tilde{\sigma }}^r(B){\tilde{\sigma }}^s(A), \end{aligned}$$

provided at least one operator is properly supported. Moreover, $A^t\in {\tilde{\Psi }}^s(F',E')$ and

113 $$\begin{aligned} {\tilde{\sigma }}^s(A^t)={\tilde{\sigma }}^s(A)^t. \end{aligned}$$

These two properties follow immediately from the corresponding statements in the ungraded case, see Proposition 3.1(d) &(e). Recall that the bundle $E'=E^*\otimes |\Lambda |_{M}$ is equipped with the dual filtration as explained in Sect. 4.1.

For trivially filtered vector bundles these concepts clearly reduce to the ungraded case discussed in Sect. 3.1. Moreover, for differential operators we recover the graded Heisenberg order and graded Heisenberg symbol from Sect. 4.1. More precisely, for every non-negative integer *k*, we have ${\mathcal {D}}{\mathcal {O}}(E,F)\cap {\tilde{\Psi }}^k(E,F)={\widetilde{{\mathcal {D}}{\mathcal {O}}}}^k(E,F)$, and the graded Heisenberg principal symbol from Sect. 4.1 coincides with principal Heisenberg cosymbol introduced in this section via the canonical inclusion

114 $$\begin{aligned} \bigl ({\mathcal {U}}(\mathfrak tM)\otimes \hom ({{\,\mathrm{gr}\,}}(E),{{\,\mathrm{gr}\,}}(F))\bigr )_{-k}\subseteq {\tilde{\Sigma }}^k(E,F), \end{aligned}$$

see Proposition 3.1(f) and (27).

#### Lemma 5.1

Let *E* be a filtered vector bundle over a filtered manifold *M*, and let ${\mathbf {E}}$ denote the same vector bundle equipped with the trivial filtration, ${\mathbf {E}}={\mathbf {E}}^0\supseteq {\mathbf {E}}^1=0$. For every complex number *s* there exist ${\tilde{\Lambda }}_s\in {\tilde{\Psi }}_\text {prop}^s(E,{\mathbf {E}})$ and ${\tilde{\Lambda }}_s'\in {\tilde{\Psi }}^{-s}_\text {prop}({\mathbf {E}},E)$ such that ${\tilde{\Lambda }}_s{\tilde{\Lambda }}_s'-{{\,\mathrm{id}\,}}$ and ${\tilde{\Lambda }}_s'{\tilde{\Lambda }}_s-{{\,\mathrm{id}\,}}$ are both smoothing operators. Moreover, these operators may be chosen such that ${\tilde{\Lambda }}_s:\Gamma ^{-\infty }_c(E)\rightarrow \Gamma ^{-\infty }_c({\mathbf {E}})$ and ${\tilde{\Lambda }}_s':\Gamma ^{-\infty }_c({\mathbf {E}})\rightarrow \Gamma ^{-\infty }_c(E)$ are injective. On a closed manifold these operators may even be chosen such that ${\tilde{\Lambda }}_s{\tilde{\Lambda }}_s'={{\,\mathrm{id}\,}}$ and ${\tilde{\Lambda }}_s'{\tilde{\Lambda }}_s={{\,\mathrm{id}\,}}$.

#### Proof

Choose a splitting of the filtration, $S:{{\,\mathrm{gr}\,}}(E)\rightarrow E$. Let $\Lambda _{s-p}\in \Psi ^{s-p}_\text {prop}({{\,\mathrm{gr}\,}}_p(E))$ and $\Lambda _{s-p}'\in \Psi ^{-(s-p)}_\text {prop}({{\,\mathrm{gr}\,}}_p(E))$ be as in Lemma 3.13. Then, the operators

$$\begin{aligned} {\tilde{\Lambda }}_s:=S\bigl (\textstyle \bigoplus _p\Lambda _{s-p}\bigr )S^{-1} \qquad \text {and}\qquad {\tilde{\Lambda }}_s':=S\bigl (\textstyle \bigoplus _p\Lambda '_{s-p}\bigr )S^{-1} \end{aligned}$$

have the desired properties. $\square$

### Graded Heisenberg Sobolev Scale

Let *E* be a filtered vector bundle over a filtered manifold *M*. For each real number *s* we let ${\tilde{H}}^s_\text {loc}(E)$ denote the space of all distributional sections $\psi \in \Gamma ^{-\infty }(E)$ such that $A\psi \in L^2_\text {loc}({\mathbf {F}})$ for all $A\in {\tilde{\Psi }}^s_\text {prop}(E,{\mathbf {F}})$ and all trivially filtered vector bundles ${\mathbf {F}}$ over *M*, that is, ${\mathbf {F}}={\mathbf {F}}^0\supseteq {\mathbf {F}}^1=0$. We equip ${\tilde{H}}^s_\text {loc}(E)$ with the coarsest topology such that $A:{\tilde{H}}^s_\text {loc}(E)\rightarrow L^2_\text {loc}({\mathbf {F}})$ is continuous for all such $A\in {\tilde{\Psi }}^s_\text {prop}(E,{\mathbf {F}})$. Similarly, we let ${\tilde{H}}^s_c(E)$ denote the space of all compactly supported distributional sections $\psi \in \Gamma _c^{-\infty }(E)$ such that $A\psi \in L^2_\text {loc}({\mathbf {F}})$ for all $A\in {\tilde{\Psi }}^s(E,{\mathbf {F}})$ and all trivially filtered vector bundles ${\mathbf {F}}$ over *M*. We equip ${\tilde{H}}^s_c(E)$ with the coarsest topology such that $A:{\tilde{H}}^s_c(E)\rightarrow L^2_\text {loc}({\mathbf {F}})$ is continuous for all such $A\in {\tilde{\Psi }}^s(E,{\mathbf {F}})$. We will refer to these spaces as *graded Heisenberg Sobolev spaces*. Any splitting of the filtration on *E* gives rise to non-canonical topological isomorphisms

$$\begin{aligned} {\tilde{H}}^s_\text {loc}(E)\cong \bigoplus _p H^{s-p}_\text {loc}\bigl ({{\,\mathrm{gr}\,}}_p(E)\bigr ) \qquad \text {and}\qquad {\tilde{H}}^s_c(E)\cong \bigoplus _pH^{s-p}_c\bigl ({{\,\mathrm{gr}\,}}_p(E)\bigr ). \end{aligned}$$

Generalizing Proposition 3.17(a) &(b), we have continuous inclusions

$$\begin{aligned} \Gamma ^\infty (E)\subseteq {\tilde{H}}^{s_2}_\text {loc}(E)\subseteq {\tilde{H}}^{s_1}_\text {loc}(E)\subseteq \Gamma ^{-\infty }(E) \end{aligned}$$

and

$$\begin{aligned} \Gamma ^\infty _c(E)\subseteq {\tilde{H}}^{s_2}_c(E)\subseteq {\tilde{H}}^{s_1}_c(E)\subseteq \Gamma _c^{-\infty }(E) \end{aligned}$$

for all real numbers $s_1\le s_2$. If *F* is another filtered vector bundle, then each $A\in {\tilde{\Psi }}^k(E,F)$ induces continuous operators $A:{\tilde{H}}^s_c(E)\rightarrow {\tilde{H}}_\text {loc}^{s-\Re (k)}(F)$ for all real *s*, cf. Proposition 3.17(d). As in Proposition 3.17(c), the canonical pairing $\Gamma ^\infty _c(E')\times \Gamma ^\infty (E)\rightarrow {\mathbb {C}}$ extends to a pairing

$$\begin{aligned} {\tilde{H}}^{-s}_c(E')\times {\tilde{H}}^s_\text {loc}(E)\rightarrow {\mathbb {C}}\end{aligned}$$

inducing linear bijections ${\tilde{H}}^s_\text {loc}(E)^*={\tilde{H}}^{-s}_c(E')$ and ${\tilde{H}}^{-s}_c(E')^*={\tilde{H}}^s_\text {loc}(E)$. If, moreover, *M* is closed, then ${\tilde{H}}^s_c(E)={\tilde{H}}^s_\text {loc}(E)$ is a Hilbert space we denote by ${\tilde{H}}^s(E)$, and the pairing induces an isomorphism of Hilbert spaces, ${\tilde{H}}^s(E)^*={\tilde{H}}^{-s}(E')$. This can all be proved as in Proposition 3.17 using Lemma 5.1.

Suppose *M* is closed. Fix a smooth volume density on *M* and a smooth fiber-wise Hermitian metric *h* on ${\mathbf {E}}$. Moreover, let *s* be a real number, choose invertible ${\tilde{\Lambda }}_s\in {\tilde{\Psi }}^s(E,{\mathbf {E}})$ with inverse ${\tilde{\Lambda }}_s^{-1}\in {\tilde{\Psi }}^{-s}({\mathbf {E}},E)$, see Lemma 5.1, and consider the associated Hermitian inner product, cf. (11),

115 $$\begin{aligned} \langle \!\langle \psi _1,\psi _2\rangle \!\rangle _{{\tilde{H}}^s(E)} :=\langle \!\langle {\tilde{\Lambda }}_s\psi _1,{\tilde{\Lambda }}_s\psi _2\rangle \!\rangle _{L^2({\mathbf {E}})} =\langle {\tilde{\Lambda }}_s^t(h\otimes dx){\tilde{\Lambda }}_s\psi _1,\psi _2\rangle \end{aligned}$$

where $\psi _1,\psi _2\in \Gamma ^\infty (E)$. In the expression on the right hand side $h\otimes dx:\bar{{\mathbf {E}}}\rightarrow {\mathbf {E}}'$ is considered as a vector bundle isomorphism, ${\tilde{\Lambda }}_s^t\in {\tilde{\Psi }}^s({\mathbf {E}}',E')$, and $\langle -,-\rangle$ denotes the canonical pairing for sections of *E*. The sesquilinear form in (115) extends to an inner product generating the Hilbert space topology on the graded Heisenberg Sobolev space ${\tilde{H}}^s(E)$.

With respect to inner products on ${\tilde{H}}^{s_1}(E)$ and ${\tilde{H}}^{s_2}(F)$ as above, every $A\in {\tilde{\Psi }}^k(E,F)$ admits a formal adjoint, $A^\sharp \in {\tilde{\Psi }}^{{\bar{k}}+2(s_2-s_1)}(F,E)$ such that

116 $$\begin{aligned} \langle \!\langle A^\sharp \phi ,\psi \rangle \!\rangle _{{\tilde{H}}^{s_1}(E)}=\langle \!\langle \phi ,A\psi \rangle \!\rangle _{{\tilde{H}}^{s_2}(F)} \end{aligned}$$

for all $\psi \in \Gamma ^\infty (E)$ and $\phi \in \Gamma ^\infty (F)$. Indeed,

117 $$\begin{aligned} A^\sharp ={\tilde{\Lambda }}_{E,s_1}^{-1}({\tilde{\Lambda }}_{F,s_2}A{\tilde{\Lambda }}_{E,s_1}^{-1})^*{\tilde{\Lambda }}_{F,s_2} =({\tilde{\Lambda }}_{E,s_1}^t(h_E\otimes dx){\tilde{\Lambda }}_{E,s_1})^{-1}\,A^t\,{\tilde{\Lambda }}_{F,s_2}^t(h_F\otimes dx){\tilde{\Lambda }}_{F,s_2}.\nonumber \\ \end{aligned}$$

In the first expression the star denotes the adjoint of ${\tilde{\Lambda }}_{F,s_2}A{\tilde{\Lambda }}_{E,s_1}^{-1}\in \Psi ^{k+s_2-s_1}({\mathbf {E}},{\mathbf {F}})$ with respect to the $L^2$ inner products associated with the fiber-wise Hermitian metrics $h_E$ and $h_F$ and the volume density *dx*. One readily verifies:

118 $$\begin{aligned} (BA)^\sharp =A^\sharp B^\sharp \qquad \text {and}\qquad (A^\sharp )^\sharp =A. \end{aligned}$$

### Graded Rockland operators

Let *E* and *F* be filtered vector bundles over a filtered manifold *M*. To formulate the graded Rockland condition for operators in ${\tilde{\Psi }}^s(E,F)$, we begin by extending the definition of ${\bar{\pi }}(a)$ to graded cosymbols $a\in {\tilde{\Sigma }}^s_x(E,F)$ at $x\in M$, where $\pi :{\mathcal {T}}_xM\rightarrow U({\mathcal {H}})$ is a non-trivial irreducible unitary representation of the osculating group: Write $a=\sum _{p,q}a_{p,q}$ according to the decomposition (111) with $a_{q,p}\in \Sigma ^{s+q-p}_x({{\,\mathrm{gr}\,}}_p(E),{{\,\mathrm{gr}\,}}_q(F))$, and put ${\bar{\pi }}(a):=\sum _{p,q}{\bar{\pi }}(a_{q,p})$ where ${\bar{\pi }}(a_{q,p})$ denotes the unbounded operator from ${\mathcal {H}}\otimes {{\,\mathrm{gr}\,}}_p(E_x)$ to ${\mathcal {H}}\otimes {{\,\mathrm{gr}\,}}_q(F_x)$ described in Sect. 3.2. Hence, ${\bar{\pi }}(a)$ is an unbounded operator form ${\mathcal {H}}\otimes {{\,\mathrm{gr}\,}}(E_x)$ to ${\mathcal {H}}\otimes {{\,\mathrm{gr}\,}}(F_x)$. Moreover, the subspace ${\mathcal {H}}_\infty \otimes {{\,\mathrm{gr}\,}}(E_x)$ is contained in the domain of definition and mapped into ${\mathcal {H}}_\infty \otimes {{\,\mathrm{gr}\,}}(F_x)$. From (34) we immediately obtain

119 $$\begin{aligned} {\bar{\pi }}(ba)={\bar{\pi }}(b){\bar{\pi }}(a) \end{aligned}$$

for all $a\in {\tilde{\Sigma }}^s_x(E,F)$ and $b\in {\tilde{\Sigma }}^{s'}_x(F,G)$. For trivially filtered vector bundles, this clearly specializes to the definition in Sect. 3.2. If *k* is a non-negative integer and, see (114), $a\in \bigl ({\mathcal {U}}({\mathfrak {t}}_xM)\otimes \hom ({{\,\mathrm{gr}\,}}(E_x),{{\,\mathrm{gr}\,}}(F_x))\bigr )_{-k}\subseteq {\tilde{\Sigma }}^k_x(E,F)$ then, on ${\mathcal {H}}_\infty \otimes {{\,\mathrm{gr}\,}}(E_x)$, the operator ${\bar{\pi }}(a)$ coincides with $\pi (a)$ considered in Sect. 4.1, cf. Definition 4.2.

Generalizing Definition 3.8 to the graded situation we have:

#### Definition 5.2

(*Graded Rockland condition*) Let *E* and *F* be filtered vector bundles over a filtered manifold *M*. A graded principal cosymbol $a\in {\tilde{\Sigma }}_x^s(E,F)$ at $x\in M$ is said to satisfy the *graded Rockland condition* if, for every non-trivial irreducible unitary representation $\pi :{\mathcal {T}}_xM\rightarrow U({\mathcal {H}})$, the unbounded operator ${\bar{\pi }}(a)$ is injective on ${\mathcal {H}}_\infty \otimes {{\,\mathrm{gr}\,}}(E_x)$. An operator $A\in {\tilde{\Psi }}^s(E,F)$ is said to satisfy the *graded Rockland condition* if its graded principal cosymbol, ${\tilde{\sigma }}^s_x(A)\in {\tilde{\Sigma }}^s_x(E,F)$, satisfies the graded Rockland condition at each point $x\in M$.

We obtain the following generalization of Theorem 3.11 and Corollary 3.20.

#### Corollary 5.3

(Left parametrix and graded regularity) Let *E* and *F* be two filtered vector bundles over a filtered manifold *M*, let *k* be a complex number, and suppose $A\in {\tilde{\Psi }}^k(E,F)$ satisfies the graded Rockland condition. Then, there exists a properly supported left parametrix $B\in {\tilde{\Psi }}^{-k}_\text {prop}(F,E)$ such that $BA-{{\,\mathrm{id}\,}}$ is a smoothing operator. In particular, *A* is hypoelliptic. More precisely, if $\psi \in \Gamma ^{-\infty }_c(E)$ and $A\psi \in {\tilde{H}}^{r-\Re (k)}_\text {loc}(F)$, then $\psi \in {\tilde{H}}^r_c(E)$. If, moreover, *M* is closed, then $\ker (A)$ is a finite dimensional subspace of $\Gamma ^\infty (E)$, and for every $r'\le r$ there exists a constant $C=C_{A,r,r'}\ge 0$ such that the maximal graded hypoelliptic estimate

120 $$\begin{aligned} \Vert \psi \Vert _{{\tilde{H}}^r(E)}\le C\left( \Vert \psi \Vert _{{\tilde{H}}^{r'}(E)}+\Vert A\psi \Vert _{{\tilde{H}}^{r-\Re (k)}(F)}\right) \end{aligned}$$

holds for all $\psi \in {\tilde{H}}^r(E)$. Here we are using any norms generating the Hilbert space topologies on the corresponding graded Heisenberg Sobolev spaces. Moreover, if *Q* denotes the orthogonal projection, with respect to an inner product of the form (115), onto the (finite dimensional) subspace $\ker (A)\subseteq \Gamma ^\infty (E)$, then there exists a constant $C=C_{A,r,s}\ge 0$ such that the maximal graded hypoelliptic estimate

121 $$\begin{aligned} \Vert \psi \Vert _{{\tilde{H}}^r(E)}\le C\left( \Vert Q\psi \Vert +\Vert A\psi \Vert _{{\tilde{H}}^{r-\Re (k)}(F)}\right) \end{aligned}$$

holds for all $\psi \in {\tilde{H}}^r(E)$. Here $\Vert -\Vert$ denotes any norm on $\ker (A)$.

#### Proof

Let ${\mathbf {E}}$ and ${\mathbf {F}}$ denote the vector bundles *E* and *F* equipped with the trivial filtrations, respectively, that is to say, ${\mathbf {E}}={\mathbf {E}}^0\supseteq {\mathbf {E}}^1=0$ and ${\mathbf {F}}={\mathbf {F}}^0\supseteq {\mathbf {F}}^1=0$. According to Lemma 5.1 there exist ${\tilde{\Lambda }}_E\in {\tilde{\Psi }}^0(E,{\mathbf {E}})$, ${\tilde{\Lambda }}_E'\in {\tilde{\Psi }}^0({\mathbf {E}},E)$, ${\tilde{\Lambda }}_F\in {\tilde{\Psi }}^0(F,{\mathbf {F}})$, ${\tilde{\Lambda }}_F'\in {\tilde{\Psi }}^0({\mathbf {F}},F)$ such that ${\tilde{\Lambda }}_E{\tilde{\Lambda }}_E'-{{\,\mathrm{id}\,}}$, ${\tilde{\Lambda }}_E'{\tilde{\Lambda }}_E-{{\,\mathrm{id}\,}}$, ${\tilde{\Lambda }}_F{\tilde{\Lambda }}_F'-{{\,\mathrm{id}\,}}$, and ${\tilde{\Lambda }}_F'{\tilde{\Lambda }}_F-{{\,\mathrm{id}\,}}$ are all smoothing operators. Then ${\mathbf {A}}:={\tilde{\Lambda }}_FA{\tilde{\Lambda }}_E'\in \Psi ^k({\mathbf {E}},{\mathbf {F}})$ has Heisenberg order *k* in the ungraded sense, and

$$\begin{aligned} \sigma _x^k({\mathbf {A}})={\tilde{\sigma }}^k_x({\mathbf {A}})={\tilde{\sigma }}^0_x({\tilde{\Lambda }}_F){\tilde{\sigma }}^k_x(A){\tilde{\sigma }}^0_x({\tilde{\Lambda }}_E'), \end{aligned}$$

see (112). Since *A* satisfies the graded Rockland condition, and since ${\tilde{\sigma }}^0_x({\tilde{\Lambda }}_F)$ and ${\tilde{\sigma }}^0_x({\tilde{\Lambda }}_E')$ are invertible with inverses ${\tilde{\sigma }}^0_x({\tilde{\Lambda }}_F')$ and ${\tilde{\sigma }}^0_x({\tilde{\Lambda }}_E)$, respectively, we conclude that ${\mathbf {A}}$ satisfies the (ungraded) Rockland condition, see (119). Hence, by Theorem 3.11, there exists a left parametrix ${\mathbf {B}}\in \Psi ^{-k}_\text {prop}({\mathbf {F}},{\mathbf {E}})$ such that ${\mathbf {B}}{\mathbf {A}}-{{\,\mathrm{id}\,}}$ is a smoothing operator. Putting $B:={\tilde{\Lambda }}_E'{\mathbf {B}}{\tilde{\Lambda }}_F\in {\tilde{\Psi }}^{-k}(F,E)$ and using the fact that ${\tilde{\Lambda }}_E'{\tilde{\Lambda }}_E-{{\,\mathrm{id}\,}}$ is a smoothing operator, we see that $BA-{{\,\mathrm{id}\,}}$ is a smoothing operator. Hence, *B* is the desired left parametrix. The hypoellipticity statements follow immediately from the pseudolocality of *B* and the mapping property $B:{\tilde{H}}_\text {loc}^{r-\Re (k)}(F)\rightarrow {\tilde{H}}^r_\text {loc}(E)$. Assume *M* closed. As in Corollary 2.8 we see that $\ker (A)$ is a finite dimensional subspace of $\Gamma ^\infty (E)$. For the maximal graded hypoelliptic estimate (120) we use boundedness of $B:{\tilde{H}}^{r-\Re (k)}(F)\rightarrow {\tilde{H}}^r(E)$ and the fact that smoothing operators induce bounded operators ${\tilde{H}}^{r'}(E)\rightarrow {\tilde{H}}^r(E)$. To see the other hypoelliptic estimate, we consider the formal adjoint $A^\sharp \in {\tilde{\Psi }}^{{\bar{k}}}(F,E)$ with respect to inner products of the form (115), see (117) with $s_1=s_2=s$. Clearly, $A^\sharp A\in {\tilde{\Psi }}^{2\Re (k)}(E)$ satisfies the graded Rockland condition and $\ker (A^\sharp A)=\ker (A)$. Hence, Corollary 5.4 implies that $A^\sharp A+Q$ is invertible with inverse $(A^\sharp A+Q)^{-1}\in {\tilde{\Psi }}^{-2\Re (k)}(E)$. Thus, $B':=(A^\sharp A+Q)^{-1}A^\sharp \in {\tilde{\Psi }}^{-k}(F,E)$ is a parametrix such that $B'A={{\,\mathrm{id}\,}}-Q$, whence (121). $\square$

In view of Corollary 5.3, the graded Rockland condition implies Rumin’s C–C ellipticity, cf. [69, Definition 5.1] or [67, Section 2].

We obtain the following generalization of Corollaries 2.9, 3.12, and 3.22. A Hodge decomposition for the Rumin complex on an equiregular C–C manifold has been established in [68, Proposition 3.6].

#### Corollary 5.4

(Graded Hodge decomposition) Let *E* be a filtered vector bundle over a closed filtered manifold *M*. Suppose $A\in {\tilde{\Psi }}^k(E)$ satisfies the graded Rockland condition and is formally self-adjoint, $A^\sharp =A$, with respect to a graded Sobolev inner product of the form (115). Moreover, let *Q* denotes the orthogonal projection onto the (finite dimensional) subspace $\ker (A)\subseteq \Gamma ^\infty (E)$ with respect to the inner product (115). Then, $A+Q$ is invertible with inverse $(A+Q)^{-1}\in {\tilde{\Psi }}^{-k}(E)$. Consequently, we have topological isomorphisms and Hodge type decompositions:

$$\begin{aligned} A+Q:\Gamma ^\infty (E)&\xrightarrow \cong \Gamma ^\infty (E)&\Gamma ^\infty (E)&=\ker (A)\oplus A(\Gamma ^\infty (E))\\ A+Q:{\tilde{H}}^r(E)&\xrightarrow \cong {\tilde{H}}^{r-\Re (k)}(E)&{\tilde{H}}^{r-\Re (k)}(E)&=\ker (A)\oplus A({\tilde{H}}^r(E))\\ A+Q:\Gamma ^{-\infty }(E)&\xrightarrow \cong \Gamma ^{-\infty }(E)&\Gamma ^{-\infty }(E)&=\ker (A)\oplus A(\Gamma ^{-\infty }(E)) \end{aligned}$$

#### Proof

Let ${\mathbf {E}}$ denote the vector bundle *E* equipped with the trivial filtration, ${\mathbf {E}}={\mathbf {E}}^0\supseteq {\mathbf {E}}^1=0$. Recall that $A^\sharp ={\tilde{\Lambda }}_s^{-1}({\tilde{\Lambda }}_sA{\tilde{\Lambda }}_s^{-1})^*{\tilde{\Lambda }}_s$, see (117). Hence, the assumption $A^\sharp =A$ implies that ${\mathbf {A}}:={\tilde{\Lambda }}_sA{\tilde{\Lambda }}_s^{-1}\in \Psi ^k({\mathbf {E}})$ is formally self-adjoint with respect to the $L^2$ inner product (11), that is, ${\mathbf {A}}^*={\mathbf {A}}$. Moreover, ${\mathbf {A}}$ satisfies the (ungraded) Rockland condition for ${\tilde{\sigma }}^s_x({\tilde{\Lambda }}_s)$ is invertible with inverse ${\tilde{\sigma }}^{-s}_x({\tilde{\Lambda }}_s^{-1})$, see (112). Hence, according to Corollary 3.12, ${\mathbf {A}}+{\mathbf {Q}}\in \Psi ^k({\mathbf {E}})$ is invertible with inverse $({\mathbf {A}}+{\mathbf {Q}})^{-1}\in \Psi ^{-k}({\mathbf {E}})$, where ${\mathbf {Q}}\in {\mathcal {O}}^{-\infty }({\mathbf {E}})$ denotes the orthogonal projection onto $\ker ({\mathbf {A}})$, a finite dimensional subspace of $\Gamma ^\infty ({\mathbf {E}})$. Note that $Q:={\tilde{\Lambda }}_s^{-1}{\mathbf {Q}}{\tilde{\Lambda }}_s\in {\mathcal {O}}^{-\infty }(E)$ is the orthogonal projection onto $\ker (A)$ with respect to the inner product (115). Conjugating with ${\tilde{\Lambda }}_s$, we conclude that $A+Q\in {\tilde{\Psi }}^k(E)$ is invertible with inverse $(A+Q)^{-1}={\tilde{\Lambda }}_s^{-1}({\mathbf {A}}+{\mathbf {Q}})^{-1}{\tilde{\Lambda }}_s\in {\tilde{\Psi }}^{-k}(E)$. The remaining assertions follow at once. $\square$

We have the following generalization of Corollary 3.23:

#### Corollary 5.5

(Fredholm operators and index) Let *E* and *F* be a filtered vector bundles over a closed filtered manifold *M*. Suppose $A\in {\tilde{\Psi }}^k(E,F)$ is such that *A* and $A^t$ both satisfy the graded Rockland condition. Then, for every real number *r*, we have an induced Fredholm operator $A:{\tilde{H}}^r(E)\rightarrow {\tilde{H}}^{r-\Re (k)}(F)$ whose index is independent of *r* and can be expressed as

$$\begin{aligned} {{\,\mathrm{ind}\,}}(A)=\dim \ker A-\dim \ker A^t. \end{aligned}$$

This index depends only on the graded Heisenberg principal cosymbol ${\tilde{\sigma }}^k(A)\in {\tilde{\Sigma }}^k(E,F)$.

#### Proof

Using Corollary 5.3 we obtain a parametrix $B\in {\tilde{\Psi }}^{-k}(F,E)$ such that $BA-{{\,\mathrm{id}\,}}$ and $AB-{{\,\mathrm{id}\,}}$ are both smoothing operators, cf. Remark 3.15. We may now proceed exactly as in the proof of Corollary 3.23. $\square$

### Graded Rockland sequences

Throughout this section we assume *M* to be a closed filtered manifold. Suppose $E_i$ are filtered vector bundles over *M*, and consider a sequence

122 $$\begin{aligned} \cdots \rightarrow \Gamma ^\infty (E_{i-1})\xrightarrow {A_{i-1}}\Gamma ^\infty (E_i)\xrightarrow {A_i}\Gamma ^\infty (E_{i+1})\rightarrow \cdots \end{aligned}$$

where $A_i\in {\tilde{\Psi }}^{k_i}(E_i,E_{i+1})$ for some complex numbers $k_i$. Generalizing Definition 4.2 for sequences of differential operators, we make the following

#### Definition 5.6

(Graded Rockland sequence) A sequence of operators as above is said to be a *graded Rockland sequence* if, for every $x\in M$ and every non-trivial irreducible unitary representation $\pi :{\mathcal {T}}_xM\rightarrow U({\mathcal {H}})$, the sequence

$$\begin{aligned} \cdots \rightarrow {\mathcal {H}}_\infty \otimes {{\,\mathrm{gr}\,}}(E_{{i-1,x}})\xrightarrow {{\bar{\pi }}({\tilde{\sigma }}^{k_{i-1}}_x(A_{i-1}))} {\mathcal {H}}_\infty \otimes {{\,\mathrm{gr}\,}}(E_{i,x})\xrightarrow {{\bar{\pi }}({\tilde{\sigma }}^{k_i}_x(A_i))} {\mathcal {H}}_\infty \otimes {{\,\mathrm{gr}\,}}(E_{i+1,x})\rightarrow \cdots \end{aligned}$$

is weakly exact; that is, the image of each arrow is contained and dense in the kernel of the subsequent arrow. Here ${\mathcal {H}}_\infty$ denotes the subspace of smooth vectors in ${\mathcal {H}}$.

Suppose the sequence in (122) is a Rockland sequence. Fix real numbers $s_i$ such that $\Re (k_i)+s_{i+1}-s_i$ is independent of *i*, and put

123 $$\begin{aligned} \kappa :=\Re (k_i)+s_{i+1}-s_i. \end{aligned}$$

Let ${\mathbf {E}}_i$ denote the vector bundle $E_i$ considered as a trivially filtered bundle, that is, equipped with the filtration ${\mathbf {E}}_i={\mathbf {E}}_i^0\supseteq {\mathbf {E}}^1_i=0$. Fix a smooth volume density on *M* as well as smooth fiber-wise Hermitian inner products $h_i$ on ${\mathbf {E}}_i$ and let $\langle \!\langle -,-\rangle \!\rangle _{L^2({\mathbf {E}}_i)}$ denote the associated $L^2$ inner product on sections of ${\mathbf {E}}_i$, see (11). Moreover, choose invertible ${\tilde{\Lambda }}_i\in {\tilde{\Psi }}^{s_i}(E_i,{\mathbf {E}}_i)$ with ${\tilde{\Lambda }}_i^{-1}\in {\tilde{\Psi }}^{-s_i}({\mathbf {E}}_i,E_i)$, see Lemma 5.1, and consider the associated graded Sobolev inner product on sections of $E_i$,

124 $$\begin{aligned} \langle \!\langle \psi _1,\psi _2\rangle \!\rangle _{{\tilde{H}}^{s_i}(E_i)} :=\langle \!\langle {\tilde{\Lambda }}_i\psi _1,{\tilde{\Lambda }}_i\psi \rangle \!\rangle _{L^2({\mathbf {E}}_i)} =\langle {\tilde{\Lambda }}_i^t(h_i\otimes dx){\tilde{\Lambda }}_i\psi _1,\psi _2\rangle \end{aligned}$$

where $$\psi _1,\psi _2\in \Gamma ^\infty (E_i)$$. Let $$A_i^\sharp \in {\tilde{\Psi }}^{2\kappa -k_i}(E_{i+1},E_i)$$,

$$\begin{aligned} A_i^\sharp ={\tilde{\Lambda }}_i^{-1}({\tilde{\Lambda }}_{i+1}A_i{\tilde{\Lambda }}_i^{-1})^*{\tilde{\Lambda }}_{i+1} =({\tilde{\Lambda }}_i^t(h_i\otimes dx){\tilde{\Lambda }}_i)^{-1}\,A^t_i\,{\tilde{\Lambda }}_{i+1}^t(h_{i+1}\otimes dx){\tilde{\Lambda }}_{i+1} \end{aligned}$$

denote the formal adjoint of $A_i$ with respect to these inner products, that is,

$$\begin{aligned} \langle \!\langle A_i^\sharp \phi ,\psi \rangle \!\rangle _{{\tilde{H}}^{s_i}(E_i)}=\langle \!\langle \phi ,A_i\psi \rangle \!\rangle _{{\tilde{H}}^{s_{i+1}}(E_{i+1})} \end{aligned}$$

for all $\psi \in \Gamma ^\infty (E_i)$ and $\phi \in \Gamma ^\infty (E_{i+1})$. Let us finally consider the Laplace type operators

$$\begin{aligned} B_i:=A_{i-1}A_{i-1}^\sharp +A_i^\sharp A_i. \end{aligned}$$

Note that $B_i=B_i^\sharp \in {\tilde{\Psi }}^{2\kappa }(E_i)$, see (123) and (118).

#### Lemma 5.7

The operator $B_i$ satisfies the graded Rockland condition.

#### Proof

Note that ${\mathbf {B}}_i:={\tilde{\Lambda }}_iB_i{\tilde{\Lambda }}_i^{-1}\in \Psi ^{2\kappa }({\mathbf {E}}_i)$ is of the form ${\mathbf {B}}_i={\mathbf {A}}_i^*{\mathbf {A}}_i+{\mathbf {A}}_{i-1}{\mathbf {A}}_{i-1}^*$ where ${\mathbf {A}}_i:={\tilde{\Lambda }}_{i+1}A_i{\tilde{\Lambda }}_i^{-1}\in \Psi ^{k_i+s_{i+1}-s_i}({\mathbf {E}}_i,{\mathbf {E}}_{i+1})$. Using (34), (35), and Remark 3.3, we obtain

$$\begin{aligned} {\bar{\pi }}(\sigma ^{2\kappa }_x({\mathbf {B}}_i))= & {} {\bar{\pi }}(\sigma ^{k_{i-1}+s_i-s_{i-1}}_x({\mathbf {A}}_{i-1})){\bar{\pi }}(\sigma ^{k_{i-1}+s_i-s_{i-1}}_x({\mathbf {A}}_{i-1}))^* \\&+{\bar{\pi }}(\sigma ^{k_i+s_{i+1}-s_i}_x({\mathbf {A}}_i))^*{\bar{\pi }}(\sigma ^{k_i+s_{i+1}-s_i}_x({\mathbf {A}}_i)). \end{aligned}$$

Since the operators ${\mathbf {A}}_i$ form an (ungraded) Rockland sequence, one readily concludes that ${\mathbf {B}}_i$ satisfies the (ungraded) Rockland condition, cf. the proof of Lemma 2.14. Clearly, this implies that $B_i$ satisfies the graded Rockland condition, see (119). $\square$

In view of Lemma 5.7, Corollary 5.4 applies to each of the operators $B_i$ and we obtain the following generalization of Corollaries 2.15, 2.16, 3.24, and 3.25:

#### Corollary 5.8

The operator $(A_{i-1}^\sharp ,A_i)$ is hypoelliptic. More precisely, if $\psi \in \Gamma ^{-\infty }(E_i)$ is such that $A_{i-1}^\sharp \psi \in {\tilde{H}}^{r-2\kappa +\Re (k_{i-1})}(E_{i-1})$ and $A_i\psi \in {\tilde{H}}^{r-\Re (k_i)}(E_{i+1})$, then $\psi \in {\tilde{H}}^r(E_i)$. Moreover, there exists a constant $C=C_{A_i,r}\ge 0$ such that the maximal graded hypoelliptic estimate

$$\begin{aligned} \Vert \psi \Vert _{{\tilde{H}}^r(E_i)}\le C\left( \Vert A^\sharp _{i-1}\psi \Vert _{{\tilde{H}}^{r-2\kappa +\Re (k_{i-1})}(E_{i-1})}+\Vert Q_i\psi \Vert _{\ker (B_i)}+\Vert A_i\psi \Vert _{{\tilde{H}}^{r-\Re (k_i)}(E_{i+1})}\right) \end{aligned}$$

holds for all $\psi \in {\tilde{H}}^r(E_i)$. Here *r* is any real number, $Q_i$ denotes the orthogonal projection onto the (finite dimensional) subspace $\ker (B_i)\subseteq \Gamma ^\infty (E_i)$ with respect to the inner product (124), $\Vert -\Vert _{\ker (B_i)}$ denotes any norm on $\ker (B_i)$, and $\Vert -\Vert _{{\tilde{H}}^r(E_i)}$ is any norm generating the Hilbert space topology on the graded Sobolev space ${\tilde{H}}^r(E_i)$. Furthermore,

$$\begin{aligned} \ker (B_i)=\ker (A_{i-1}^\sharp |_{\Gamma ^{-\infty }(E_i)})\cap \ker (A_i|_{\Gamma ^{-\infty }(E_i)}) =\ker (A_{i-1}^\sharp |_{\Gamma ^\infty (E_i)})\cap \ker (A_i|_{\Gamma ^\infty (E_i)}). \end{aligned}$$

If, moreover, $A_iA_{i-1}=0$, then we have Hodge type decompositions

$$\begin{aligned} \Gamma ^\infty (E_i)&=A_{i-1}(\Gamma ^\infty (E_{i-1}))\oplus \ker (B_i)\oplus A_i^\sharp (\Gamma ^\infty (E_{i+1}))\\ {\tilde{H}}^r(E_i)&=A_{i-1}\bigl ({\tilde{H}}^{r+\Re (k_{i-1})}(E_{i-1})\bigr )\oplus \ker (B_i)\oplus A_i^\sharp \bigl ({\tilde{H}}^{2\kappa -\Re (k_i)}(E_{i+1})\bigr )\\ \Gamma ^{-\infty }(E_i)&=A_{i-1}(\Gamma ^{-\infty }(E_{i-1}))\oplus \ker (B_i)\oplus A_i^\sharp (\Gamma ^{-\infty }(E_{i+1})) \end{aligned}$$

as well as:

$$\begin{aligned} \ker (A_i|_{\Gamma ^\infty (E_i)})&=A_{i-1}(\Gamma ^\infty (E_{i-1}))\oplus \ker (B_i)\\ \ker (A_i|_{{\tilde{H}}^r(E_i)})&=A_{i-1}({\tilde{H}}^{r+\Re (k_{i-1})}(E_{i-1}))\oplus \ker (B_i)\\ \ker (A_i|_{\Gamma ^{-\infty }(E_i)})&=A_{i-1}(\Gamma ^{-\infty }(E_{i-1}))\oplus \ker (B_i) \end{aligned}$$

In particular, every cohomology class admits a unique harmonic representative:

$$\begin{aligned} \frac{\ker (A_i|_{\Gamma ^{-\infty }(E_i)})}{{{\,\mathrm{img}\,}}(A_{i-1}|_{\Gamma ^{-\infty }(E_{i-1})})} =\frac{\ker (A_i|_{\Gamma ^\infty (E_i)})}{{{\,\mathrm{img}\,}}(A_{i-1}|_{\Gamma ^\infty (E_{i-1})})} =\ker (B_i) =\ker (A_{i-1}^\sharp )\cap \ker (A_i). \end{aligned}$$

## Data Availability

Data sharing is not applicable to this article as no datasets were generated or analyzed during the current study.

## Footnotes

1 The opposite groupoid ${\mathcal {T}}^\text {op}M$ mediates between two conflicting, yet common, conventions we are following: The Lie algebra of a Lie group is usually defined by restricting the Lie bracket to *left invariant* vector fields, while the Lie algebroid of a smooth groupoid is defined using *right invariant* vector fields.

2 Actually, $r_0=0$ appears to be sufficient, see [16] and [59, Remark 3.3.14], but we won’t need that.

3 The degree *p* filtration subbundle of ${{\,\mathrm{gr}\,}}(E)$ is ${{\,\mathrm{gr}\,}}(E)^p=\bigoplus _{q\ge p}{{\,\mathrm{gr}\,}}_q(E)$ and thus the filtration on ${{\,\mathrm{gr}\,}}(E)'$ canonically identifies to $({{\,\mathrm{gr}\,}}(E)')^p=\bigoplus _{q\le -p}{{\,\mathrm{gr}\,}}_q(E)'=\bigoplus _{q\le -p}{{\,\mathrm{gr}\,}}_{-q}(E')={{\,\mathrm{gr}\,}}(E')^p$.

4 In subsequent sections we will restrict our attention to the case when $\delta _i$ are (tensorial) vector bundle homomorphisms satisfying $\delta _i\delta _{i+1}=0$, but these restrictions would not be helpful here.

5 In particular, $\delta _i$ is of graded Heisenberg order at most $-{k_{i-1}}$.

6 Only the existence of such splittings is required, $S_i$ is not part of the data.

7 As formulated in [10, Theorem 3.3.5], Kostant’s version of the Bott–Borel–Weil theorem computes the cohomology $H^k({\mathfrak {p}}_+;{\mathbb {E}})$. Using the following facts, this permits working out the homology $H_k({\mathfrak {p}}_+;{\mathbb {E}})$ as well: $H^k({\mathfrak {p}}_+;{\mathbb {E}}^*)\cong H_k({\mathfrak {p}}_+;{\mathbb {E}})^*$ as ${\mathfrak {g}}_0$-modules; ${\mathbb {E}}\cong {\mathbb {E}}^*$ as ${\mathfrak {g}}$-modules; If *W* is an irreducible ${\mathfrak {g}}_0$ module with highest weight $a\lambda _1+b\lambda _2$, then $W^*$ is an irreducible ${\mathfrak {g}}_0$ module with highest weight $a'\lambda _1+b'\lambda _2$, where $a'=a$ and $b'=-3a-b$.
